# Isolation and characterization of a novel bacterial strain from a Tris-Acetate-Phosphate agar medium plate of the green micro-alga *Chlamydomonas reinhardtii *that can utilize common environmental pollutants as a carbon source

**DOI:** 10.12688/f1000research.24680.1

**Published:** 2020-06-29

**Authors:** Mautusi Mitra, Kevin Manoap-Anh-Khoa Nguyen, Taylor Wayland Box, Jesse Scott Gilpin, Seth Ryan Hamby, Taylor Lynne Berry, Erin Harper Duckett

**Affiliations:** 1 Department of Biology, University of West Georgia, Carrollton, Georgia, 30118, USA; 2 Department of Mechanical Engineering, Kennesaw State University, Marietta, Georgia, 30060, USA; 3 Carrollton High School, Carrollton, Georgia, 30117, USA; 4 Department of Chemistry and Biochemistry, University of North Georgia, Dahlonega, Georgia, 30597, USA

**Keywords:** Chlamydomonas, Acidovorax sp., TAP medium, LMJ, bioremediation, 16S rRNA gene, penicillin-sensitivity, pyomelanin

## Abstract

**Background:** *Chlamydomonas reinhardtii*, a green micro-alga can be grown at the lab heterotrophically or photo-heterotrophically in Tris-Phosphate-Acetate (TAP) medium which contains acetate as the sole carbon source. When grown in TAP medium, *Chlamydomonas* can utilize the exogenous acetate in the medium for gluconeogenesis using the glyoxylate cycle, which is also present in many bacteria and higher plants. A novel bacterial strain, LMJ, was isolated from a contaminated TAP medium plate of *Chlamydomonas*. We present our work on the isolation and physiological and biochemical characterizations of LMJ.

**Methods:** Several microbiological tests were conducted to characterize LMJ, including its sensitivity to four antibiotics. We amplified and sequenced partially the 16S rRNA gene of LMJ. We tested if LMJ can utilize cyclic alkanes, aromatic hydrocarbons, poly-hydroxyalkanoates, and fresh and combusted car motor oil as the sole carbon source on Tris-Phosphate (TP) agar medium plates for growth.

**Results:** LMJ is a gram-negative rod, oxidase-positive, mesophilic, non-enteric, pigmented, salt-sensitive bacterium. LMJ can ferment glucose, is starch hydrolysis-negative, and is very sensitive to penicillin and chloramphenicol. Preliminary spectrophotometric analyses indicate LMJ produces pyomelanin. NCBI-BLAST analyses of the partial 16S rRNA gene sequence of LMJ showed that it matched to that of an uncultured bacterium clone LIB091_C05_1243. The nearest genus relative of LMJ is an *Acidovorax* sp. strain. LMJ was able to use alkane hydrocarbons, fresh and combusted car motor oil, poly-hydroxybutyrate, phenanthrene, naphthalene, benzoic acid and phenyl acetate as the sole carbon source for growth on TP-agar medium plates.

**Conclusions:** LMJ has 99.14% sequence identity with the *Acidovorax* sp. strain A16OP12 whose genome has not been sequenced yet. LMJ’s ability to use chemicals that are common environmental pollutants makes it a promising candidate for further investigation for its use in bioremediation and, provides us with an incentive to sequence its genome.

## Introduction

Our research laboratory primarily focuses on the functional genomics of eukaryotic oxygenic photosynthesis using the model green micro-alga, *Chlamydomonas reinhardtii*. The work in this article stemmed from a side research project of four undergraduates from the University of West Georgia and, one high school student from the Carrollton High School in Georgia. This undergraduate/high school student research project was centered on minimizing bacterial contamination of *C. reinhardtii* in our research lab.

*Chlamydomonas* can be grown at the lab heterotrophically or photo-heterotrophically at room temperature in Tris-Phosphate-Acetate (TAP) medium, which contains acetate as the sole carbon source ^1^. When grown in TAP medium, *Chlamydomonas* can utilize the exogenous acetate to synthesize glucose using the glyoxylate cycle, without being dependent on photosynthesis for glucose biosynthesis ^2^. Aerobic bacteria can also utilize glyoxylate cycle to metabolize fatty-acids or two carbon-compounds such as acetate to synthesize oxaloacetate, which can be used for gluconeogenesis ^3, 4^. The acetate in the TAP medium can be substituted with alternative carbon sources to test if bacteria can use the tested chemicals as the sole alternative carbon and energy source.

We isolated a bacterial strain from a contaminated *Chlamydomonas* TAP-agar medium culture plate. This bacterium was named LMJ, after the abbreviated name of the *Chlamydomonas* strain it contaminated: LMJ.SG0182. We performed growth analyses and several microbiological tests on LMJ. LMJ grows faster on TAP medium than on lysogeny broth (LB) medium, is a mesophilic, non-enteric, gram-negative bacillus. LMJ does not grow on Mannitol Salt Agar (MSA) and MacConkey agar (MAC) and is sensitive to 1% NaCl. LMJ is starch hydrolysis-negative, gamma hemolytic and is oxidase-positive.

LMJ has a light pinkish-brown color on LB agar and creamy white color on TAP agar. We have found that on TAP + 1% tryptone medium plates in light, LMG colonies are pinkish brown pigmented, unlike that on the TAP-agar medium plates, indicating tryptone ingredients give the pink-brown color to LMJ on TAP+1% tryptone medium. Preliminary spectrophotometric analyses of the pigment/chemicals exuded by LMJ indicate that the light pink-brown pigment is pyomelanin. Pyomelanin is an auto-oxidized and self-polymerized product of homogentisate (HGA), which is an important intermediate in the tyrosine catabolic pathway ^5^. Pyomelanin is one of the many forms of melanin that is produced by bacteria, fungus and other organisms ^5– 10^. Generation of pyomelanin can provide a survival advantage, scavenge free radicals, bind various drugs, offer protection against light and reactive oxygen species, and is involved in iron reduction and acquisition, and extracellular electron transfer ^11– 17^.

We have amplified and sequenced partially LMJ’s 16S rRNA gene. The best hit identified via the NCBI-BLAST analyses of the partial 16S rRNA gene sequence of LMJ is that of an uncultured bacterium clone LIB091_C05_1243 (Accession #: JX086489.1). We have submitted the partial 16S rRNA sequence of LMJ to the GenBank with the definition: Bacterium strain clone LIB091_C05_1243 variant 16S ribosomal RNA gene, partial sequence (Accession number: MN633292.1). The nearest relative of LMJ with a genus name is *Acidovorax* sp. strain A16OP12 (Accession #: MN519578.1).

Betaproteobacteria are highly metabolically diverse and contain chemolithoautotrophs, photoautotrophs, generalist heterotrophs and opportunistic pathogens. *Acidovorax* is a genus of betaproteobacteria of the order Burkholderiales and family Comamonadaceae ^18, 19^. Strains classified as *Acidovorax* have been associated with soils, wastewater treatment plants, plants, and clinical samples ^20– 28^. Reflecting the diversity inherent in this widespread distribution, isolates within this diverse genus have been linked to a variety of phenotypes ranging from plant pathogenicity, to denitrification, to the biodegradation of contaminants ^29^.

*Acidovorax* sp. can degrade alkanes, polyhydroxyalkanoates (PHA) and polycyclic aromatic hydrocarbons (PAH) ^30– 38^. We tested the growth of LMJ on Tris-Phosphate (TP)-agar medium containing PAH, PHA, other aromatic compounds, cyclic alkanes and 10W30 car motor oil to determine if LMJ can use these compounds as the sole carbon source. LMJ was able to use cyclohexyl chloride, phenanthrene, naphthalene, benzoate, phenyl acetate and fresh and combusted 10W30 car motor oil. The abovementioned chemicals occur as common environmental pollutants in soil and water. In future, whole genome sequencing of LMJ and proper quantitative assays to determine LMJ’s ability to degrade (and remove) toxic chemicals from the TP-growth medium are needed to probe its full potential for usage in environmental bioremediation. In this primarily undergraduate and high school student-driven research project, we are presenting our research on the isolation and physiological and biochemical characterizations of the novel bacterial strain, LMJ.

## Methods

### Growth media and cultures

We used the *Chlamydomonas* wild type strain 4A+ (CC- 4051 4A+ mt+) strain in our antibiotic-sensitivity testing experiments. 4A+ strain was maintained on TAP agar medium in dim light intensities (15-20 µmol m ^-2^s ^-1^) at 22°C. Liquid 4A+ cultures were grown in 50 mL flasks in 15 mL of TAP in low light (50-80 µmol m ^-2^s ^-1^) for 3 days. LMJ bacterial strain was maintained in the lab under dim light at 22°C on either TAP or LB agar medium plates. Liquid cultures of LMJ were grown in culture tubes in 3 mL of TAP or LB medium. 4A+ and LMJ liquid cultures were shaken at 150 rpm for aeration on a MaxQ420HP incubator shaker (Thermo Fisher Scientific, Waltham, MA). Light intensities were measured using a LI-250A light meter (LI-COR, Inc., Lincoln, NE).

The detailed protocol utilized to produce the TAP and TP medium used in this article is available from Protocols.io: https://doi.org/10.17504/protocols.io.bgzujx6w.

### LMJ growth analyses

*a) Testing salt-sensitivity of LMJ:* LMJ was streaked on LB, TAP, LB-1% NaCl and TAP +1% NaCl agar medium plates. Streaked LMJ media plates were incubated either 22°C or at 37ºC for 3 days and then imaged.

*b) Testing bile salts-/salt-sensitivity and ability to ferment lactose/mannitol:* Growth assays on MAC and MSA were performed by streaking LMJ on MAC and MSA plates purchased from Carolina Biological (Burlington, NC). Media plates were incubated at room temperature for 4 days and then imaged to capture the LMJ growth and the pH change in the media. *Escherichia coli* and *Staphylococcus aureus* was used as a positive control for the MAC and the MSA experiment, respectively.

*c) Testing LMJ’s ability to use different sugars as a sole carbon source:* LMJ was streaked on agar plates containing TP-phenol red medium (pH 7.2) supplemented with 1% glucose, 1% sucrose and 1% lactose, respectively. Media plates were imaged after 5 days of growth at 22°C to capture the LMJ growth and the pH change in the media.

*d) Testing LMJ’s ability to use different hydrocarbons and aromatic compounds as a sole carbon source:* TP-agar plates were coated with different doses of cyclohexyl chloride, phenanthrene, naphthalene, benzoic acid, phenyl acetate and fresh and combusted 10W30 oil, using a modified technique ^39^. 1% stock solutions of all chemicals (except 10W30 motor oil) were prepared by either dissolving or diluting the chemicals in chloroform. 10W30 fresh and used motor oils were diluted in chloroform to make a 2% (v/v) stock solution. 4 mL of 1% cyclohexyl chloride and 1% polyhydroxybutyrate (PHB) were coated on a TP plate. 2 mL and 4 mL of 1% phenanthrene stock solution were coated on TP plates. 0.5 mL, 1 mL and 2 mL of 1% napthalene, 1% benzoate and 1% phenyl acetate and 2% combusted and fresh 10W30 car motor oil were coated on TP plates. LMJ was streaked on the chemical-coated TP plates and incubated at room temperature. After 2 weeks, the plates were imaged using a Samsung Galaxy S5 camera. Images of all medium plates are available as *Underlying data* ^40^.

### Gram staining, oxidase test and starch hydrolysis test

*a) Gram staining*: LMJ from LB-agar plate and LB and TAP shaking liquid cultures were used for Gram staining. Gram staining was performed using standard Gram staining protocol using Gram stain reagents from VWR (Radnor, PA). Gram-stained cells were viewed under oil immersion lens with a X100 magnification. Images of gram-stained cells were taken by a Samsung Galaxy S5 camera using a cell phone adapter for the microscope eyepiece.

*b) Oxidase test:* Oxidase test was performed using Difco DrySlide Oxidase Disposable Slide purchased from Carolina Biological (Burlington, NC). The dry slide contains a film coated with an oxidase reagent (tetramethyl-p-phenylenediamine dihydrochloride). LMJ and *Microbacterium* sp. cells were taken from tryptic soy agar medium plates which does not contain any fermentable sugar. Fermentable sugars generate acid which can drop the pH below 5.1 and can give a false negative oxidase test result ( https://microbeonline.com/oxidase-test-principle-procedure-and-oxidase-positive-organisms/) (Carolina Biological, Burlington, NC). Cells were streaked on the dry slide in two separate reaction areas. After 10 seconds, the color of the streak was captured with a Samsung Galaxy S5 camera.

*c) Starch hydrolysis test*: Starch hydrolysis test was performed on Mueller-Hinton-agar medium plates purchased from Carolina Biological (Burlington, NC). LMJ and *E. coli* were streaked on Mueller-Hinton agar medium plates and incubated for 48 hours at 37°C. After 48 hours of growth, plates were imaged. After imaging, Mueller-Hinton agar plates were flooded with Gram iodine and incubated for 10 minutes to test for the absence or presence of starch in the medium and the plate was re-imaged.

### Antibiotic susceptibility test using disc diffusion method

We modified the Kirby-Bauer (KB) disc diffusion method to perform the antibiotic susceptibility tests on TAP-agar plates ( https://www.asm.org/Protocols/Kirby-Bauer-Disk-Diffusion-Susceptibility-Test-Pro). We grew LMJ in 3 mL of liquid TAP or in liquid LB and diluted the overnight culture 5-fold with liquid medium. The optical density of the 5-fold diluted culture was measured at 600 nm with a Beckman Coulter DU 730 Life science UV/Vis spectrophotometer (Brea, CA) ^41^. The optical density of culture was adjusted with liquid medium to achieve an optical density of 0.08–13 which matches the 0.5 McFarland standard. *Chlamydomonas* strain 4A+ was grown in liquid TAP for three days and the optical density of the *Chlamydomonas* culture was measured at 750 nm with a Beckman Coulter DU 730 Life science UV/Vis spectrophotometer (Brea, CA) ^42^. Measurement at 750 nm avoids the absorption of light by cellular pigments (chlorophyll and carotenoids) and is treated as a pure light scattering measurement ^42^. The optical density of the *Chlamydomonas* culture was adjusted with TAP medium to achieve an optical density of 0.3–0.4.

Using a sterile swab, optical density-adjusted cultures of LMJ and *Chlamydomonas* were plated on the TAP plates. Two sterile filter paper discs were placed on each TAP plate with a separating distance of 4–5 cm. On one disc 50 µg or 100 µg of the antibiotic in a 20 µL total volume and on the other disc, 20 µL of sterile water was added (control). Antibiotics that were applied were: penicillin; neomycin, chloramphenicol and polymyxin B. Antibiotic plates were incubated at room temperature for 3–4 days. LMJ and *Chlamydomonas* plates were imaged after 3 and 4 days of incubation respectively, as *Chlamydomonas* grows slowly than bacteria. Diameters of zone of inhibitions were measured using a ruler. Standard deviations and means were calculated using Excel and, statistical analyses of the data from three biological replicates per experiment were performed using Microsoft Excels’ t-Test: Paired Two Sample for Means tool in the analysis ToolPak. Both One-Tailed and Two-Tailed Hypothesis Tests were performed. Statistical analyses ^43^ and images of all antibiotic plates ^44^ are available as part of the *Underlying data*.

### Analyses of pigment/chemicals of LMJ

LMJ from TAP-agar plates were streaked on fresh TAP +1% tryptone-agar plates. One plate was incubated under light intensity of 350 µmol photons m ^-2^s ^-1^ and the other plate was kept in dark at 22ºC. Images of the plates were taken after 3 days of growth to monitor pigment production. LMJ cells were treated in three different ways for spectrophotometric analyses. 1) LMJ was grown in 2.5 mL of LB medium at 37°C for 3 days. 2 mL of the LB culture was centrifuged at 5000 X g for 2 minutes. The supernatant and the cell pellet were collected. Supernatant was alkalinized (20 µL of 6 M NaOH per ml of sample) and centrifuged at 16,000 X g for 2 minutes and the supernatant was collected. 2) LMJ cell pellet from treatment 1 was resuspended in 200 µL of 1% NaNO _2_ solution and 1mL of 0.1N-HC1 by vortexing the sample for few minutes and incubated for 5 minutes. 3) LMJ cells from a LB-agar medium plate was resuspended in 1 mL of 6M NaOH by vortexing and incubated for 5 minutes. Acidified and alkalinized tubes of cell pellet washes from treatments 2 and 3 were centrifuged at 16,000 X g for 2 minutes and the supernatants were collected separately.

Supernatants obtained from the above stated three treatments were filtered using a 5 mL syringe fitted to a nylon membrane filters with a cut-off of 0.45 µm. Pigment formation in the alkalinized supernatant from the liquid LB culture and, the alkalinized- and acidified- supernatants from cell pellet washes were analyzed by using the wavelength scan program ranging 200 - 600 nm in a Beckman Coulter DU 730 Life science UV/Vis spectrophotometer (Brea, CA). To detect pyomelanin in the supernatant of the LB liquid culture, the absorption peak at the 400 - 405 nm region was monitored. To detect homogentisate (HGA) in the alkalinized supernatant from the cell pellet wash, the absorption peak at the 290 nm was monitored. To detect 1:4-benzoquinone-2-acetic acid (benzoquinone acetate; BQA), the absorption peak at the 250 nm was monitored in the acidified supernatant obtained from the cell pellet wash. Our protocol is a “quick and crude” version of the protocol available in the literature for preparation of samples for spectrophotometric assays of pyomelanin, BQA and HGA ^45– 47^.

### Genomic DNA isolation and PCR amplification of the partial 16S rRNA gene

Genomic DNA was isolated using Qiagen’s blood and cell culture DNA mini kit (Qiagen, Valencia, CA) according to the protocol given in the technical manual. DNA concentration and purity of the isolated genomic DNA sample were measured using a Nanodrop 2000 spectrophotometer (Thermo Fisher Scientific, Waltham, MA). Genomic DNA sample was separated on a 1% agarose gel and the quality of the sample was visualized by imaging the gel with a BioRad Molecular Imager Gel Doc XR+ (BioRad, Hercules, CA).

Forward and reverse 16S rRNA PCR primers were designed based on sequences in the literature for selecting primer pairs with the best overall coverage and phylum spectrum to reduce the bias in PCR-based microbial diversity studies ^48^. The sequence of the forward primer 16SF is: 5’ CCTACGGGNGGCWGCAG 3’ and that of the reverse primer 16SR is: 5’ GACTACHVGGGTATCTAATCC 3’. HotStar Taq Plus DNA polymerase enzyme kit (Qiagen, Valencia, CA) was used for PCR following the given cycling conditions given in the Qiagen protocol booklet.

### Gel extraction of the 16S rRNA amplicon and its cloning

The PCR-amplified partial 16S rRNA genomic product was excised from the agarose gel and purified using the QIAquick Gel Extraction Kit (Qiagen, Valencia, CA). The purified PCR product was cloned using the TOPO TA cloning kit in the pCR4-TOPO TA vector (Thermo Fisher Scientific, Waltham, MA) according to the protocol given in the technical manual. One clone harboring the partial 16S rRNA gene of LMJ was sequenced by the UC Berkeley DNA Sequencing Facility. Chromas Lite and BLAST program were used to analyze DNA sequences. Raw electropherogram files and sequence text files are available as *Underlying data* ^49^.

## Results

### Isolation of the bacterial strain LMJ

**Figure 1.**
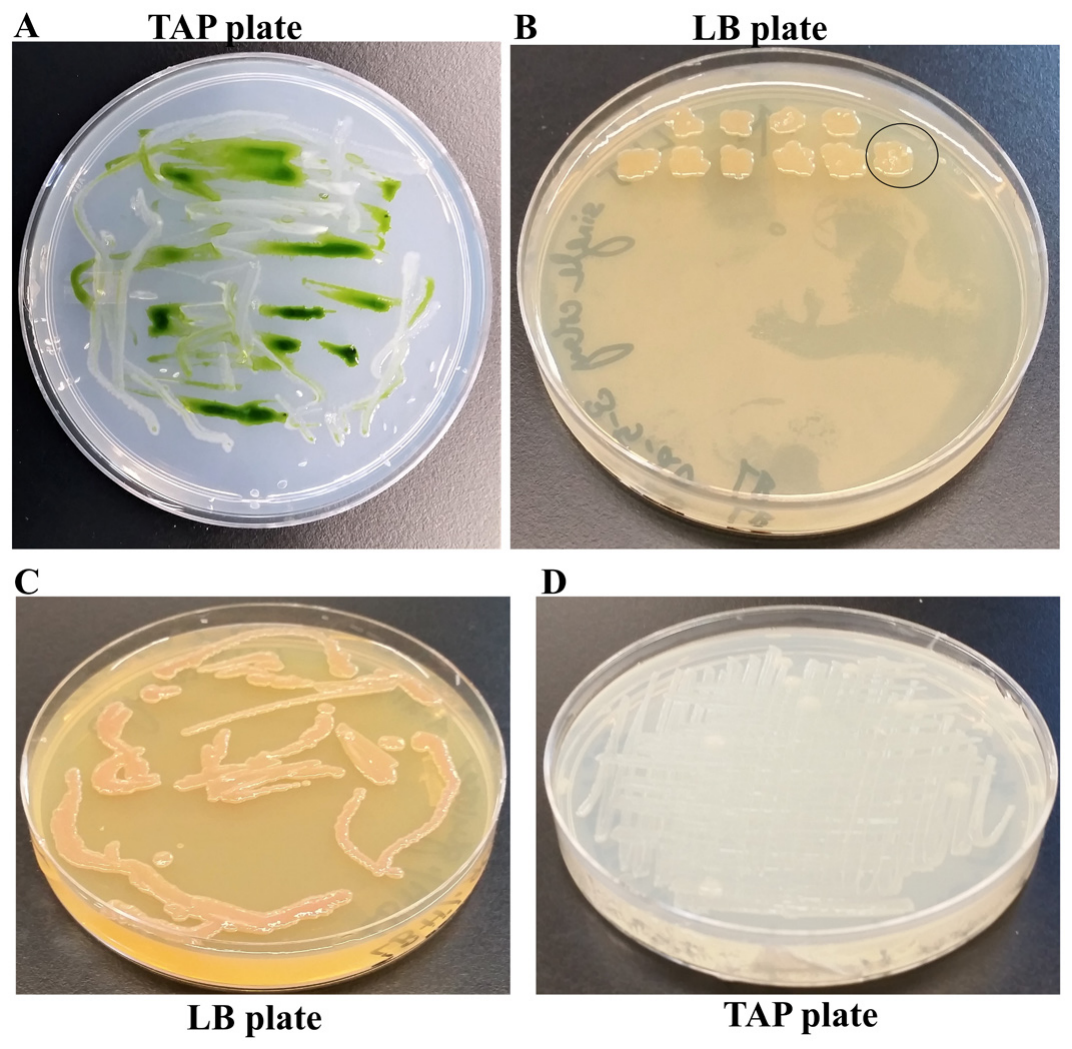
Isolation of a novel bacterial strain from a contaminated *Chlamydomonas* Tris-Acetate-Phosphate (TAP)-agar medium plate. ( **A**) TAP-agar medium plate showing bacterial contamination of a *Chlamydomonas* strain, LMJ.SG0182 at room temperature (22ºC). The bacterium is named LMJ, after the abbreviated *Chlamydomonas* LMJ.SG0182 strain. ( **B**) Ten single colonies of LMJ on a lysogeny broth (LB)-agar medium plate. Colony # 10 outlined by the black circle was picked for culture stock maintenance and further analyses. ( **C**) LB-agar medium plate of purified LMJ strain ( **D**) TAP-agar medium plate of purified LMJ strain. Culture plates shown in ( **C**) and ( **D**) were imaged after 5 days of growth at 37ºC.

We found a creamy white bacterial contamination on a *Chlamydomonas* strain, LMJ.SG0182 TAP plate at our laboratory. ( Figure 1A). We purified the bacterial strain using the streak plate method to isolate single bacterial colonies on fresh TAP medium plates. We picked ten single colonies and transferred them to a LB agar medium plate ( Figure 1B). Colony # 10 was selected for our studies ( Figure 1B). Colony # 10 was streaked on fresh LB and TAP-agar medium plates to maintain the purified bacterial strain in the lab ( Figure 1C, D). We named this bacterial strain as LMJ after the abbreviated name of the *Chlamydomonas* strain LMJ.SG0182, as it was isolated from this *Chlamydomonas* strain’s culture plate. LMJ is an acid-devouring strain as it was able to grow on the TAP-agar medium ( Figure 1D). LMJ culture on LB agar medium appears pigmented with a light pinkish-brown color ( Figure 1C) but on TAP agar medium the culture is creamy white ( Figure 1D).

Antibiotic sensitivity data for the bacterial strain LMJ (Bacterium strain clone LIB091_C05_1243 variant 16S ribosomal RNA; GenBank Accession # MN633292.1). The Excel data file, Data S1 LMJ.xlsx contain information about the means and standard deviations of the zones of inhibitions of the bacterial strain LMJ and *Chlamydomonas*, induced by 4 antibiotics. The Excel data file, Data S2 LMJ.xlsx contain statistical analyses of the zones of inhibitions of the bacterial strain LMJ and *Chlamydomonas*, induced by 4 antibiotics. Antibiotics used are: Penicillin, Chloramphenicol, Neomycin & Polymyxin B. Three biological replicates were used for the generation of the data. Two different doses of antibiotics were used: 50 and 100 micrograms of each antibiotic. Click here for additional data file. Copyright: © 2020 Mitra M et al. 2020

### TAP medium can be used as a new stringent minimal medium for LMJ

TAP medium recipe can be found at the website of Chlamydomonas Resource Center. Hutner’s trace element solution is an ingredient in the TAP medium. Hutner’s trace element recipe can be found at https://www.chlamycollection.org/methods/media-recipes/hutners-trace-elements/. M9 medium is the standard minimal medium for growing bacteria. In our lab we use a slightly modified TAP medium recipe which has a final concentration of phosphate, nitrogen, magnesium and calcium approximately 10-fold, 2-fold, 2-fold and 2-fold higher than that in the TAP recipe found on the Chlamydomonas Resource Center website, respectively. Final concentrations of acetate and Hutner’s trace elements are the same in both TAP recipes. Table 1 compares the chemical ingredients in our lab’s TAP recipe with that present in the standard M9 medium described at http://www.thelabrat.com/protocols/m9minimal.shtml. M9 medium has a final concentration of phosphate, nitrogen, magnesium and carbon in the medium approximately 70-fold, 2.5-fold, 4-fold and 5-fold higher than that present in our lab’s TAP medium, respectively. Additionally, M9 contains 0.05% salt (8.56 mM) ( Table 1). TAP has additional trace elements like iron, zinc, copper, manganese, cobalt, boron and molybdenum which are components of the Hutner’s trace element solution ( Table 1). We have substituted the acetate in the TAP medium with alternative carbon sources to perform various experiments that are described below in the result section.

**Table 1.** Comparison of chemical ingredients in the TAP and in M9 growth media. TAP medium is used for growing *Chlamydomonas* heterotrophically or photo-heterotrophically. M9 medium is the standard minimal medium for growing bacteria. **Note:** Hutner’s trace element is an ingredient in the TAP medium. TAP trace elements and EDTA shown in the table are components in the Hutner’s trace element solution. pH of Hutner’s solution is adjusted to 6.5 using KOH (not NaOH) pellets before it is used to make the TAP medium. Both growth media have (pH 7-7.2). Acetate in the TAP medium can be substituted with alternative carbon sources. TAP medium minus acetate is the TP medium in our work and the media recipes can be found at https://doi.org/10.17504/protocols.io.bgzujx6w.

Chemical	Final concentration in 1L of TAP medium	Final concentration in 1L of M9 minimal medium
Na _2_HPO _4_-7H _2_O	-	47.74 mM; M9 salt
KH _2_PO _4_	0.396 mM; phosphate solution	22.04 mM; M9 salt
K _2_HPO _4_	0.6 mM; phosphate solution	-
NaCl	-	8.56 mM; M9 salt
NH _4_Cl	7.48 mM; TAP salt	18.69 mM; M9 salt
MgSO _4_.7H _2_O	0.405 mM; TAP salt	2 mM
Carbon source	0.1% glacial acetic acid (99.7%)	0.4 % (glucose or any other carbon source)
CaCl _2_ .2H _2_O	0.4525 mM; TAP salt	0.1mM
Tris base	19.97 mM	
FeSO _4_.7H _2_O	0.018 mM; trace element	-
ZnSO _4_.7 H _2_O	0.0765mM; trace element	-
H _3_BO _3_	0. 184 mM; trace element	-
MnCl _2_.4H _2_O	0.0256 mM; trace element	-
CuSO _4_.5H _2_O	0.0063 mM; trace element	-
(NH _4_) _6_Mo _7_O _24_.4 H _2_O	0.00089 mM; trace element	-
CoCl _2_.6H _2_O	0.00068 mM; trace element	-
EDTA	134 mM	

### Gram staining

**Figure 2.**
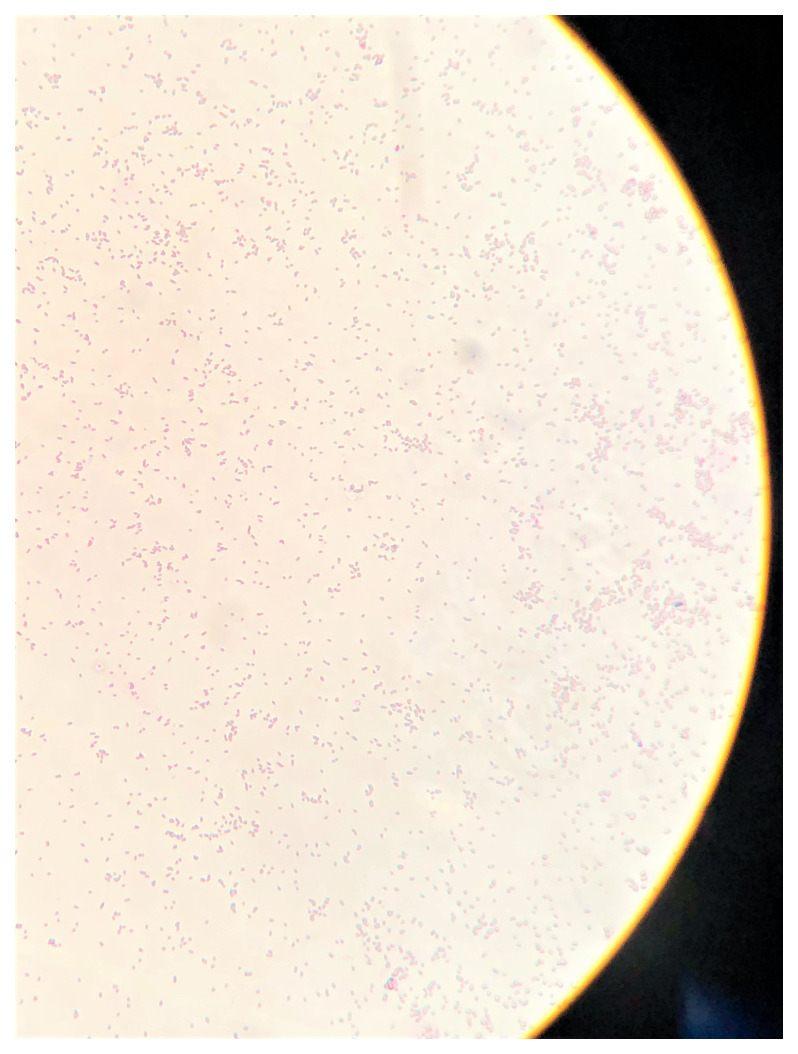
Gram-stained LMJ imaged under 100X magnification. LMJ cells from a LB-agar medium plate was used for gram-staining. Gram-stained cells were visualized and imaged under an oil immersion lens of a bright-field microscope.

Gram staining of LMJ cells from a LB-agar medium plate revealed that LMJ cells are straight to slightly curved rods ( Figure 2). Cells occur singly, in pairs or in short chains. LMJ is a gram-negative bacterium ( Figure 2).

Images of antibiotic plates of the bacterial strain LMJ (Bacterium strain clone LIB091_C05_1243 variant 16S ribosomal RNA; GenBank Accession # MN633292.1) and green micro-alga Chlamydomonas from the antibiotic susceptibility disc diffusion tests. This file contains 16 images of antibiotic plates used for the antibiotic susceptibility tests using the disc diffusion method for Chlamydomonas and the bacterial strain, LMJ. Antibiotics tested are: penicillin, chloramphenicol, polymyxin B and neomycin. Two different doses of antibiotics were used: 50 and 100 micrograms of each antibiotics. **On the LMJ antibiotic plates, the filter paper disc on the right contains the antibiotic and that on the left contains sterile water (control). On the *Chlamydomonas* antibiotic plates, the filter paper disc on the left contains the antibiotic and that on the right contains sterile water (control).**
Copyright: © 2020 Mitra M et al. 2020

### Analyses of LMJ growth on TAP- and LB-agar medium plates at different temperatures

**Figure 3.**
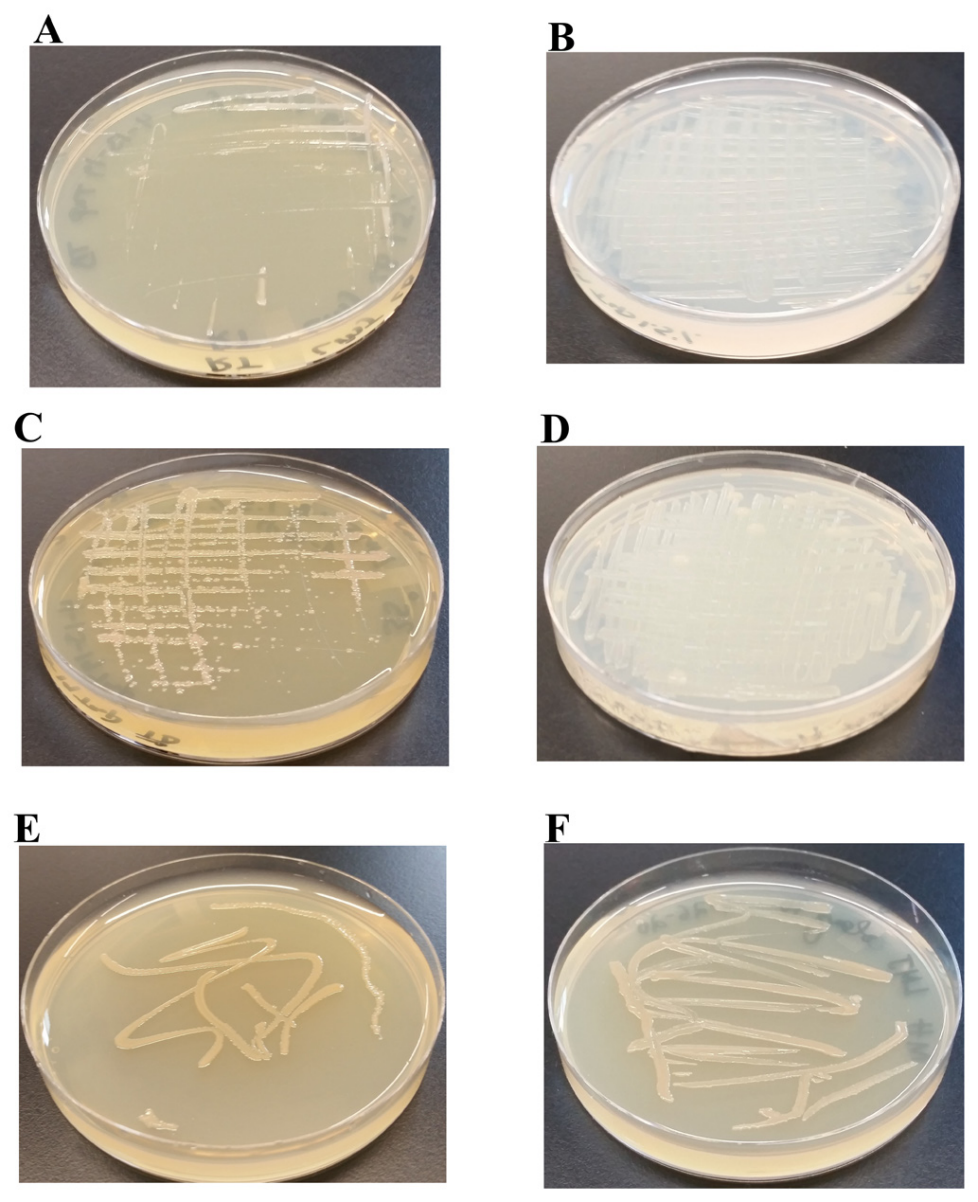
LMJ growth on TAP- and LB-agar medium plates at different temperatures. ( **A**) Growth on LB-agar medium plate at room temperature (22ºC). ( **B**) Growth on TAP-agar medium plate at room temperature. ( **C**) Growth on LB-agar medium plate at 37ºC. ( **D**) Growth on TAP-agar medium plate at 37ºC. ( **E**) LMJ growth on Mueller-Hinton agar medium plate at 22ºC. ( **F**) LMJ growth on Mueller-Hinton agar medium plate at 37ºC. Culture plates were imaged after 5 days of growth.

We have monitored the growth of LMJ on LB-agar, TAP-agar and Mueller-Hinton agar medium over 5 days. LMJ has less dense growth on LB agar than on TAP agar or on Mueller-Hinton agar at 22°C ( Figure 3A, B, E). LMJ has dense growth at 37°C than at 22°C on all three media tested ( Figure 3C, D, F). It is to be noted that LMJ appears lightly pigmented on both LB-agar and Mueller-Hinton agar but not on TAP-agar ( Figure 1C and Figure 3C, E, F).

Tests using Tris-Phosphate medium (TP) to see if hydrocarbons, aromatic compounds and polyhydroxyalkanoates can be used by the bacterium LMJ (Bacterium strain clone LIB091_C05_1243 variant 16S ribosomal RNA; GenBank Accession # MN633292.1) as the sole carbon source. This file contains 23 images of TP (Tris-Phosphate) medium plates containing different alternative carbon sources. Bacterium LMJ was streaked on these chemical plates to test if LMJ can utilize these chemicals as the sole carbon source for energy and growth. 1% stocks of the following chemicals were tested: cyclohexyl chloride, phenanthrene, napthalene, benzoic acid, phenyl acetate. 2% (v/v) stocks of fresh and used car motor oil 10W30 were also tested. The doses used are given in mL in the file name. Click here for additional data file. Copyright: © 2020 Mitra M et al. 2020

### Salt-sensitivity of LMJ

**Figure 4.**
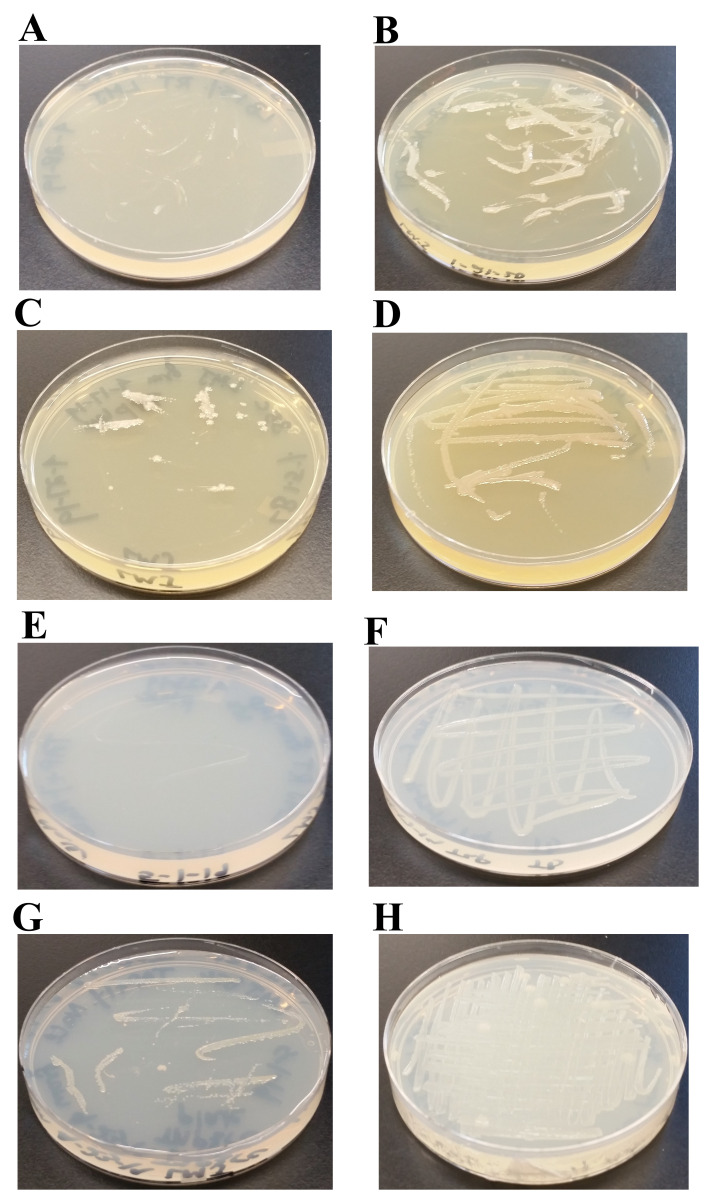
Effect of 1% NaCl on LMJ growth at different temperatures. ( **A**) LMJ growth on LB (contains 1% NaCl)-agar medium plate at 22ºC. ( **B**) LMJ growth on LB (minus 1% NaCl)-agar medium plate at 22ºC. ( **C**) LMJ growth on LB (contains 1% NaCl)-agar medium plate at 37ºC. ( **D**) LMJ growth on LB (minus 1% NaCl)-agar medium plate at 37ºC. ( **E**) LMJ growth on TAP+1% NaCl-agar medium plate at 22ºC. ( **F**) LMJ growth on TAP-agar medium plate at 22ºC. ( **G**) LMJ growth on TAP+1% NaCl-agar medium plate at 37ºC. ( **H**) LMJ growth on TAP-agar medium plate at 37ºC. Culture plates were imaged after 3 days of growth.

LMJ grows on the nutrient-rich LB agar medium slowly compared to that on the TAP agar medium ( Figure 3). TAP does not contain NaCl ( Table 1) but LB medium has 1% NaCl ( http://2018.igem.org/wiki/images/f/f2/T--Toronto--_LB_Medium_Preperation_Protocol.pdf). Gram-negative bacteria are usually sensitive to salt. Hence, we studied the growth of LMJ on LB, LB -1% NaCl, TAP, and TAP +1% NaCl-agar medium at 22°C and at 37°C. LMJ grew faster on LB-1% NaCl agar compared to that on the LB agar at both temperatures ( Figure 4A-D). LMJ grew very slowly on TAP +1% NaCl agar compared to that on the TAP-agar at both temperatures ( Figure 4E-H). The slow growth of LMJ was very pronounced at 22°C than at 37°C on both LB-agar and TAP +1% NaCl-agar medium ( Figure 4A, C, E, G). Taken together, our results show that LMJ is sensitive to even 1% of NaCl in LB that is tolerated well by many Gram-negative bacteria.

16S rRNA partial gene sequences of the bacterial strain LMJ (Bacterium strain clone LIB091_C05_1243 variant 16S ribosomal RNA; GenBank Accession # MN633292.1) Dataset contains 2 abi extension files obtained from sequencing of the LMJ strain's 16S rRNA gene partially. There are 2 electropherogram files and corresponding text files of DNA sequences in the dataset. The 16S rRNA gene was partially amplified from LMJ and cloned in the TOPO-TA vector (Thermo Scientific Fisher) for DNA sequencing. Sanger dideoxy sequencing technology was used for DNA sequencing. Click here for additional data file. Copyright: © 2020 Mitra M et al. 2020

### Liquid medium grown-LMJ cells are prone to cell lysis during Gram staining

**Figure 5.**
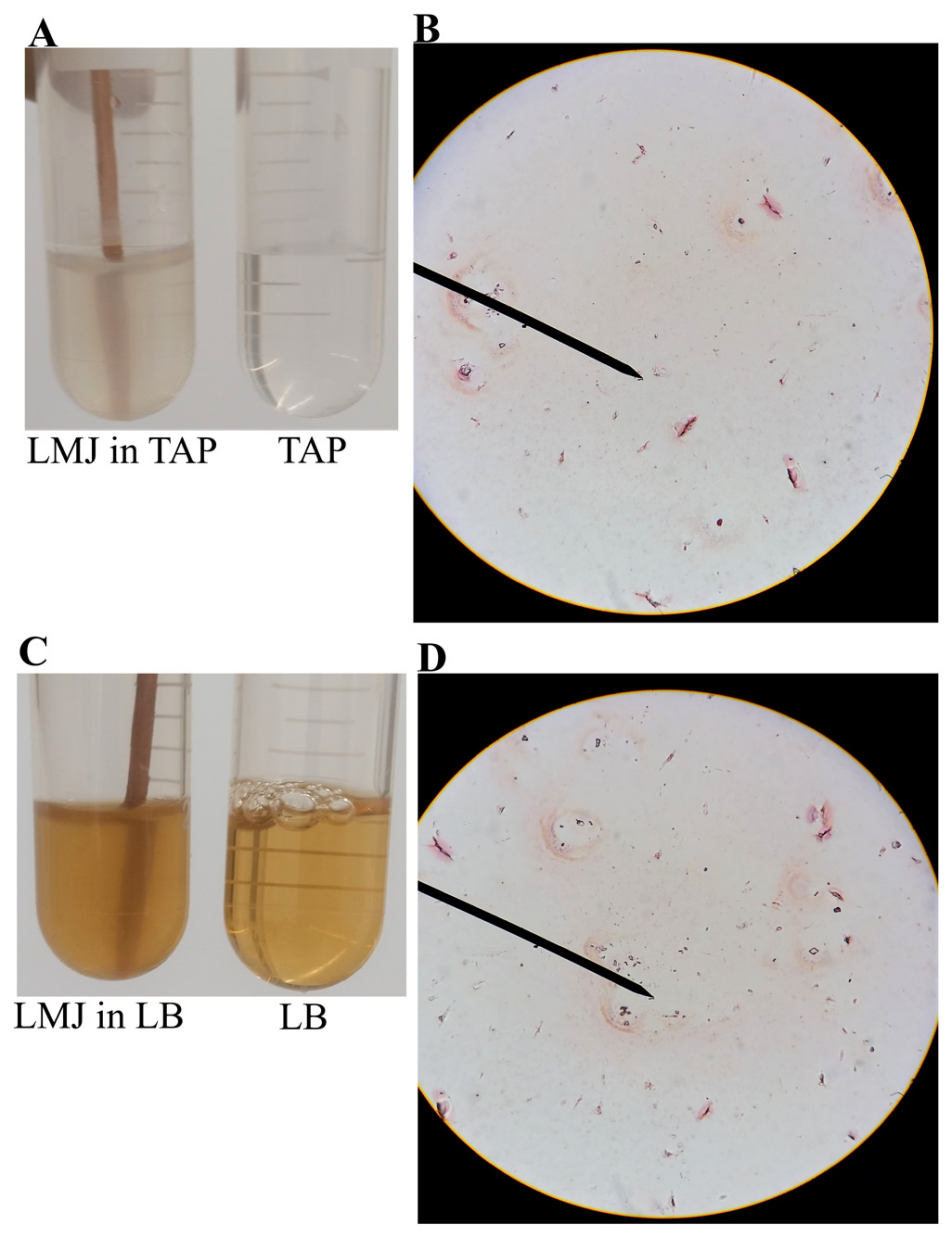
Liquid medium grown-LMJ cells are prone to cell lysis during Gram staining. ( **A**) A culture of LMJ grown for 24 h in liquid TAP and liquid TAP medium (control). ( **B**) Gram-stained LMJ cells from the 24 hours-TAP liquid culture. ( **C**) A 24 hours-grown culture of LMJ in liquid LB and liquid LB medium (control). ( **D**) Gram-stained LMJ cells from the 24 hours-grown LMJ LB liquid culture. Mostly membrane debris, few rods and round shaped cells can be seen. Original gram-stained files can be found at 10.6084/m9.figshare.12420893.

In Figure 2, LMJ cells from a LB-agar medium plate was used for Gram staining. We performed Gram staining using cells from LMJ liquid TAP and liquid LB cultures that were shaking in an incubator shaker at 150 rpm at 37°C ( Figure 5). Although the turbidity of the TAP or the LB liquid culture indicates dense growth ( Figure 5A, C), Gram stain of LMJ liquid cultures revealed mainly cell lysis and membrane debris with very few rods and round intact cells ( Figure 5B, D; *Underlying data* ^50^). We have monitored the LMJ LB and liquid culture growth over a period 96 hours and we did not see any clearing of the TAP and LB liquid cultures (see *Underlying data* ^50^). Cells from the liquid TAP culture were plated on a fresh TAP-agar medium plate and LMJ grew back within 4 days at room temperature (see *Underlying data* ^50^). Cells in liquid culture were not dead although we saw mainly cell debris and very few intact cells after gram staining. Taken together, our results indicate that LMJ is probably a biofilm forming bacteria. It could not form biofilm in a shaking liquid culture and cells were prone to lysis in the shaking liquid culture (see *Discussion*).

Growth of bacterial strain LMJ (Bacterium strain clone LIB091_C05_1243 variant 16S ribosomal RNA; GenBank Accession # MN633292.1) and Staphylococcus aureus on Tryptic Soy Agar medium plates containing 5% sheep blood. This file contains five images. We have included two figures showing the 24 hours-growth on the blood agar media plates, the growth of the bacterial strain LMJ is on the left on the blood agar plate and that of *Staphylococcus aureus* is on the right of the blood agar plate. Images were taken after 1 day of growth at 37C. *S. aureus* is beta-hemolytic while LMJ is gamma hemolytic. We have also included three additional images of LMJ growth on Tryptic Soy Agar medium for 24 hours, 48 hours and 72 hours at 37C to show that even with prolonged incubation at 37C, LMJ does not show complete or partial hemolysis on blood agar medium plates. Tryptic Soy Agar medium plates containing 5% sheep blood were purchased from Carolina Biological (Burlington, NC). Click here for additional data file. Copyright: © 2020 Mitra M et al. 2020

### Pigment studies

Many bacteria are known to produce a brown-black melanin pigment called pyomelanin ^5, 17^. Light and oxygen are needed for the formation of pyomelanin ^45^. Aromatic amino acid tyrosine is needed for pyomelanin formation ^5^. Phenylalanine can also contribute to the synthesis of pyomelanin as it can be can be converted to tyrosine by the enzyme phenylalanine-4-hydroxylase ^51^. LB medium contains 1% tryptone which is a rich source of different amino acids including the aromatic amino acids ( https://khimexpert.com/wp-content/uploads/2018/12/GCM23-Tryptone.pdf).

**Figure 6.**
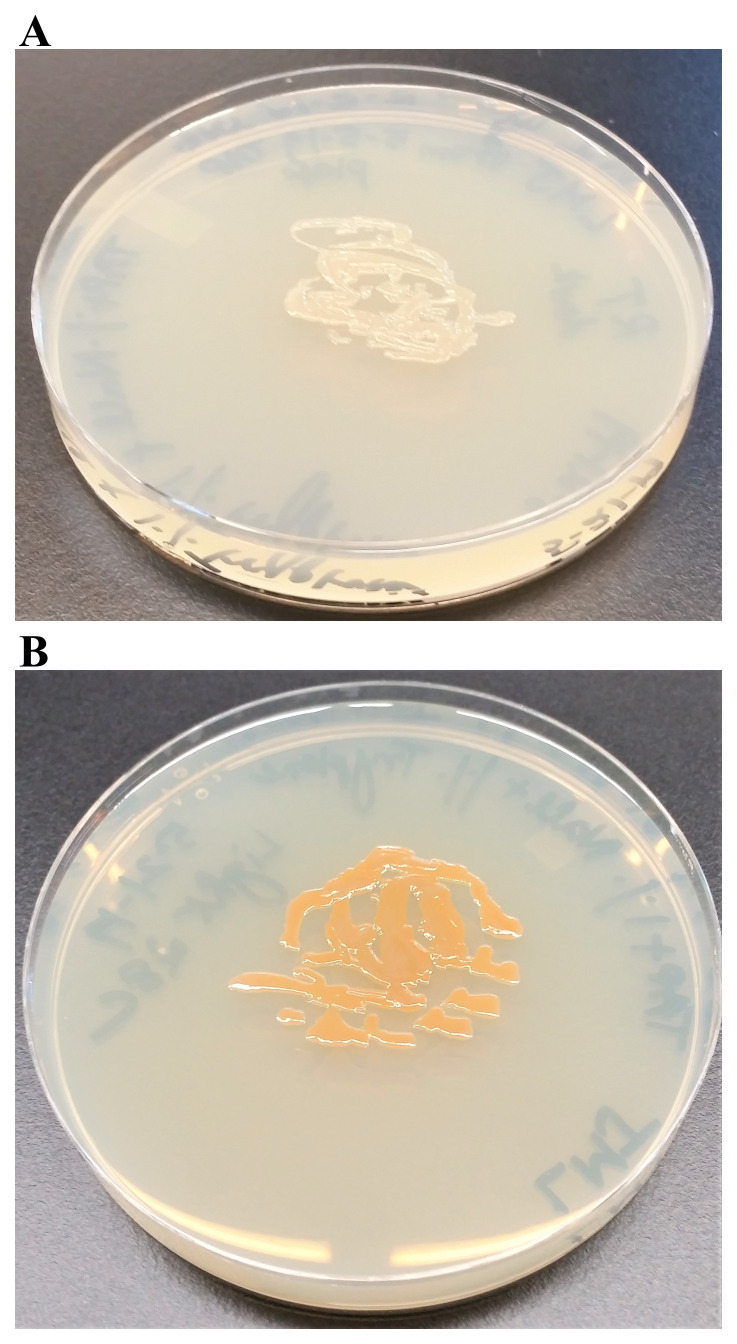
Pigment production in LMJ in dark and in light on TAP + 1% Tryptone-agar. ( **A**) LMJ on TAP +1% tryptone agar at room temperature. ( **B**) LMJ on TAP +1% tryptone-agar under light intensity of 350 µmol photons m ^-2^s ^-1^ at room temperature. Images were taken after 3 days of growth.

We tested if the light brown color of LMJ on LB medium is because of tryptone which provides aromatic amino acids. We grew LMJ on TAP +1% tryptone-agar medium plates in dark and in light. In the dark, on TAP + 1% tryptone medium plate LMJ did not appear to be pigmented, unlike that in the light ( Figure 6). We found that 1% tryptone and light are needed for pigment production in LMJ ( Figure 6).

TAP and LB liquid cultures of the bacterial strain LMJ (Bacterium strain clone LIB091_C05_1243 variant 16S ribosomal RNA; GenBank Accession # MN633292.1) grown for 4 days and Gram stains of LMJ from these liquid cultures. Growth of LMJ on TAP-agar from a 96 hours-grown TAP liquid culture is also shown. This file contains 11 images. Bacterial strain LMJ was grown in liquid TAP and liquid LB medium for 4 days (96 hours) in an incubator shaker, shaking at 150 rpm at 37C. Images were taken every 24 hours to monitor the turbidity/growth of these liquid cultures over 96 hours. Gram stains were performed on the 24 hours-grown liquid TAP and LB cultures. There were cell debris, membrane fragments, some small rods and round shaped cells were visible after gram staining. 96 hours liquid TAP culture was plated on a TAP-agar medium plate. LMJ grew back within 4 days at room temperature. This result shows that although gram stain could not detect many intact cells in the liquid culture, cells were not dead. LMJ could not form a biofilm in shaking cultures properly and this induced cell lysis in liquid cultures. It can form biofilms on TAP-agar when plated from the liquid TAP culture. Click here for additional data file. Copyright: © 2020 Mitra M et al. 2020

**Figure 7.**
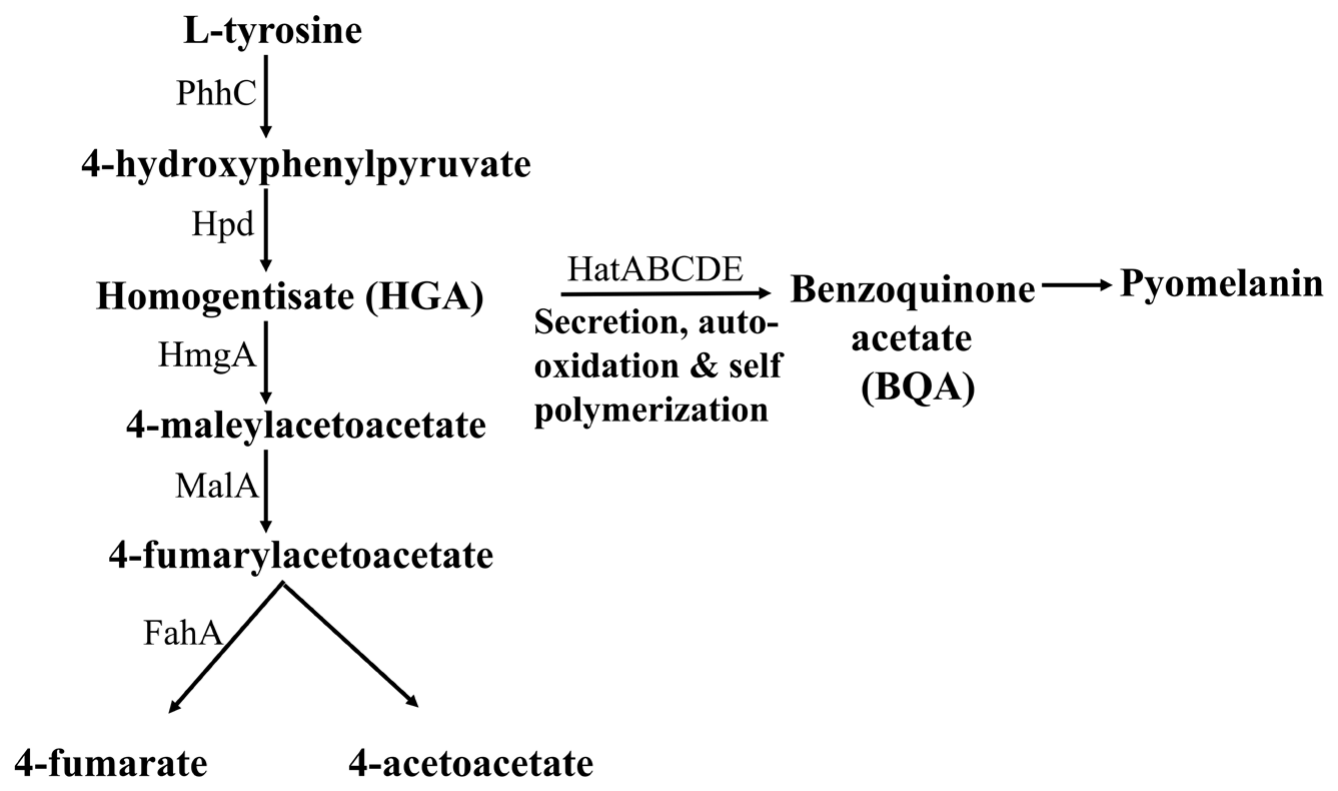
A simplified tyrosine catabolism pathway in *Pseudomonas aeruginosa*. PhhC: Family I aminotransferase; Hpd: 4-hydroxyphenylpyruvate dioxygenase; HmgA: homogentisate 1,2-dioxygenase; MalA: maleylacetoacetate isomerase; FahA: fumarylacetoacetate ** hydrolase; HatABCDE: ABC transporter.

Pyomelanin is a negatively charged extracellular pigment of high molecular weight, derived from the tyrosine catabolism pathway ^11^( Figure 7). Pyomelanin is usually produced because of defects in the tyrosine catabolism pathway that leads to an accumulation of homogentisate (HGA) and occurs naturally in many pyomelanin over-producing strains ^5, 52^ ( Figure 7). Accumulation of HGA can occur because of an inactivation of the enzyme homogentisate 1, 2 dioxygenase (HmgA) ^52, 53^ ( Figure 7). Accumulated HGA is secreted out of the cells by an ABC transporter and is auto-oxidized to 1:4-benzoquinone-2-acetic acid (benzoquinone acetate; BQA) and then polymerized to form the brown pigment pyomelanin ( Figure 7). We wanted to see if we can detect HGA, BQA and pyomelanin in the alkalinized LMJ LB liquid culture medium and in the acidified and alkalinized LMJ cell pellet-wash supernatants ^45– 47^.

**Figure 8.**
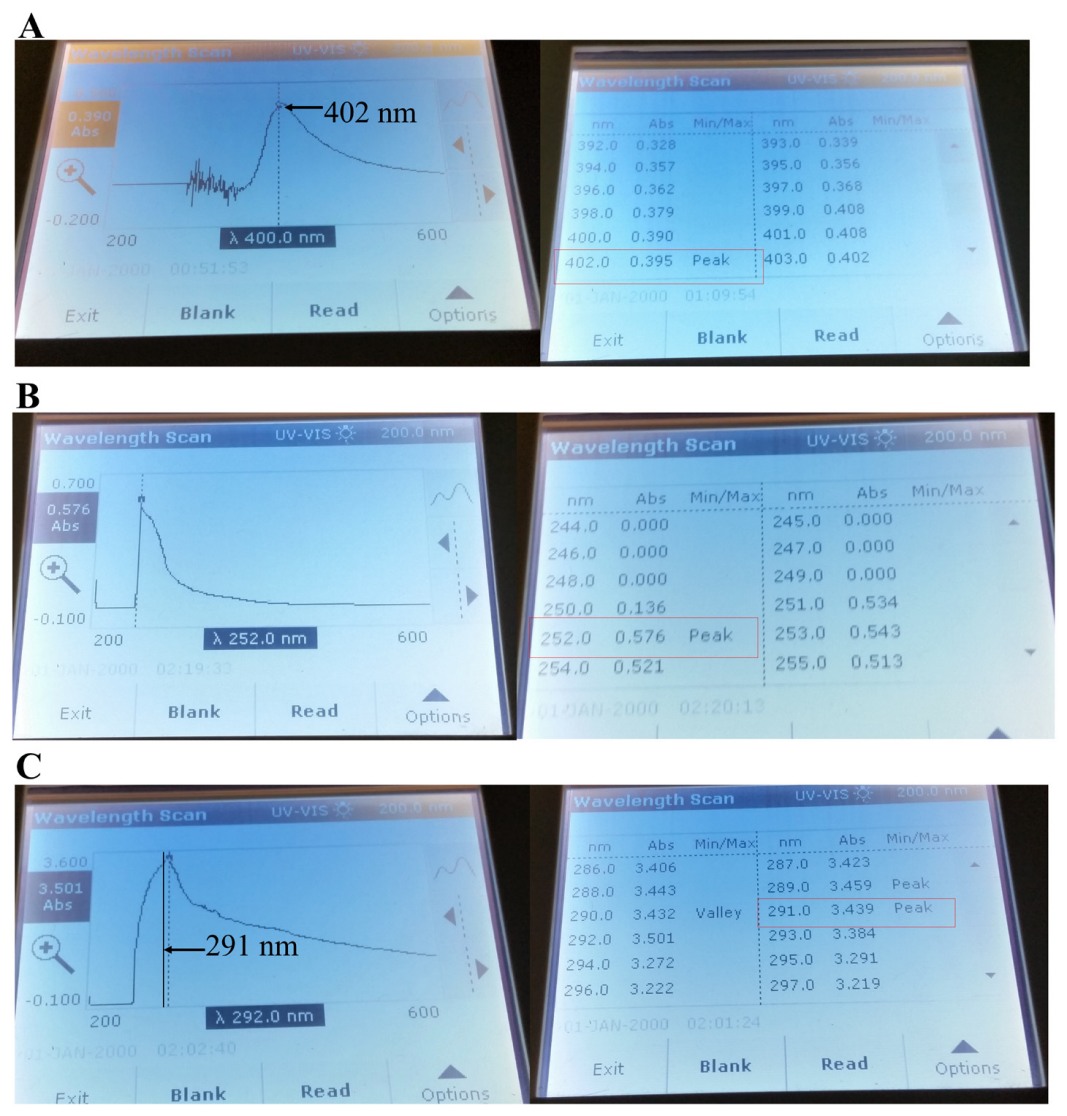
Absorption spectra of chemicals and pigment in LMJ. ( **A**) Absorption curve of the pigment, exuded out in the LMJ LB liquid culture at 37ºC and the corresponding absorbance reading and the absorption peak. ( **B**) Absorption curve of the acidified LMJ cell pellet wash and the corresponding absorbance reading and the absorption peak. LMJ cells were harvested from the LB liquid culture (72 hours old) by centrifugation. ( **C**) Absorption curve of the alkalinized LMJ cell pellet wash and the corresponding absorbance reading and the absorption peak. LMJ cells were harvested from a LB-agar medium plate. Absorption maxima of the alkalinized LB medium ( **A**), acidified ( **B**) and alkalinized ( **C**) cell pellet washes were measured using the wavelength scan program in the UV-visible light wavelength range of 200–600 nm in a UV-Vis spectrophotometer. The absorbance readings at the absorption peaks are outlined by the red box. The black arrows and the black line points to the absorption maxima in the absorption curve.

Pyomelanin absorption peak is between 400 and 405 nm ^45– 47^. We observed a 402 nm absorption peak in the absorption curve of alkalinized LMJ liquid LB culture medium ( Figure 8A). BQA absorbs maximally in the UV region at 250 nm ^45^. We observed an absorption peak at 252 nm in the absorption curve of acidified-cell pellet wash supernatant derived from LMJ liquid LB culture ( Figure 8B). HGA absorbs maximally in the UV region at 290 nm ^45^. We observed an absorption peak at 291 nm in the absorption curve of alkalinized cell pellet wash supernatant derived from LMJ cells from a LB-agar medium plate ( Figure 8C). A slight 1-2 nm shift was observed in the HGA and BQA absorption peaks in our samples compared to the values found in literature (see *Discussion*) ^45^. Taken together our results strongly indicate the LMJ produces pyomelanin and its production is stimulated under light by tryptone ( Figure 6 and Figure 8). We did not have tyrosine at the time of submission of this work.

### LMJ growth on MAC and MSA

**Figure 9.**
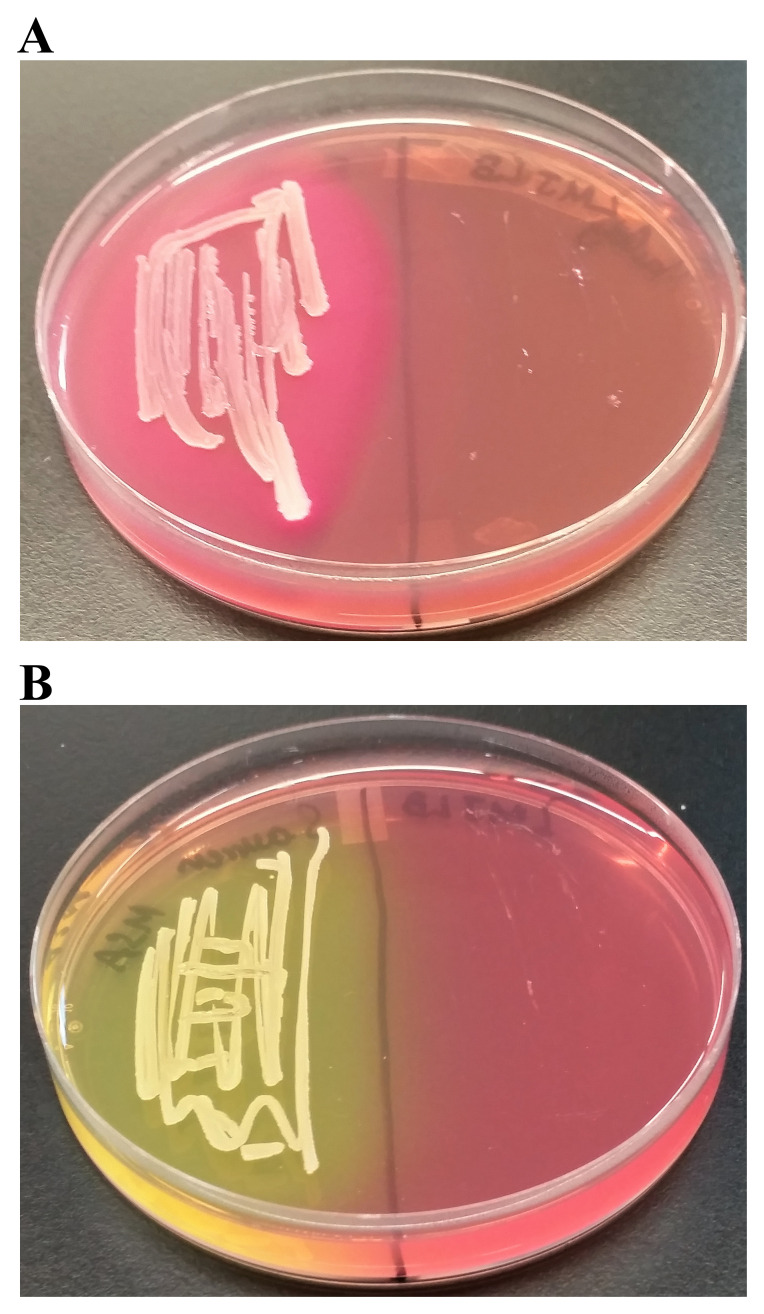
LMJ growth on MacConkey Agar and Mannitol Salt Agar. ( **A**) LMJ (on right) and *Escherichia coli* (left) on MacConkey Agar medium plate. LMJ fails to grow on MacConkey agar medium plate. *E. coli* appears pinkish because it ferments lactose to acid, which causes the neutral red pH indicator to turn red. The dark opaque pink haze on the medium around the *E. coli* growth is the bile precipitation in acidic environment ( **B**) LMJ (on right) and *Staphylococcus aureus* (left) on Mannitol Salt Agar medium plate. LMJ fails to grow on Mannitol Salt Agar medium plate. Culture plates were imaged after 4 days of growth at room temperature.

MAC is a selective and differential medium used for isolation and differentiation of enteric gram-negative bacteria based on their ability to ferment lactose. Bile salts and crystal violet in MAC inhibit the growth of gram-positive bacteria. Lactose in MAC is a source of fermentable carbohydrate. Neutral red is a pH indicator present in MAC that turns red at a pH below 6.8 and is colorless at a pH greater than 6.8. Bacteria that ferment lactose and thereby produce acid in the medium will appear pink because of the neutral red turning red while bacteria that are lactose non-fermenters will produce normal-colored or colorless colonies. LMJ was unable to grow on MAC ( Figure 9A; right) unlike *E. coli,* a gram-negative enteric bacterium ( Figure 9A; left). *E. coli* appeared pink on MAC plate, indicating it can ferment lactose ( Figure 9A; left). Additionally, bile salts precipitated out of the MAC medium surrounding the growth of *E. coli* because of the acidic pH ( Figure 9A; left). Our results indicate that LMJ is a non-enteric bacterium. Inability of LMJ to grow on MAC could be because: 1) LMJ cannot use lactose as a carbon/energy source; 2) because it is sensitive to bile salts and crystal violet in the MAC medium or 3) a combination of 1 and 2.

MSA is a selective and differential medium. MSA contains a high concentration (about 7.5–10%) of NaCl. This makes MSA selective for many high salt-tolerant gram-positive bacteria ( *Staphylococcus*, *Enterococcus* and *Micrococcus*) as the high salt concentration is inhibitory for growth of most bacteria. MSA also contains the sugar mannitol and the pH indicator phenol red. If a bacterium can ferment mannitol to acid, the color of phenol red in the agar will change from red to yellow. LMJ being gram-negative and salt-sensitive ( Figure 2 and Figure 4), fails to grow on MSA ( Figure 9B, right) while *Staphylococcus aureus*, a mannitol-fermenter, grew on MSA and produced acid that changed the phenol red’s color to yellow ( Figure 9B, left).

### Ability of LMJ to use different sugars as an alternative carbon source

**Figure 10.**
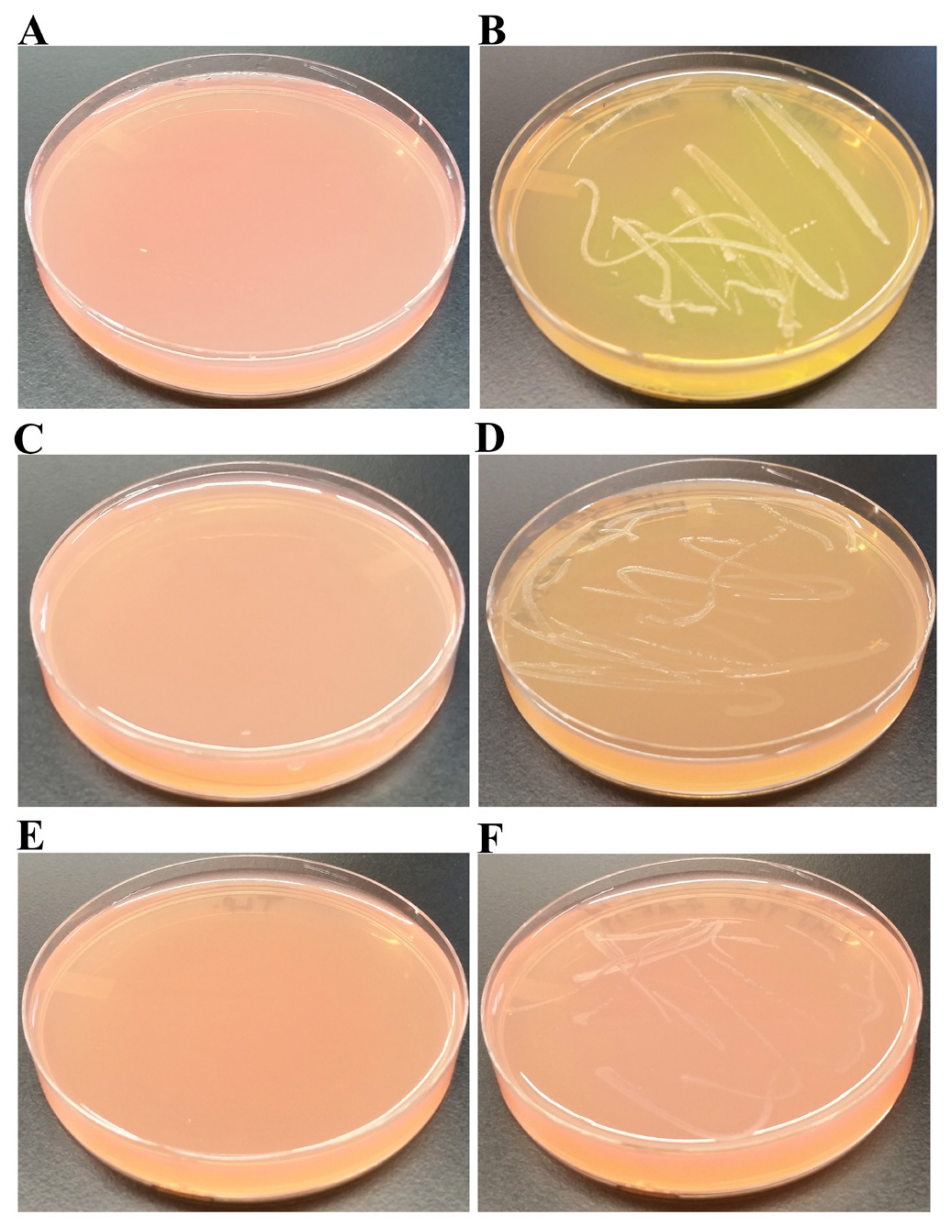
LMJ’s ability to use and ferment different sugars as the sole carbon source. ( **A**) Control TP + 1% glucose agar medium plate with phenol red as a pH indicator. ( **B**) LMJ growth on TP +1% glucose agar medium plate containing phenol red. LMG can ferment glucose to produce acid. ( **C**) Control TP +1% sucrose agar medium plate with phenol red as a pH indicator. ( **D**) LMJ growth on TP +1% sucrose agar medium plate containing phenol red. Trace amount of sugar fermentation detected. ( **E**) Control TP +1% lactose agar medium plate with phenol red as a pH indicator. ( **F**) LMJ fails to grow on TP +1% lactose agar medium plate containing phenol red. Phenol red’s color turns yellow when sugars are fermented to produce acid. Culture plates were imaged after 5 days of growth at room temperature.

We grew LMJ on TP + 1% sugar agar which contained phenol red as the pH indicator. We replaced the carbon source acetate in the TAP medium with sugars like glucose, sucrose and lactose ( Figure 10). TAP minus acetate medium is called TP medium in this work. Figure 10A, Figure 10C and Figure 10E represent control TP + 1% glucose, TP + 1% sucrose and TP + 1% lactose plates, respectively. LMJ grew very well on TP + 1% glucose agar and fermented glucose to produce acid ( Figure 10B). It did grow to some extent on TP +1% sucrose agar but not as well as it did on the TP +1% glucose agar ( Figure 10B, D). It was hard to confirm if LMJ fermented sucrose to any significant degree as there was no distinct color change of phenol red like that in Figure 10B ( Figure 10D). But it is to be noted that the color of phenol red in the control TP +1% sucrose agar was light red ( Figure 10C) and that on LMJ-TP +1% sucrose agar was light orange ( Figure 10D), indicating there could have been some sugar fermentation. No visible growth of LMJ was observed on TP +1% lactose agar. Taken together with results from the LMJ growth on MAC medium ( Figure 9A), LMJ cannot use the disaccharide lactose as a carbon source ( Figure 9A and Figure 10F) but can use monosaccharide glucose and disaccharide sucrose as alternative carbon sources ( Figure 10B, D).

### Starch hydrolysis test

**Figure 11.**
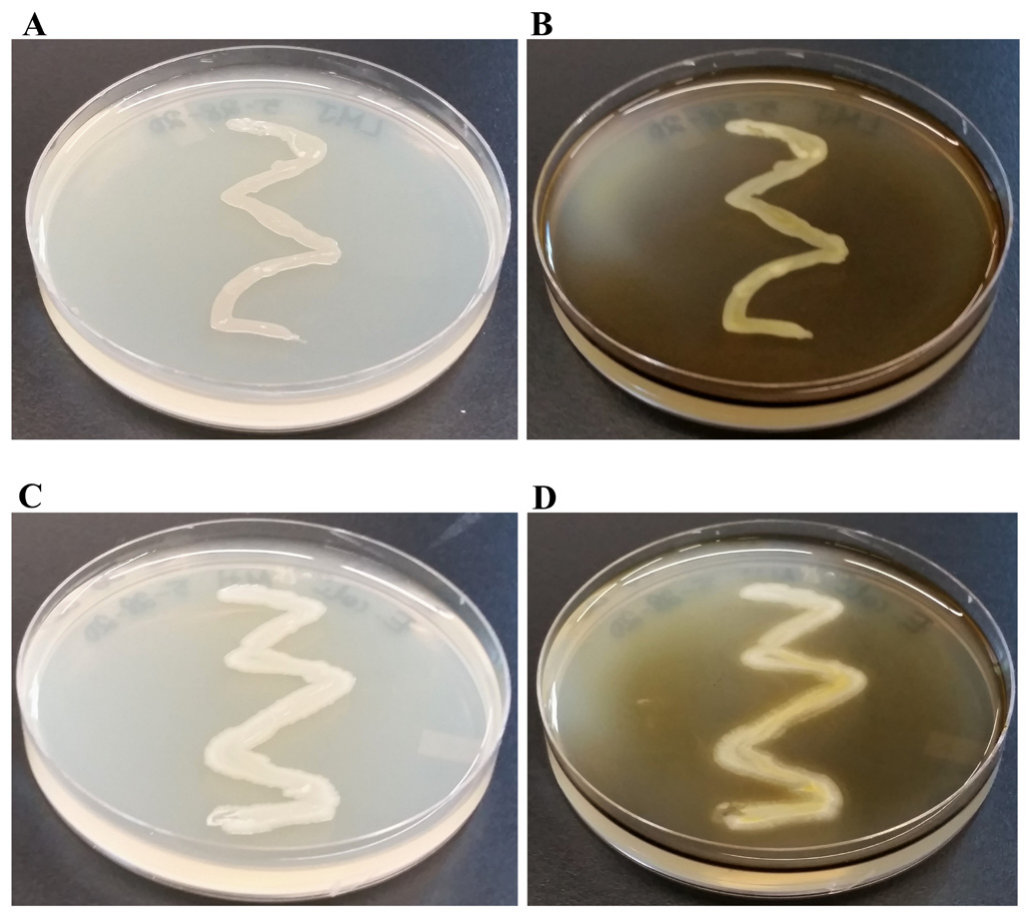
Starch hydrolysis test. ( **A**) 48 hours-growth of LMJ on Mueller-Hinton medium which contains 0.15% starch. ( **B**) LMJ Mueller-Hinton plate shown in ( **A**) was treated with Gram iodine. ( **C**) 48 hours-growth of *E. coli* on Mueller-Hinton medium which contains 0.15% starch. ( **D**) *E. coli* Mueller-Hinton plate shown in ( **C**) treated with Gram iodine. *E. coli* and LMJ fail to hydrolyze starch on Mueller-Hinton medium as there are no visible clear zones around the bacterial growth after gram iodine treatment. The brown-blue color of the medium upon Gram iodine treatment occurs because of the reaction of starch in the medium with iodine. Mueller-Hinton medium plates were incubated with Gram iodine for 10 minutes at room temperature and then imaged.

Starch hydrolysis test is used to identify bacteria that can hydrolyze starch (amylose and amylopectin) using the enzymes α-amylase and oligo-1,6-glucosidase. Because of the large size, amylose and amylopectin molecules cannot cross the bacterial cell wall. To use starch as a carbon source, bacteria must secrete α-amylase and oligo-1,6-glucosidase into the extracellular space. These enzymes break the starch molecules into smaller glucose subunits which can be utilized by the cells. To interpret the results of the starch hydrolysis test, Gram iodine was added to the agar. Iodine reacts with the starch to form a dark brown/blue color. Hydrolysis of starch will create a clear zone around the bacterial growth. Both LMJ ( Figure 11B) and *E. coli* ( Figure 11D) tested negative in the starch hydrolysis test as there was no clear zone around the growth on the Mueller Hinton agar. We did not have an access to a starch-positive strain to use as a positive control in the starch hydrolysis test.

### Oxidase test

**Figure 12.**
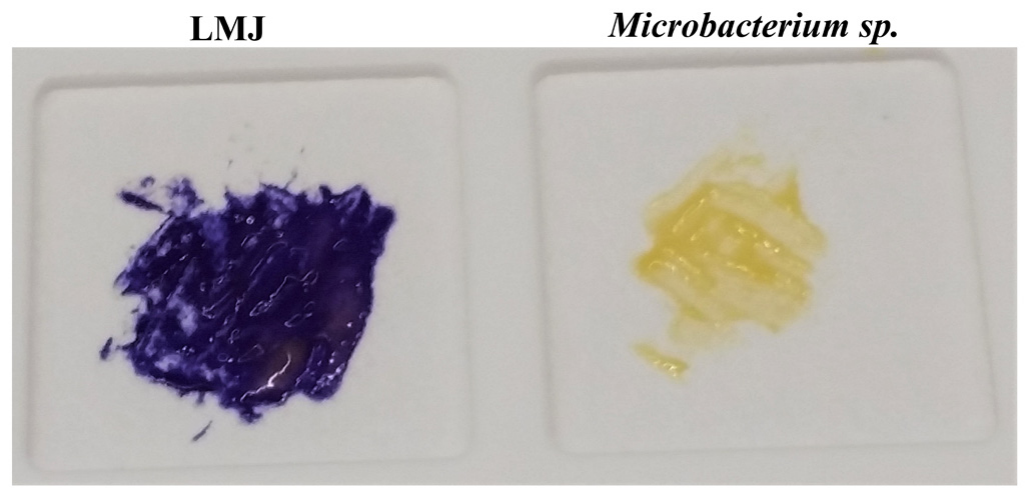
Cytochrome c oxidase test. Cells of LMJ (on the left) and *Microbacterium* sp. (on the right) streaked on a disposable slide containing a film coated with oxidase reagent (tetramethyl-p-phenylenediamine dihydrochloride). Image of the slide was taken after 10 seconds of the application of the cells on the slide. LMJ is cytochrome c oxidase-positive as cytochrome c oxidase, if present, oxidizes the oxidase reagent on the film to form purple colored-indophenols. *Microbacterium* sp. *,* a yellow-pigmented bacterium, is oxidase-negative and fails to form the purple-colored product. Cells were taken from strain specific-tryptic soy agar medium plates.

The oxidase test is used to identify aerobic, facultative anaerobic or microaerophilic bacteria that produce cytochrome c oxidase, an enzyme of the electron transport chain. When present, cytochrome c oxidase oxidizes the oxidase reagent (tetramethyl-p-phenylenediamine; TMPD) to indophenols (purple colored product) within 5–10 seconds. When the enzyme is not present, the reagent remains reduced and is colorless. Oxidase-positive strains take 5–10 seconds to form indophenols and the color change occurs within 30 seconds. Delayed oxidase-positive strains form indophenols within 60–90 seconds and oxidase-negative strains can either remain colorless or take more than 2 minutes to show the purple color because of slow spontaneous non-enzymatic oxidation of TMPD in air ( https://www.asm.org/Protocols/Oxidase-Test-Protocol). Our oxidase test results show that LMJ is oxidase-positive ( Figure 12; left) and *Microbacterium* sp. is oxidase-negative ( Figure 12; right). Hence LMJ is an aerobic bacterium and uses cytochrome c in the electron transport chain ( Figure 3– Figure 6, Figure 8 and Figure 12).

### Testing of antibiotic-sensitivity of *Chlamydomonas* and LMJ to identify an antibiotic that will eliminate LMJ contamination of *Chlamydomonas*

We tested four antibiotics to determine which of these four antibiotics and at what dose, will be effective in inhibiting growth of LMJ with no/minimal detrimental effect on *Chlamydomonas* growth. In the first set of experiments, we determined the antibiotic-sensitivity of LMJ and *Chlamydomonas* 4A+ strain separately to identify the effective antibiotic and, the required dose. In the second set of experiments, we streaked *Chlamydomonas* and LMJ together on the TAP-agar medium plate containing the antibiotic with the effective dose that we found to be potent against LMG (without hindering *Chlamydomonas* growth) from the first experiment. This was done to confirm that the selected antibiotic is effective in eliminating LMJ contamination on *Chlamydomonas* TAP-agar plates.

We modified the traditional KB disc diffusion method to test for antibiotic-sensitivity as described under Method section. We tested two different doses (50 µg and 100 µg) of penicillin, polymyxin B, neomycin and chloramphenicol. Table 2 shows the average diameter of the zone of inhibition for each antibiotic dose, with the standard deviations. Statistical analyses are available as part of the *Underlying data* ^43^. Images of all antibiotic plates are available as *Underlying data* ^44^.

**Table 2.** Mean diameters of zone of inhibitions obtained using the disc-diffusion antibiotic susceptibility test. Zones of inhibitions in the presence of four different antibiotics were studied for *Chlamydomonas reinhardtii* and the bacterial strain, LMJ. Grey and white rows represent 50 µg and 100 µg dose of each antibiotics applied on the filter paper discs, respectably. Three biological replicates were used to calculate the mean and standard deviations shown in the table ( 10.6084/m9.figshare.12407735; 10.6084/m9.figshare.12407741).

Antibiotic	*C. reinhardtii*	LMJ
**Penicillin**	0 mm ± 0	33.3 mm ± 0.2
	0 mm ± 0	40.3 mm ± 0.1
**Polymyxin *B***	8.5 mm ± 0.2	9.5 mm ± 0.1
	9.6 mm ± 0.4	10.4 mm ± 0.4
**Neomycin**	9.5 mm ± 0.5	9.5 mm ± 0.1
	11.1 mm ± 0.1	12.7 mm ± 0.1
**Chloramphenicol**	0 mm ± 0	28.8 mm ± 0.1
	0 mm ± 0	33.3 mm ± 0.1

**Figure 13.**
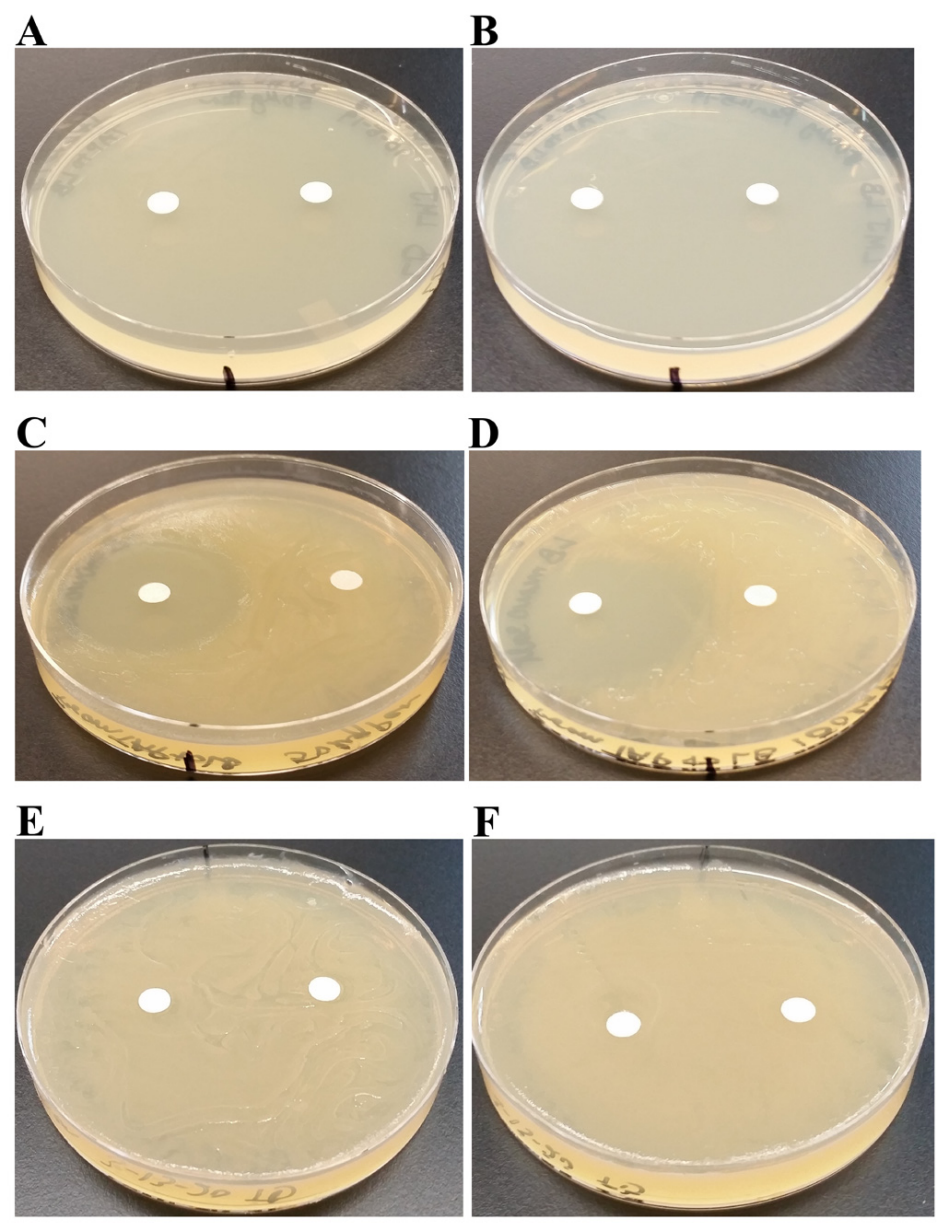
Comparison of penicillin-sensitivity of LMJ and *E. coli* on LB agar medium. On the medium plates shown in ( **A**– **F**), the filter paper disc on the right side is the water control disc and the one on the left side is the antibiotic disc containing either 50 µg (82.5 units) or 100 µg (165 units) of penicillin. Plates were imaged after incubation at 22ºC for 4 days. ( **A**) LMJ on LB-agar medium plate and the antibiotic disc contains 50 µg of penicillin. ( **B**) LMJ on LB-agar medium plate and the antibiotic disc contains 100 µg of penicillin. ( **C**) LMJ on LB minus 1% NaCl-agar medium plate and the antibiotic disc contains 50 µg of penicillin. ( **D**) LMJ on LB minus 1% NaCl-agar medium plate and the antibiotic disc contains 100 µg of penicillin. ( **E**) *E. coli* on LB-agar medium plate and the antibiotic disc contains 50 µg of penicillin. ( **F**) *E. coli* on LB-agar medium plate and the antibiotic disc contains 100 µg of penicillin. Antibiotic plates were imaged after 3 days of growth.

*a) Penicillin-sensitivity testing:* Penicillin affects peptidoglycan biosynthesis and compromises cell wall integrity in gram-positive bacteria. *Chlamydomonas* was resistant to both 50 µg (82.5 IU units) and 100 µg (165 IU units) as it did not show any zone of inhibitions ( Table 2). LMJ was highly sensitive to penicillin ( Table 2), despite being a gram-negative bacterium. p-values for the one-tailed and two-tailed hypothesis test for both doses were infinitely small and statistically significant. To ensure that the penicillin-sensitivity of LMJ is not because of the TAP-agar medium used in our experiments, we tested penicillin-sensitivity of LMJ and *E. coli* (control) on LB-agar medium and LB-1% NaCl agar medium using 50 µg and 100 µg of penicillin ( Figure 13). We found that LMJ did not grow on LB-agar in the presence of penicillin ( Figure 13A, B). LMJ grew on LB-1% NaCl + penicillin plates and showed a prominent zone of inhibition like it showed on TAP-agar penicillin plates ( Figure 13C, D). *E. coli* was not sensitive to either of the two penicillin doses ( Figure 13E, F). We have shown in Figure 4A, E that 1% NaCl inhibits growth of LMJ at 22°C. Hence penicillin in combination with 1% NaCl completely prevented growth of LMJ on LB-agar + penicillin plates ( Figure 13A, B). We did not measure the diameter of the inhibition zones as this experiment was performed to compare penicillin sensitivity of two gram-negative bacteria, namely *E. coli* and LMJ on LB agar medium.

*b) Polymyxin B-sensitivity testing:* Polymyxin B binds to the lipopolysaccharide of the outer membranes of gram-negative bacteria and increases the permeability of the bacterial outer membrane, which causes cell death. At 50 µg (500 IU) dose, LMJ is more sensitive to polymyxin B than *Chlamydomonas* ( Table 2). The p-values for the one-tailed and two-tailed tests for the 50-µg dose were 0.4% and 0.09%, respectively, indicating that the polymyxin B sensitivity of LMJ is statistically significant. The p-values for the one-tailed and two-tailed tests for the 100-µg dose were 6.4% and 12.9%, respectively indicating that there was no significant difference in polymyxin b-sensitivity between *Chlamydomonas* and LMJ ( Table 2).

*c) Neomycin-sensitivity testing:* Neomycin inhibits protein translation by binding to the 30S subunit of bacterial ribosomes. At 50 µg dose, there was no statistically significant difference in neomycin-sensitivity between *Chlamydomonas* and LMJ as the p-values for the one-tailed and two-tailed tests for the 50 µg dose were 50% and 100%, respectively. At 100 µg dose, LMJ was more sensitive to neomycin than *Chlamydomonas* ( Table 2). The p-values for the one-tailed and two-tailed tests for the 100-µg dose were 0.2% and 0.5 % respectively, indicating that there was a significant difference in neomycin-sensitivity between *Chlamydomonas* and LMJ ( Table 2).

d) *Chloramphenicol-sensitivity testing:* Chloramphenicol inhibits protein synthesis by binding to the 50S subunit of bacterial ribosomes. *Chlamydomonas* was resistant to both 50 µg and 100 µg of chloramphenicol as it did not show any zone of inhibitions ( Table 2). LMJ was highly sensitive to chloramphenicol compared to *Chlamydomonas* ( Table 2).

**Figure 14.**
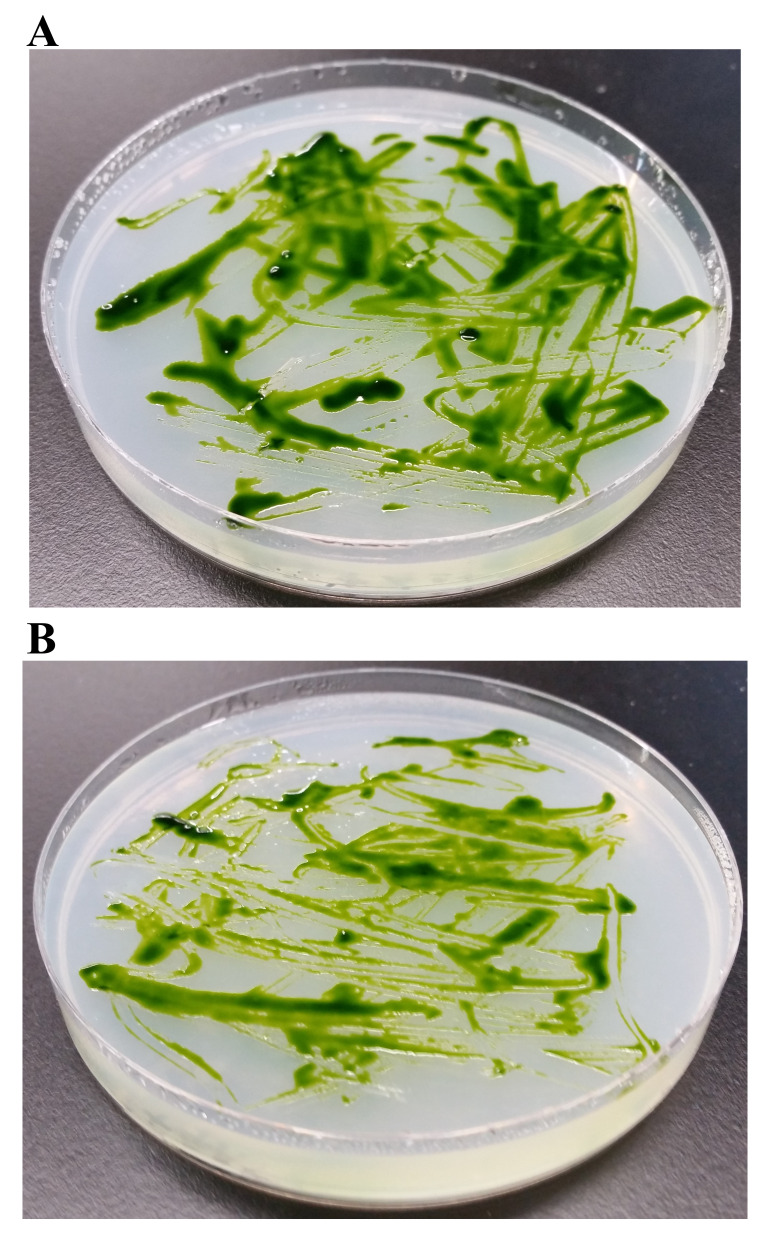
Testing the efficacy of penicillin and chloramphenicol in minimizing LMJ contamination on *Chlamydomonas* TAP-agar plates. ( **A**) *Chlamydomonas* strain 4A+ and LMJ strain streaked on TAP-agar plate containing 50 µg of penicillin/mL of TAP medium. ( **B**) *Chlamydomonas* strain 4A+ and LMJ strain streaked on TAP-agar plate containing 50 µg chloramphenicol/mL of TAP medium. TAP-agar antibiotic plates were incubated at room temperature for 2 weeks before they were imaged.

*e) Testing the potency of penicillin and chloramphenicol in minimizing LMJ contamination on Chlamydomonas culture plates:* Our first set of experiments determined penicillin and chloramphenicol are the best antibiotic choices for minimizing LMG contamination. We tested combined growth of LMJ and the wild type *Chlamydomonas* strain 4A+ on TAP agar plate containing 50 µg penicillin/mL of the medium ( Figure 14A) and on TAP-agar plate containing 50 µg chloramphenicol/mL of the medium ( Figure 14B). LMJ growth was not visible on both penicillin and chloramphenicol TAP plates ( Figure 14).

### PCR amplification of the partial 16S rRNA gene of LMJ

**Figure 15.**
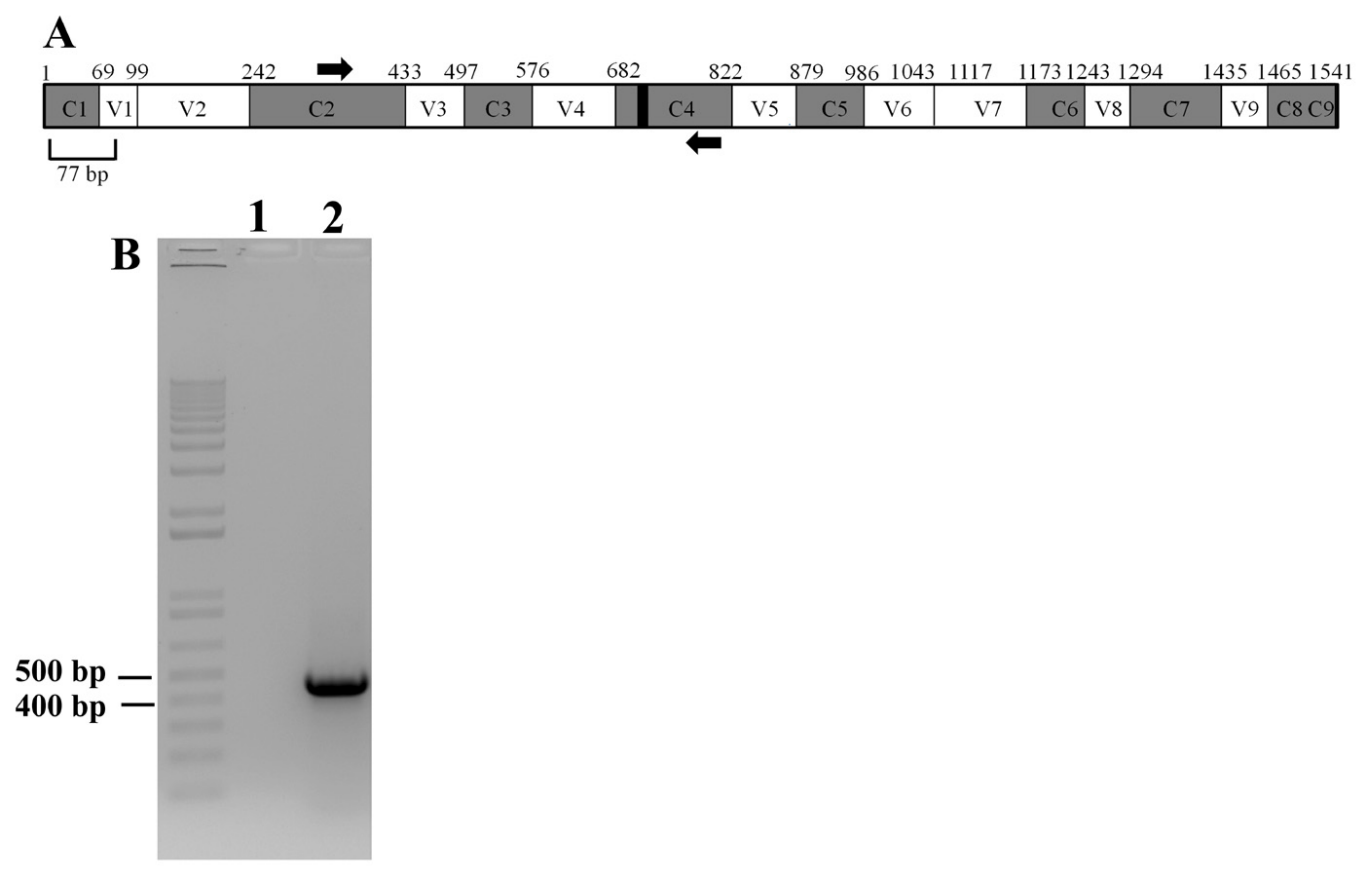
Amplification of 16S rRNA partial gene sequence of LMJ. ( **A**) A schematic diagram showing the conserved and hypervariable regions in the 16S rRNA gene. The interspersed conserved regions (C1–C9) are shown in gray, and the hypervariable regions (V1–V9) are depicted in white. The black box within the C4 region represents 11 nucleotides (788 -798 base pairs) that are invariant in bacteria. PCR primers are shown in thick black arrows. Forward primer is in the C2 region and the reverse primer is in the C4 region. The figure is based on the 16S rRNA gene sequence of *E. coli*. ( **B**) A DNA agarose gel showing the results of PCR with the primers shown in ( **A**). Lane 1 represents PCR with water (zero DNA control) and Lane 2 showing the PCR product (approximately 460 bp in size) amplified by the PCR primers. 1kb plus DNA ladder was used as a DNA molecular size ladder.

Figure 15A shows a schematic diagram of a full-length 16S rRNA gene based on the *E. coli* 16S rRNA gene. The nine hypervariable regions, V1- V9, spanned nucleotides 69-99, 137-242, 433-497, 576-682, 822-879, 986-1043, 1117-1173, 1243-1294 and 1435-1465, respectively ^54– 56^. 11 nucleotides (788-798) within C4 are invariant in bacteria and is represented in the schematic as a black box within the C4 region ^57^. The forward PCR primer, 16SF, spans nucleotides 340-356 in the C2 region and the reverse PCR primer, 16SR, spans nucleotides 784-804 within the C4 region of 16S rRNA gene ( Figure 15A). Figure 15B shows that PCR amplification of the partial 16S rRNA gene generated an amplicon of approximately 460 bp in size ( Figure 15A).

### NCBI-BLAST analyses of the 16S rRNA partial gene sequence of LMJ

**Figure 16.**
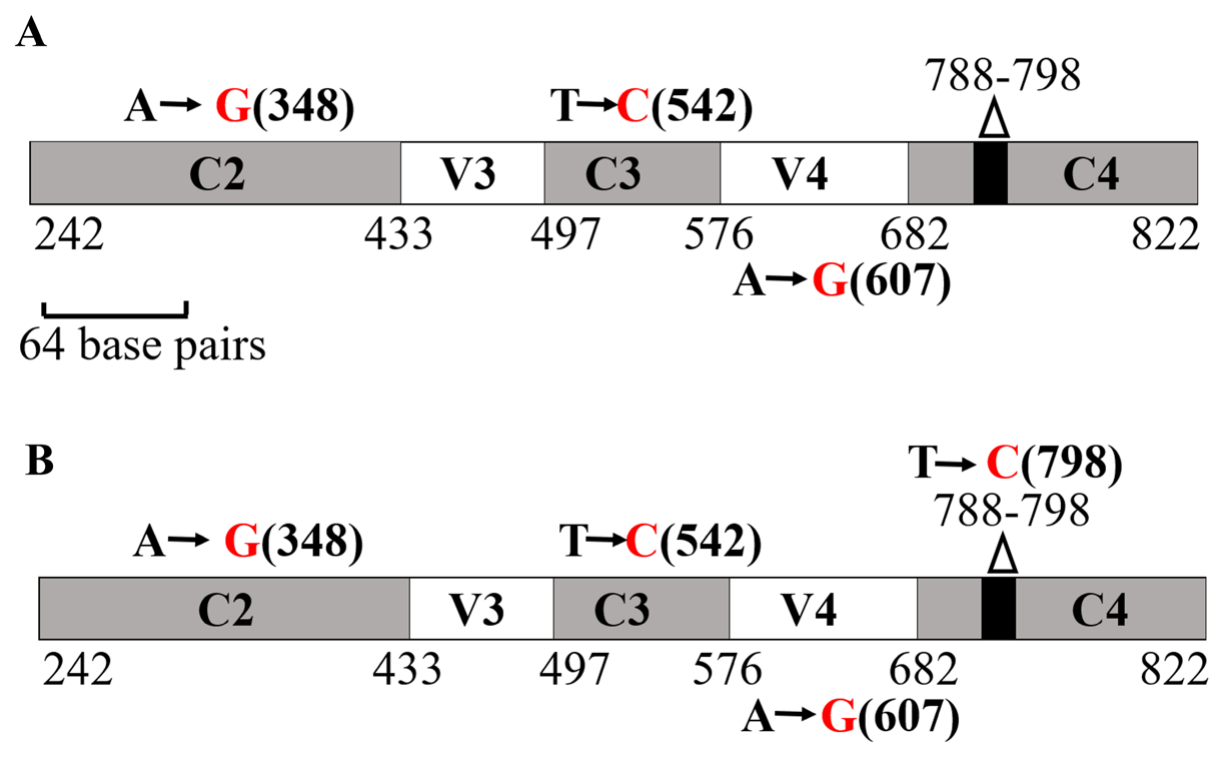
NCBI-BLAST analyses of the 16S rRNA partial gene sequence of LMJ. ( **A**) A schematic diagram showing the nucleotide changes in LMJ in the 16S rRNA region spanning the C2 and C4 regions in comparison to the best NCBI- BLAST hit (score of 843; E-value 0 and percent identity of 99.35%): Uncultured bacterium clone LIB091_C05_1243 16S ribosomal RNA gene partial sequence (Accession #: JX086489.1). ( **B**) A schematic diagram showing the nucleotide changes in LMJ in the 16S rRNA region spanning the C2 and C4 regions in comparison to a second best BLAST hit with a genus name (score of 837; E-value 0 and percent identity of 99.14%): *Acidovorax* sp. strain A16OP12 16S ribosomal RNA gene, partial sequence (Accession #: MN519578.1). Conserved regions and the hypervariable regions are depicted in grey and white, respectively. The invariant 11 bp region (788 -798 base pairs) within the C4 region is shown by a black box within the gene. Black nucleotides show the native nucleotides in the BLAST hit that were substituted by the depicted red nucleotides in LMJ 16S rRNA gene sequence. The black bold numbers within the parenthesis beside the nucleotides show the specific nucleotide position where the nucleotide changes have occurred. Nucleotide positions shown in the figures have been assigned according to that of the 16S rRNA gene sequence of *E. coli.*

The amplicon shown in Figure 15B was sequenced to determine the nearest relative of LMJ. NCBI-nucleotide BLAST analyses identified the best match to LMJ’s 16S rRNA gene partial sequence as that of an uncultured bacterium clone LIB091_C05_1243 16S ribosomal RNA gene partial sequence (Accession #: JX086489.1). This hit had a score of 843; 0 E-value and percent identity of 99.35%. Figure 16A shows three nucleotide substitutions (transitions) that are present in LMJ 16S rRNA gene sequence relative to uncultured bacterial clone hit (altered nucleotide in LMJ is shown in red font). Two of these nucleotide substitutions are in the conserved regions, C2 (nucleotide position 348) and, C3 (nucleotide position 542) and the third nucleotide substitution is in the V4 region (nucleotide position 607) ( Figure 16A).

We found that the nearest relative of LMJ with a specific genus name is *Acidovorax* sp. strain A16OP12 16S (Accession #: MN519578.1). *Acidovorax* sp. hit had a score of 837, 0 E-value and 99.14% sequence identity. In addition to the above stated three nucleotide substitutions, a fourth nucleotide substitution (another transition at nucleotide position 798) within the 11 bp invariant region in the C4 region was observed, when LMJ’s partial 16S rRNA sequence was compared with that of the *Acidovorax* sp. strain A16OP12 (Accession #: MN519578.1) ( Figure 16B). We have deposited the partial 16S rRNA sequence of LMJ in GenBank with the definition: Bacterium strain clone LIB091_C05_1243 variant 16S ribosomal RNA gene, partial sequence (Accession number: MN633292.1). Raw electropherogram files and sequence text files are available as *Underlying data* ^49^.

### Ability of LMJ to use PAHs, saturated hydrocarbons and PHA as the sole carbon source

*Acidovorax* sp. utilize alkanes, PAH and PHA as alternative carbon sources for growth ^30– 38^. Since the nearest relative of LMJ is *Acidovorax*, we tested if LMJ can utilize the above mentioned chemical compounds as a carbon source for growth. TP medium (lacks a carbon source) was supplemented with the following three different categories of external carbon sources in separate experiments: 1) saturated alkane hydrocarbons, 2) aromatic compounds (includes PAH) and, 3) PHA: PHB. Images of all medium plates are available as *Underlying data* ^40^.

**Figure 17.**
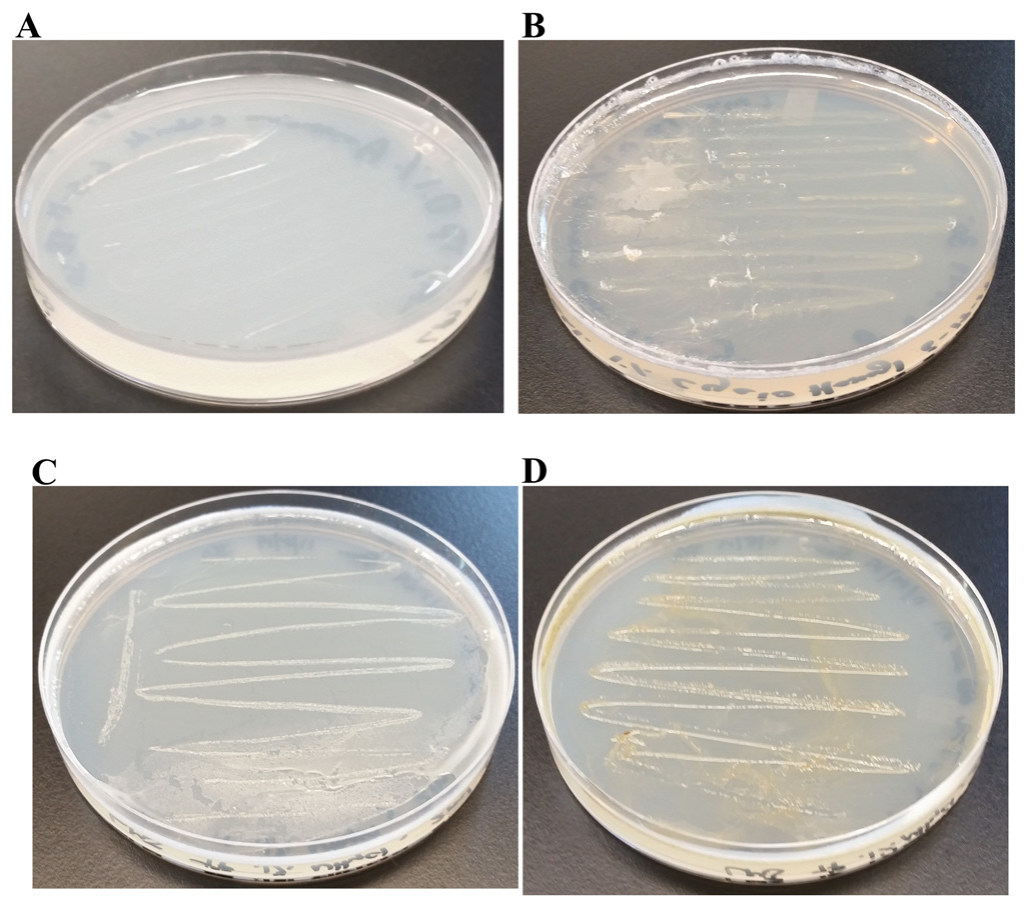
Ability of LMJ to use hydrocarbons as a sole carbon source. Tris-Phosphate (TP) agar medium plates shown in Figure 17B-17D were coated with different hydrocarbons. LMJ was streaked on the control TP-agar medium plate and on the hydrocarbon-coated TP-agar medium plates. After 2 weeks of growth at room temperature, medium plates were imaged. ( **A**) TP-agar medium plate streaked with LMJ. LMJ does not grow on TP medium as it lacks a carbon source. ( **B**) LMJ growth on TP-agar medium plate coated with 4 mL of 1% cyclohexyl chloride diluted with chloroform. ( **C**) LMJ growth on TP-agar medium plate coated with 2 mL of 2% (v/v) fresh 10W-30 car motor oil. ( **D**) LMJ growth on TP-agar medium plate coated with 2 mL of 2% (v/v) combusted 10W-30 car motor oil.

*a) Testing hydrocarbons (cycloalkanes and 10W30 oil) as the sole carbon source:* LMJ was streaked on TP-agar plate as a control to show that LMJ does not grow on a TP medium plate ( Figure 17A). 1% cyclohexyl chloride ( Figure 17B), 2% (v/v) fresh 10W30 motor oil ( Figure 17C) and 2% (v/v) combusted 10W30 motor oil ( Figure 17D) were used as carbon sources. We tested one dose of 1% cyclohexyl chloride (4 mL) and three different doses (0.5, 1 and 2 mL) of the 2% (v/v) stock solutions of the fresh and combusted 10W30 motor oil. In Figure 17, we are presenting the results of use of only one dose (2 mL) per types of motor oil. LMJ can grow well on TP-agar media plates coated with 4 ml of 1% cyclohexyl chloride ( Figure 17B), and 2 mL of 2% fresh and 2% combusted 10W30 motor oil as a carbon source ( Figure 17C, D).

*b) Testing PAH and other aromatic compounds as the sole carbon source:* Phenanthrene and napthalene are PAHs. Anthropogenic releases of benzoic acid and sodium benzoate into the environment are primarily from their uses as preservatives. Phenylacetate is found in the environment as a common carbon source and is a central intermediate in the degradation of many aromatic compounds such as phenylalanine, phenylacetaldehyde, 2-phenylethylamine and environmental contaminants like styrene and ethylbenzene ^58^.

**Figure 18.**
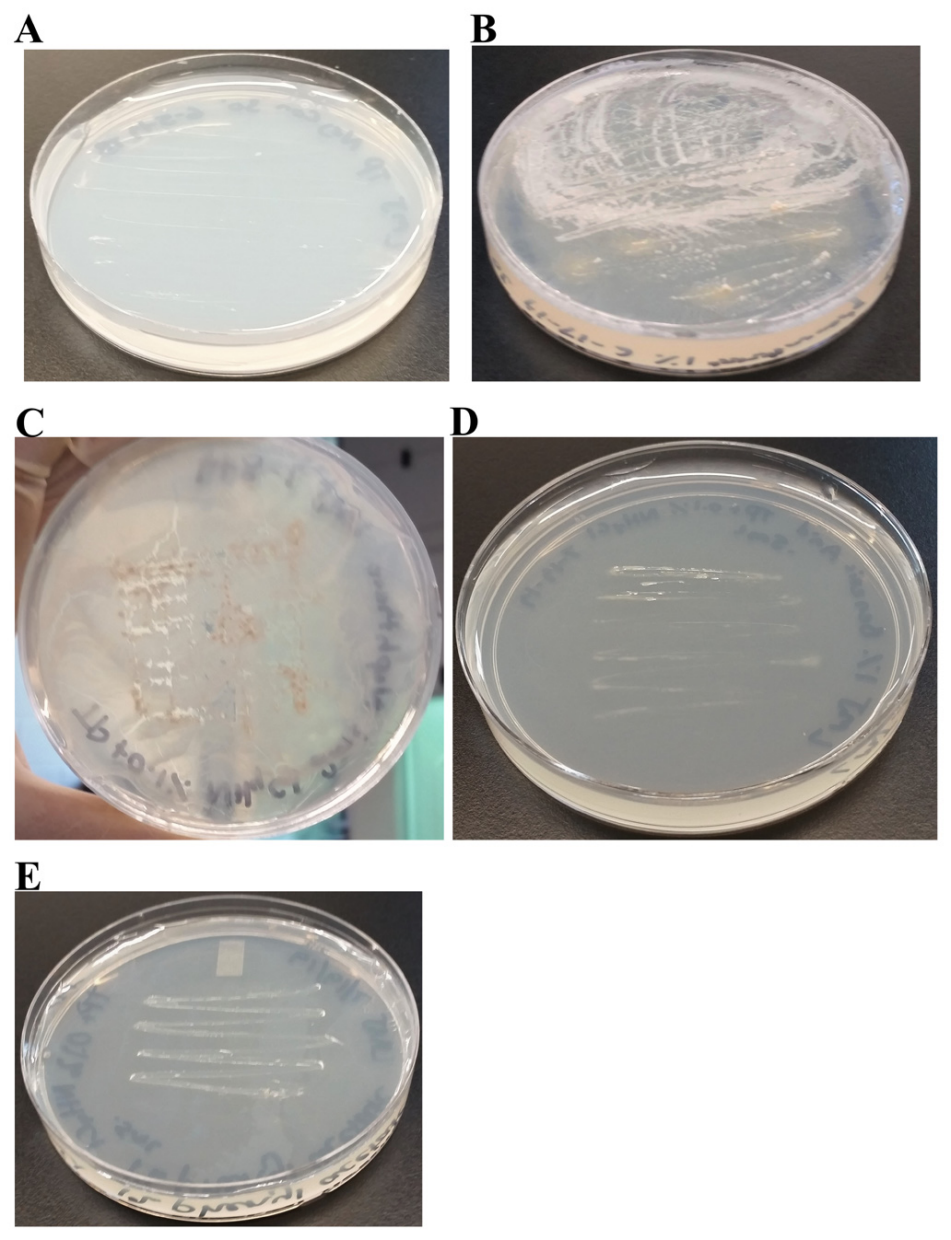
Ability of LMJ to use aromatic compounds as the sole carbon source. TP medium plates shown in panels ( **B**– **E**) were coated with different polycyclic and monocyclic aromatic compounds. LMJ was streaked on the control TP-agar medium plate and on the aromatic compound-coated TP-agar medium plates. After 2 weeks of growth at room temperature, medium plates were imaged. ( **A**) TP plate streaked with LMJ. LMJ does not grow on TP plate as it lacks a carbon source. ( **B**) LMJ growth on TP plate coated with 4 mL of 1% phenanthrene dissolved in chloroform. ( **C**) LMJ growth on TP plate coated with 2 mL of 1% naphthalene dissolved in chloroform. ( **D**) LMJ growth on TP plate coated with 0.5 mL of 1% benzoic dissolved in chloroform. ( **E**) LMJ growth on TP plate coated with 0.5 mL of 1% phenyl acetate dissolved in chloroform.

LMJ was streaked on the TP plate (lacks a carbon source) ( Figure 18A). 1% phenanthrene ( Figure 18B), 1% napthalene ( Figure 18C), 1% benzoic acid ( Figure 18C) and 1% phenyl acetate ( Figure 18D) were used as carbon sources in TP medium plates. We tested two doses of phenanthrene (2 and 4 mL) and three different doses (0.5, 1 and 2 mL) of napthalene, benzoic acid and phenyl acetate. LMJ can grow on TP-agar media plates coated with 4 mL doses of 1% phenanthrene ( Figure 18B), 2 mL of 1% naphthalene ( Figure 18C), 0.5 mL of 1% benzoic acid ( Figure 18D) and 0.5 mL of 1% phenyl acetate as a sole carbon source ( Figure 18E).

**Figure 19.**
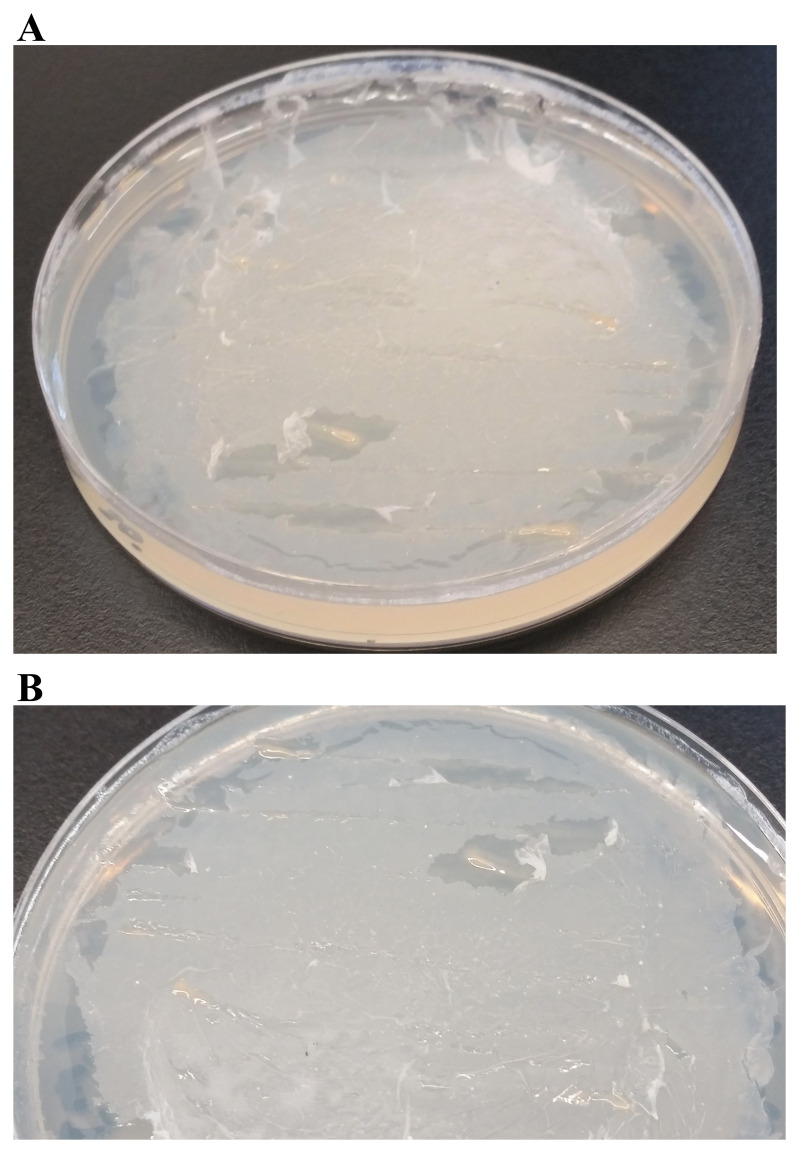
Ability of LMJ to use polyhydroxyalkanoate as the sole carbon source. ( **A**) LMJ growth on TP-agar medium plate coated with 4 mL of 1% polyhydroxybutyrate (PHB). ( **B**) Zoom up of the TP + PHB plate shown in ( **A**). TP-agar medium plate (lacking a carbon source) shown in Figure 18A served as the negative control for this experiment. Plate was imaged after two weeks of growth at room temperature.

*c) Testing PHB as the sole carbon source:* PHB is a polymer belonging to the polyesters class (PHA) that are of interest as bio-derived and biodegradable plastics ^32^. We tested one dose of 1% PHB (4 mL) on the TP-agar plate ( Figure 19). LMG can grow on TP + PHB agar but it grows slowly ( Figure 19).

## Discussion

LMJ is a mesophilic, acetate-loving, pigmented, gram-negative rod. It is a salt-sensitive, oxidase-positive, starch hydrolysis-negative, non-enteric bacterium ( Figure 1– Figure 12). LMJ is gamma hemolytic (results available as *Underlying data* ^59^). NCBI-BLAST analyses of the partial 16S rRNA gene sequence of LMJ revealed that its best match is the partial 16S rRNA gene sequence of an uncultured bacterium clone LIB091_C05_1243 16S (Accession #: JX086489.1). The isolation source for this NCBI match is from monochloramine-treated drinking water biofilms ^60^. The second best match to the LMJ’s partial 16S rRNA gene sequence is that of *Acidovorax* sp. strain A16OP12. This *Acidovorax* strain is isolated from a lake sediment in China (NCBI, Accession #: MN519578.1).

*Pseudomonas facilis*, *Pseudomonas delafieldii*, E. Falsen (EF) Group 13, EF Group 16, and several clinical/environmental isolates have been assigned a new genus name, *Acidovorax*, and belongs to the acidovorans (acid-devouring bacteria) rRNA complex in rRNA superfamily III (betaproteobacteria) in the family Comamonadaceae ^19^. Members of these genera are aerobic, mesophilic, gram-negative, oxidase-positive rods, starch-hydrolysis-negative and are commonly found in biofilm communities in soil, water and on plants ^61^. Pyrosequencing analysis of bacterial biofilm communities in a water meter of a drinking water distribution system in Illinois revealed that 58% of betaproteobacteria in the biofilm on the water meter were *Acidovorax* sp. ^62^. Biofilm formation is essential for the virulence of phytopathogenic strains *of Acidovorax* species ^24, 25, 27, 63– 65^. Our results indicate that LMJ forms biofilm ( Figure 5).

Many bacteria produce extracellular and surface-associated components such as membrane vesicles, extracellular DNA and moonlighting cytosolic proteins for which the biogenesis and export pathways are not fully understood ^66^. In *Pseudomonas aeruginosa*, cells undergo explosive cell lysis as a mechanism for the biogenesis of bacterial membrane vesicles and biofilm ^66^. LMJ liquid cultures were inoculated with tooth picks ( Figure 5). We have noticed LMJ growth on the tooth pick that is immersed in the shaking liquid media. In future, we will gram-stain cells from non-shaking liquid cultures to compare with that from the shaking cultures. We can isolate MVs from LMJ liquid cultures and stain it with FM4-64 fluorescent dye to monitor the red fluorescence. FM4-64 shows low fluorescence in water but fluoresces intensely on binding to the membrane ^66^. We can detect extracellular DNA from LMJ liquid culture by staining with TOTO-1 iodide, which is a cell-impermeant, high-affinity nucleic acid stain ^66^. These studies should confirm if LMJ undergoes explosive cell lysis in liquid cultures to form biofilm.

Despite being a gram-negative bacterium, LMJ is very susceptible to penicillin compared to *E. coli* ( Table 2; Figure 13). In a KB test if the diameter of the zone of inhibition is more than 29 mm, with a 10-µg dose (16.5 IU units) of penicillin, then the bacteria is sensitive to penicillin (see *Methods*). We used penicillin doses that were 5- to 10-fold higher than what is usually used in a KB test. There is one report of a multiple-β-lactam-antibiotic-resistant *Acidovorax* sp. strain MR-S7 ^67^.

If the diameter of the zone of inhibition is more than 17 mm, with a 30-µg dose of chloramphenicol in a KB test, the bacterium is susceptible to chloramphenicol ( https://www.flinnsci.com/antibiotic-sensitivity-disk-chloramphenicol-30-g/ab1400/). LMJ is sensitive to chloramphenicol but *Chlamydomonas* is resistant. If the diameter of the zone of inhibition is more than 16 mm, with a 30 µg dose of neomycin in a KB test, the bacterium is susceptible to neomycin ( https://www.flinnsci.com/antibiotic-sensitivity-disks-neomycin-30-g/ab1403/). If the diameter of the zone of inhibition is more than 12 mm with the application of 300 IU of polymyxin B in a KB test, then the bacteria is sensitive to polymyxin B ^68^. We used antibiotic doses of neomycin and polymyxin B were 1.7- to 3.3-fold higher than what is used for KB tests ( Table 2). LMJ is resistant to neomycin and polymyxin B ( Table 2). We have not found any studies to date that have tested the sensitivity of *Acidovorax* sp. to polymyxin B, neomycin and chloramphenicol.

Many phytopathogenic *Acidovorax* strains are pigmented ^18^. LMJ has light pink-brown pigmentation. *Pseudomonas aeruginosa* has water soluble pinkish red pyorubrin pigment and dark brown pyomelanin pigment ^69– 71^. Pyorubrin’s biosynthesis is enhanced by the addition of 1% DL-glutamate in the growth media ^69^. Pyorubrin has a characteristic absorption peak in the visible light at 520 nm ^70^. Pyomelanin is produced as a product of tyrosine catabolism via homogentisate (HGA) ( Figure 7). Pyomelanin biosynthesis needs oxygen and light and can be enhanced by the addition of 0.36–1% tyrosine in the growth medium. Pyomelanin has a characteristic absorption peak in the visible light ranging from 400–405 nm ^46, 47^.

We used 1% tryptone as we did not have access to tyrosine at our lab. Commercial tryptone contains 1.86 grams of tyrosine/100 grams of tryptone ( https://khimexpert.com/wp-content/uploads/2018/12/GCM23-Tryptone.pdf). The 1% tryptone contained 0.186 g of tyrosine/L of liquid medium which is approximately 19- to 54-fold lower than what researchers have used in inducing pyomelanin production in bacteria ^47, 69^. Although 1% tryptone induced pigment production in LMJ on TAP medium ( Figure 6) it was not dark brown like that observed in other bacterial or fungal strains ^5, 46, 47^. In future, we plan to test the enhancement of pigment production in LMJ with the addition of 1% tyrosine in the growth media.

LMJ pigmentation on TAP+1% tryptone in light and in dark and spectrophotometric pigment analyses strongly indicate that the light pink-brown pigmentation in LMJ is due to light-induced HGA-based pyomelanin production ( Figure 6 and Figure 8). In our experiments, we have a very small shift of 1–2 nm in the absorption peaks for BQA and HGA ( Figure 8B, C) because we did not buffer our samples to a pH of 6.7–6.8 with phosphate buffer ^69^. We performed this experiment using a “quick and crude” protocol to get preliminary data before we optimize our experimental protocol according to the protocols found in the literature ^45, 47^. We plan to optimize our protocols for preparing the samples for the spectrophotometric analyses using the method described in the literature ^69^. We would also like to test if the addition of ascorbic acid in the buffered-acidified cell pellet washes can reduce BQA back to HGA via monitoring the shift in absorption maxima from 250 to 290 nm, as described previously ^69^.

Pyomelanin protects cells against oxidative stress ^5, 15^. It would be interesting to probe pyomelanin’s role in oxidative stress protection in LMJ. LMJ growth can be monitored on TAP +1% tyrosine agar plate in the presence/absence of hydrogen peroxide and photo-sensitizers like Rose Bengal, that generates the reactive oxygen species, singlet oxygen, in the presence of light and oxygen ^72^. Experiments can be performed in the absence and presence of inhibitors of 4-hydroxyphenylpyruvate dioxygenase (HPPD) which are herbicides like 2-[2-nitro-4-(trifluoromethyl) benzoyl]-1,3-cyclohexanedione (NTBC) ( Figure 7) ^11, 73^. Additionally, effects of different light intensities, pH, UV light, heat and salt stress on pyomelanin production can be determined.

Two and three nucleotide substitutions in the conserved regions of the 16S rRNA gene were observed in LMJ when compared with that of the uncultured bacterial clone LIB091_C05_1243 and *Acidovorax* sp. strain A16OP12, respectively ( Figure 16). These results show that conserved regions of the 16S rRNA are not really “conserved” and conserved regions of the 16S rRNA gene exhibit considerable variations that need to be considered, when using this gene as a biomarker ^74^.

Different environmental strains of *Acidovorax* sp. exist that can degrade polychlorinated-biphenyls, PAH, plastic films and saturated hydrocarbons (alkanes) which are major environmental pollutants ^30– 38, 75– 79^. In our first preliminary round of experiments, presented in this work, we have shown that at 22°C on TP medium, LMJ can use PAH, aromatic acids/esters, PHB, saturated chloro-alkane and motor oil as the sole carbon source ( Figure 17– Figure 19). Our second round of experiments will focus on optimizing the process of uniformly coating the medium surface with chemicals as we had uneven deposition of chemical crystals on the plates. Uneven coating can affect LMJ’s utilization of aromatic chemicals.

*Acidovorax* sp. *DP5* displays a high extracellular depolymerase enzyme activity when grown in medium containing 0.25% (w/v) of PHB and 1 gram/L of urea as carbon and nitrogen source, respectively ^32^. The depolymerase enzyme produced by strain *Acidovorax* sp. *DP5* showed high percentage of degradation of PHB films in an alkaline condition at pH 9 under a temperature of 40°C ^32^. Our third round of experiments would be to test different types of nitrogen sources, pH and temperature that would allow optimal growth on PHB and PAH-containing TP medium plates. It would be interesting to compare the growth of LMJ on M9 bacterial minimal medium against the growth on TP medium, when testing alternative carbon sources, as M9 medium has a higher phosphate and nitrogen content than the TAP medium, respectively ( Table 1). We need to collaborate with a research lab that can test the concentration of these chemicals on the TP medium plates before and after the LMJ growth to get additional support that LMJ is removing PHB, PAH and other hydrocarbons in the TP medium plates.

In summary, LMJ shares morphological and many biochemical traits with *Acidovorax* sp. Currently in the NCBI database, there are 60 genome assemblies of 17 characterized *Acidovorax* species and 67 genome assemblies of uncharacterized *Acidovorax* environmental isolates. The genome of the nearest relative of LMJ, *Acidovorax* sp. strain A16OP12, an environmental isolate, has not been sequenced yet. Because of funding limitations, we could not sequence the whole genome of LMJ at the time of this study. But we will have funds in fall 2020 to sequence the LMJ’s genome using Pacific Biosciences technology. This will allow us to: 1) determine assign a specific scientific genus/species/strain name to LMJ and, 2) will identify the genes in LMJ that play a role in its metabolic diversity relevant to bioremediation of common environmental pollutants.

## Data availability

### Underlying data

NCBI GenBank: Bacterium strain clone LIB091_C05_1243 variant 16S ribosomal RNA gene, partial sequence. Accession number MN633292.1.

Figshare: Antibiotic sensitivity data for the bacterial strain LMJ (Bacterium strain clone LIB091_C05_1243 variant 16S ribosomal RNA; GenBank Accession # MN633292.1). https://doi.org/10.6084/m9.figshare.12407735.v1 ^43^.

This project contains the following underlying data:

- Data S1 LMJ (XLSX). (Means and standard deviations of the zones of inhibitions of the bacterial strain LMJ and *Chlamydomonas*, induced by 4 antibiotics.)
- Data S2 LMJ (XLSX). (Statistical analyses of the zones of inhibitions of the bacterial strain LMJ and *Chlamydomonas*, induced by 4 antibiotics.)

Figshare: Images of antibiotic plates of the bacterial strain LMJ (Bacterium strain clone LIB091_C05_1243 variant 16S ribosomal RNA; GenBank Accession # MN633292.1) and green micro-alga Chlamydomonas from the antibiotic susceptibility disc diffusion tests. https://doi.org/10.6084/m9.figshare.12407741 ^44^.

This project contains 16 images of antibiotic plates used for the antibiotic susceptibility tests using the disc diffusion method for *Chlamydomonas* and the bacterial strain, LMJ.

Figshare: Tests using Tris-Phosphate medium (TP) to see if hydrocarbons, aromatic compounds and polyhydroxyalkanoates can be used by the bacterium LMJ (Bacterium strain clone LIB091_C05_1243 variant 16S ribosomal RNA; GenBank Accession # MN633292.1) as the sole carbon source. https://doi.org/10.6084/m9.figshare.12407822 ^40^.

This project contains 23 images of Tris-Phosphate medium plates containing different alternative carbon sources. Bacterium LMJ was streaked on these chemical plates to test if LMJ can utilize these chemicals as the sole carbon source for energy and growth.

Figshare: 16S rRNA partial gene sequences of the bacterial strain LMJ (Bacterium strain clone LIB091_C05_1243 variant 16S ribosomal RNA; GenBank Accession # MN633292.1). https://doi.org/10.6084/m9.figshare.12410372 ^49^.

This project contains the following underlying data:

- ABI extension files obtained from partial sequencing of the LMJ strain 16S rRNA.
- Corresponding text files of DNA sequences.

Figshare: Growth of bacterial strain LMJ (Bacterium strain clone LIB091_C05_1243 variant 16S ribosomal RNA; GenBank Accession # MN633292.1) and Staphylococcus aureus on Tryptic Soy Agar medium plates containing 5% sheep blood. https://doi.org/10.6084/m9.figshare.12420884 ^59^.

This project contains five images of strain LMJ and *Staphylococcus aureus* grown on Tryptic Soy Agar medium plates containing 5% sheep blood.

Figshare: TAP and LB liquid cultures of the bacterial strain LMJ (Bacterium strain clone LIB091_C05_1243 variant 16S ribosomal RNA; GenBank Accession # MN633292.1) grown for 4 days and Gram stains of LMJ from these liquid cultures. Growth of LMJ on TAP-agar from a 96 hours-grown TAP liquid culture is also shown. https://doi.org/10.6084/m9.figshare.12420893 ^50^.

This project contains images of bacterial strain LMJ grown in liquid TAP and liquid LB medium for 96 hours, taken at 24-h intervals, alongside images of Gram stains for strain LMJ grown in each medium.

Data are available under the terms of the Creative Commons Attribution 4.0 International license (CC-BY 4.0).
