# Supplementary figures and images for: Isolation and characterization of a novel bacterial strain from a Tris-Acetate-Phosphate agar medium plate of the green micro-alga Chlamydomonas reinhardtii that can utilize common environmental pollutants as a carbon source
Source: F1000Res. 2020 Jun 29;9:656. [Version 1] doi: 10.12688/f1000research.24680.1 (PMC7425125; doi:10.12688/f1000research.24680.1)

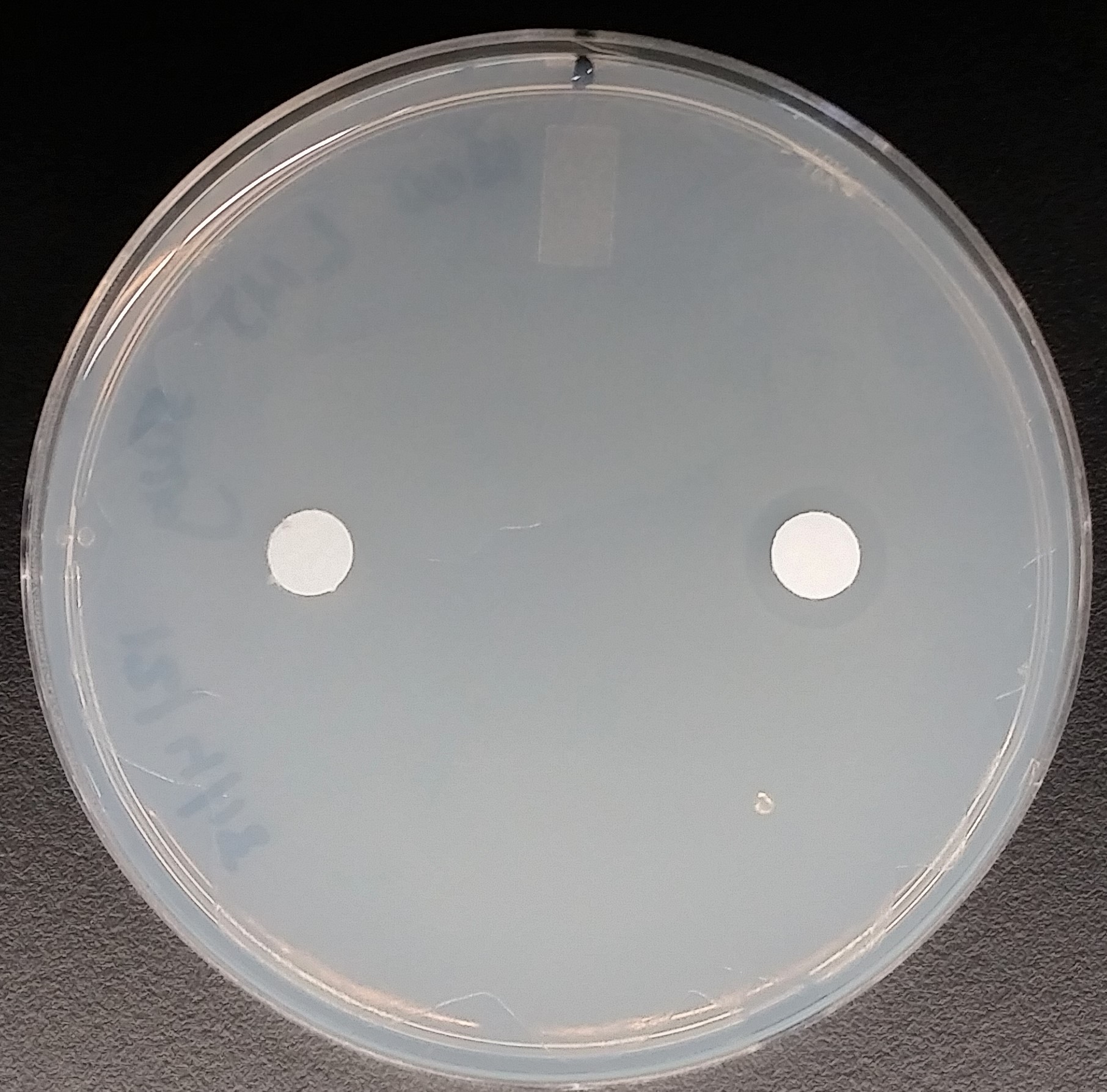

Supplement: Images of antibiotic plates of the bacterial strain LMJ (Bacterium strain clone LIB091_C05_1243 variant 16S ribosomal RNA; GenBank Accession # MN633292.1) and green micro-alga Chlamydomonas from the antibiotic susceptibility disc diffusion tests. — This file contains 16 images of antibiotic plates used for the antibiotic susceptibility tests using the disc diffusion method for Chlamydomonas and the bacterial strain, LMJ. Antibiotics tested are: penicillin, chloramphenicol, polymyxin B and neomycin. Two different doses of antibiotics were used: 50 and 100 micrograms of each antibiotics. On the LMJ antibiotic plates, the filter paper disc on the right contains the antibiotic and that on the left contains sterile water (control). On the Chlamydomonas antibiotic plates, the filter paper disc on the left contains the antibiotic and that on the right contains sterile water (control). [file f1000research-9-27224-s0001.tgz › 20181207_111426Neo72hrs50ugcropped.jpg]

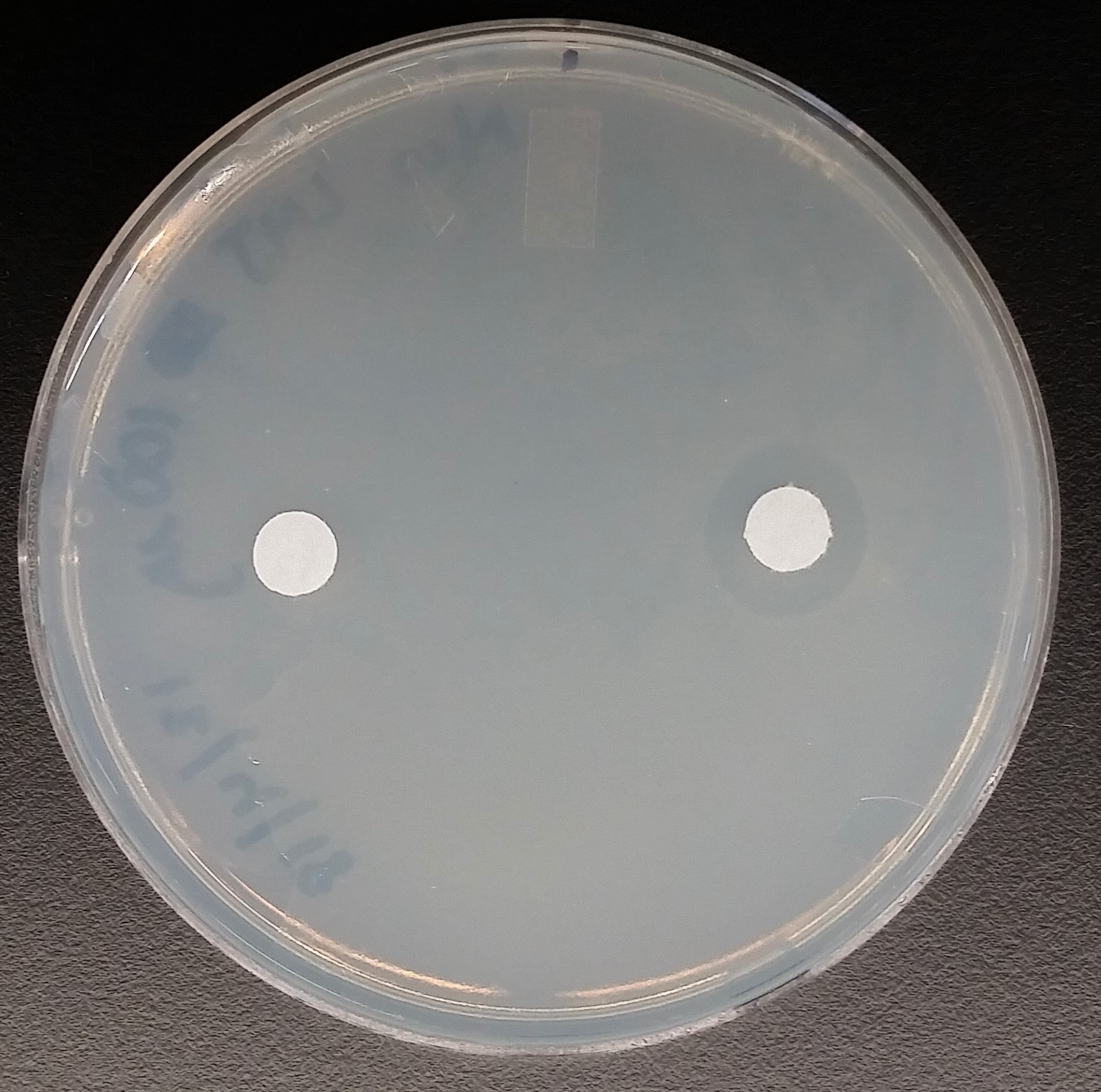

Supplement: Images of antibiotic plates of the bacterial strain LMJ (Bacterium strain clone LIB091_C05_1243 variant 16S ribosomal RNA; GenBank Accession # MN633292.1) and green micro-alga Chlamydomonas from the antibiotic susceptibility disc diffusion tests. — This file contains 16 images of antibiotic plates used for the antibiotic susceptibility tests using the disc diffusion method for Chlamydomonas and the bacterial strain, LMJ. Antibiotics tested are: penicillin, chloramphenicol, polymyxin B and neomycin. Two different doses of antibiotics were used: 50 and 100 micrograms of each antibiotics. On the LMJ antibiotic plates, the filter paper disc on the right contains the antibiotic and that on the left contains sterile water (control). On the Chlamydomonas antibiotic plates, the filter paper disc on the left contains the antibiotic and that on the right contains sterile water (control). [file f1000research-9-27224-s0001.tgz › 20181207_111508Neo72hrs100ugcropped.jpg]

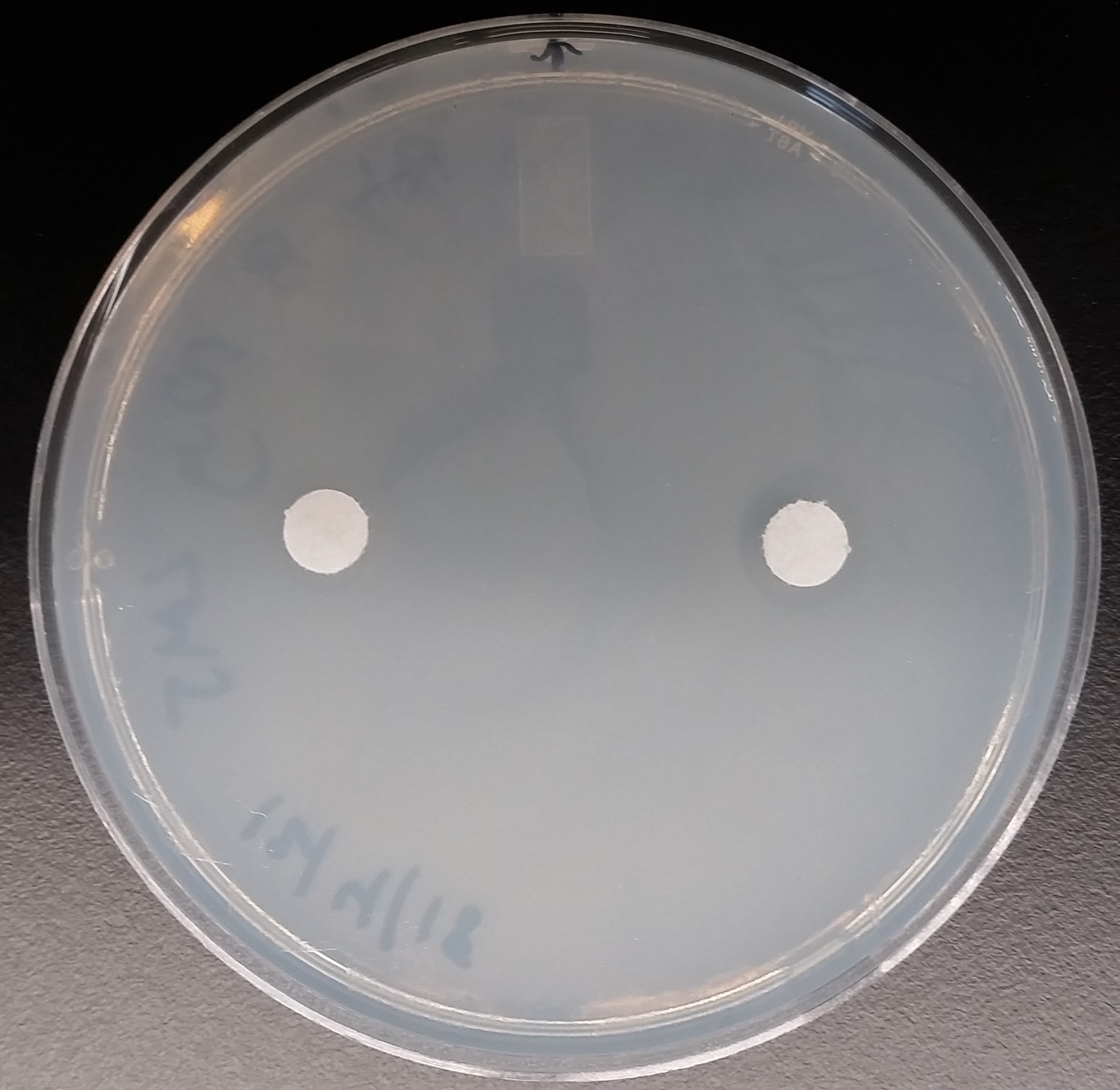

Supplement: Images of antibiotic plates of the bacterial strain LMJ (Bacterium strain clone LIB091_C05_1243 variant 16S ribosomal RNA; GenBank Accession # MN633292.1) and green micro-alga Chlamydomonas from the antibiotic susceptibility disc diffusion tests. — This file contains 16 images of antibiotic plates used for the antibiotic susceptibility tests using the disc diffusion method for Chlamydomonas and the bacterial strain, LMJ. Antibiotics tested are: penicillin, chloramphenicol, polymyxin B and neomycin. Two different doses of antibiotics were used: 50 and 100 micrograms of each antibiotics. On the LMJ antibiotic plates, the filter paper disc on the right contains the antibiotic and that on the left contains sterile water (control). On the Chlamydomonas antibiotic plates, the filter paper disc on the left contains the antibiotic and that on the right contains sterile water (control). [file f1000research-9-27224-s0001.tgz › 20181207_111729PolyB72hrs50ugcropped.jpg]

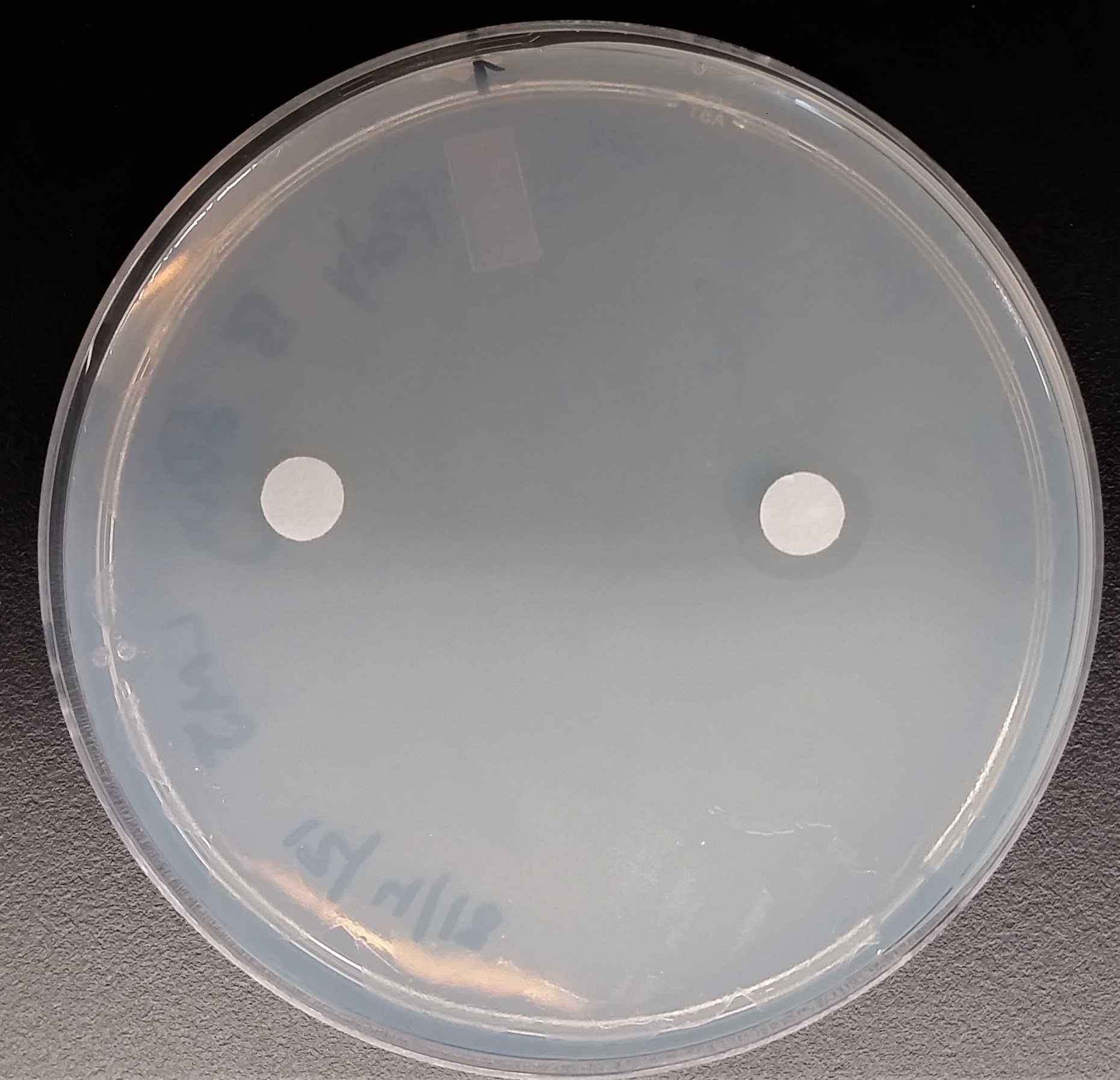

Supplement: Images of antibiotic plates of the bacterial strain LMJ (Bacterium strain clone LIB091_C05_1243 variant 16S ribosomal RNA; GenBank Accession # MN633292.1) and green micro-alga Chlamydomonas from the antibiotic susceptibility disc diffusion tests. — This file contains 16 images of antibiotic plates used for the antibiotic susceptibility tests using the disc diffusion method for Chlamydomonas and the bacterial strain, LMJ. Antibiotics tested are: penicillin, chloramphenicol, polymyxin B and neomycin. Two different doses of antibiotics were used: 50 and 100 micrograms of each antibiotics. On the LMJ antibiotic plates, the filter paper disc on the right contains the antibiotic and that on the left contains sterile water (control). On the Chlamydomonas antibiotic plates, the filter paper disc on the left contains the antibiotic and that on the right contains sterile water (control). [file f1000research-9-27224-s0001.tgz › 20181207_111830PolyB72hrs100ugcropped.jpg]

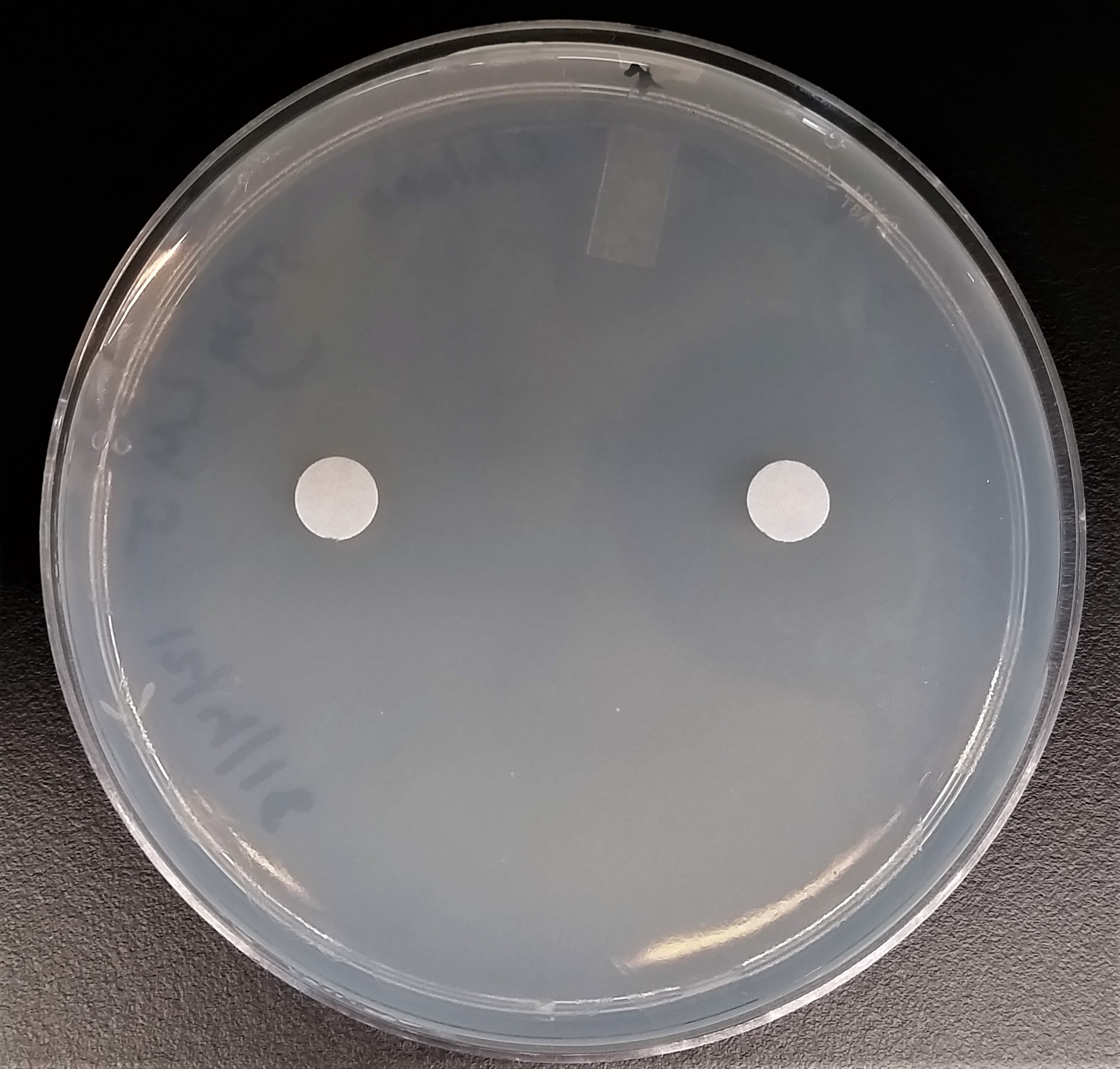

Supplement: Images of antibiotic plates of the bacterial strain LMJ (Bacterium strain clone LIB091_C05_1243 variant 16S ribosomal RNA; GenBank Accession # MN633292.1) and green micro-alga Chlamydomonas from the antibiotic susceptibility disc diffusion tests. — This file contains 16 images of antibiotic plates used for the antibiotic susceptibility tests using the disc diffusion method for Chlamydomonas and the bacterial strain, LMJ. Antibiotics tested are: penicillin, chloramphenicol, polymyxin B and neomycin. Two different doses of antibiotics were used: 50 and 100 micrograms of each antibiotics. On the LMJ antibiotic plates, the filter paper disc on the right contains the antibiotic and that on the left contains sterile water (control). On the Chlamydomonas antibiotic plates, the filter paper disc on the left contains the antibiotic and that on the right contains sterile water (control). [file f1000research-9-27224-s0001.tgz › 20181207_112357Chloro72hrs50ugcropped.jpg]

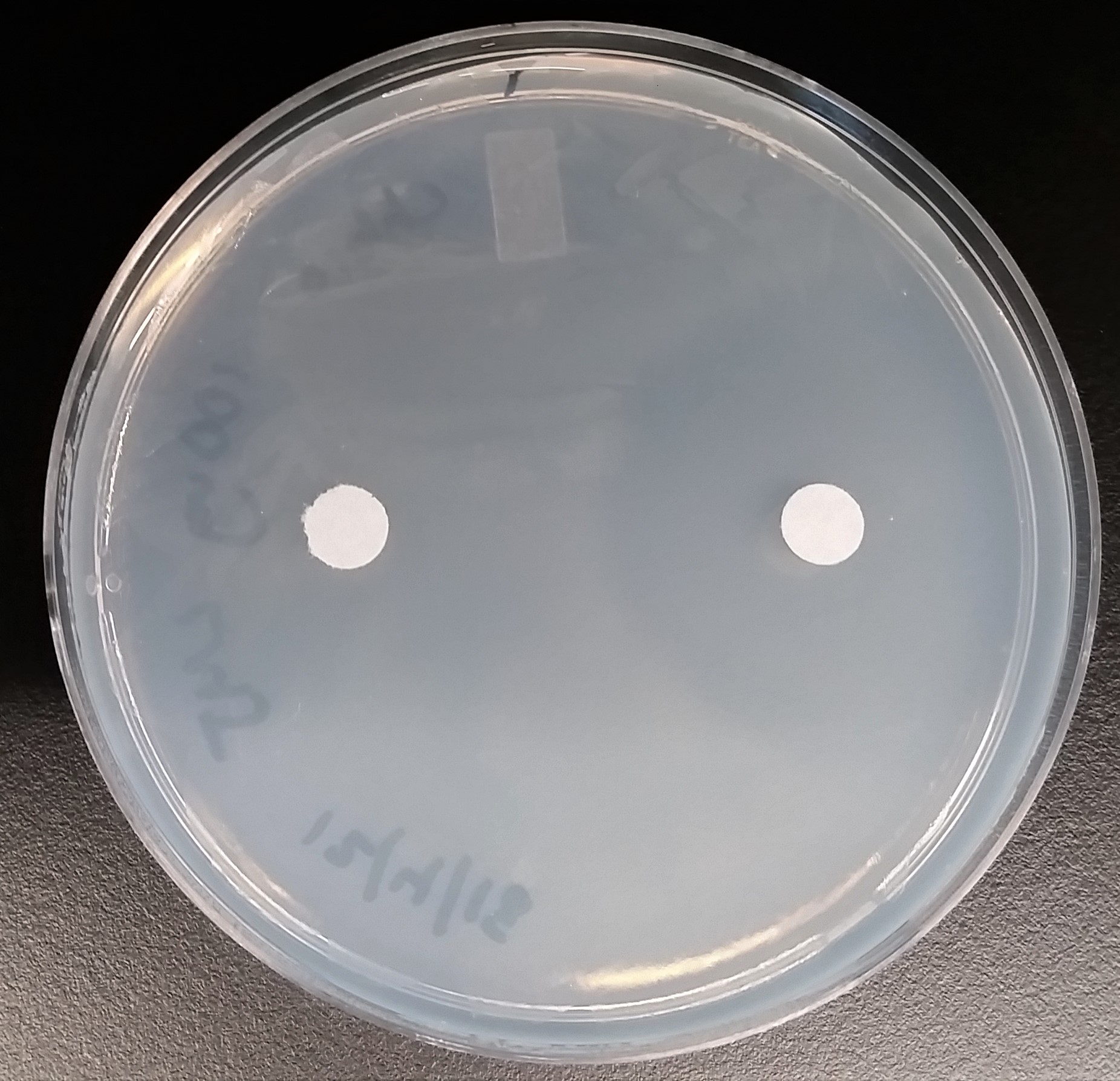

Supplement: Images of antibiotic plates of the bacterial strain LMJ (Bacterium strain clone LIB091_C05_1243 variant 16S ribosomal RNA; GenBank Accession # MN633292.1) and green micro-alga Chlamydomonas from the antibiotic susceptibility disc diffusion tests. — This file contains 16 images of antibiotic plates used for the antibiotic susceptibility tests using the disc diffusion method for Chlamydomonas and the bacterial strain, LMJ. Antibiotics tested are: penicillin, chloramphenicol, polymyxin B and neomycin. Two different doses of antibiotics were used: 50 and 100 micrograms of each antibiotics. On the LMJ antibiotic plates, the filter paper disc on the right contains the antibiotic and that on the left contains sterile water (control). On the Chlamydomonas antibiotic plates, the filter paper disc on the left contains the antibiotic and that on the right contains sterile water (control). [file f1000research-9-27224-s0001.tgz › 20181207_112500Chloro72hrs100ugcropped.jpg]

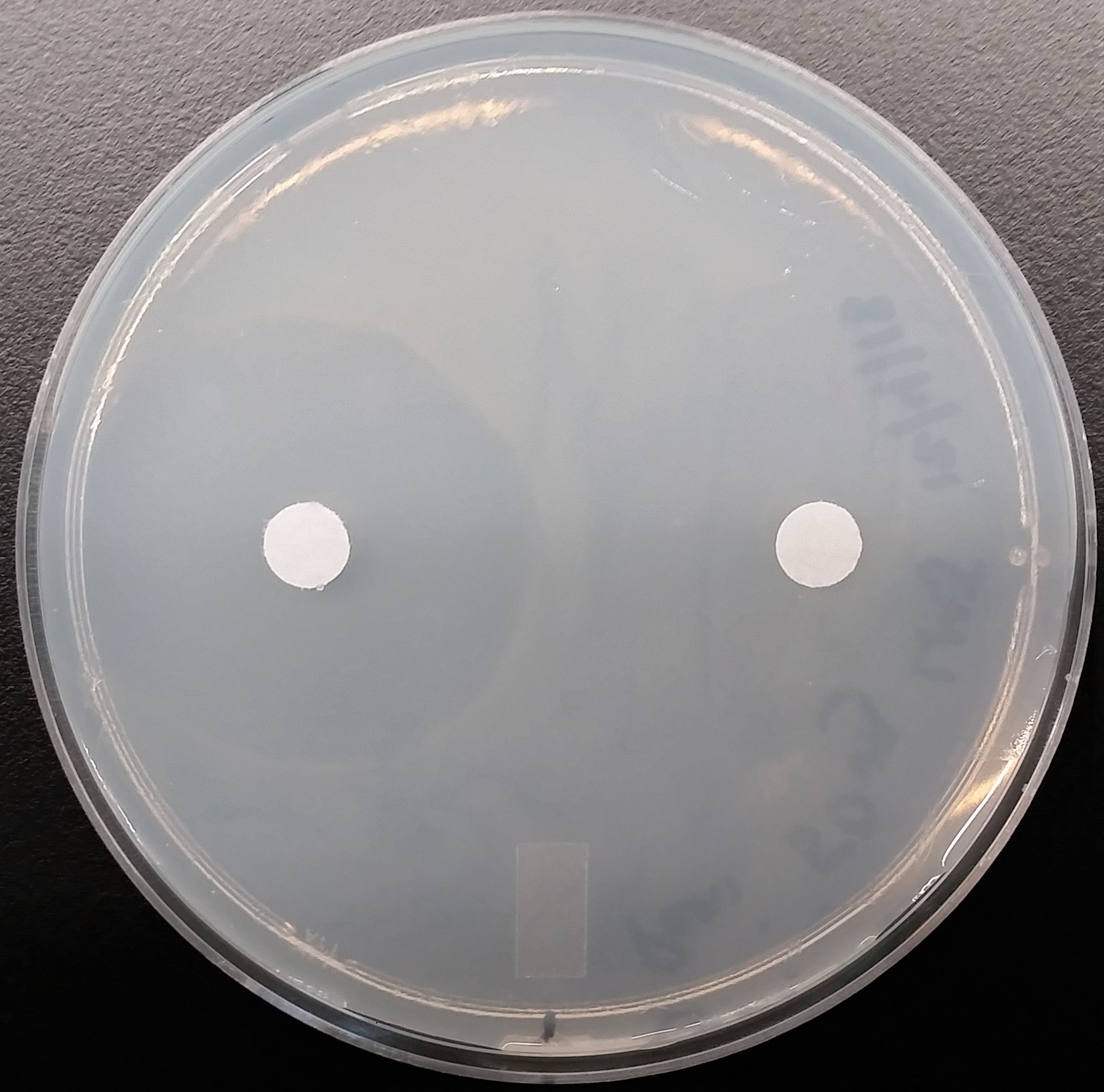

Supplement: Images of antibiotic plates of the bacterial strain LMJ (Bacterium strain clone LIB091_C05_1243 variant 16S ribosomal RNA; GenBank Accession # MN633292.1) and green micro-alga Chlamydomonas from the antibiotic susceptibility disc diffusion tests. — This file contains 16 images of antibiotic plates used for the antibiotic susceptibility tests using the disc diffusion method for Chlamydomonas and the bacterial strain, LMJ. Antibiotics tested are: penicillin, chloramphenicol, polymyxin B and neomycin. Two different doses of antibiotics were used: 50 and 100 micrograms of each antibiotics. On the LMJ antibiotic plates, the filter paper disc on the right contains the antibiotic and that on the left contains sterile water (control). On the Chlamydomonas antibiotic plates, the filter paper disc on the left contains the antibiotic and that on the right contains sterile water (control). [file f1000research-9-27224-s0001.tgz › 20181207_112647Penicillin72hrs50ug.jpg]

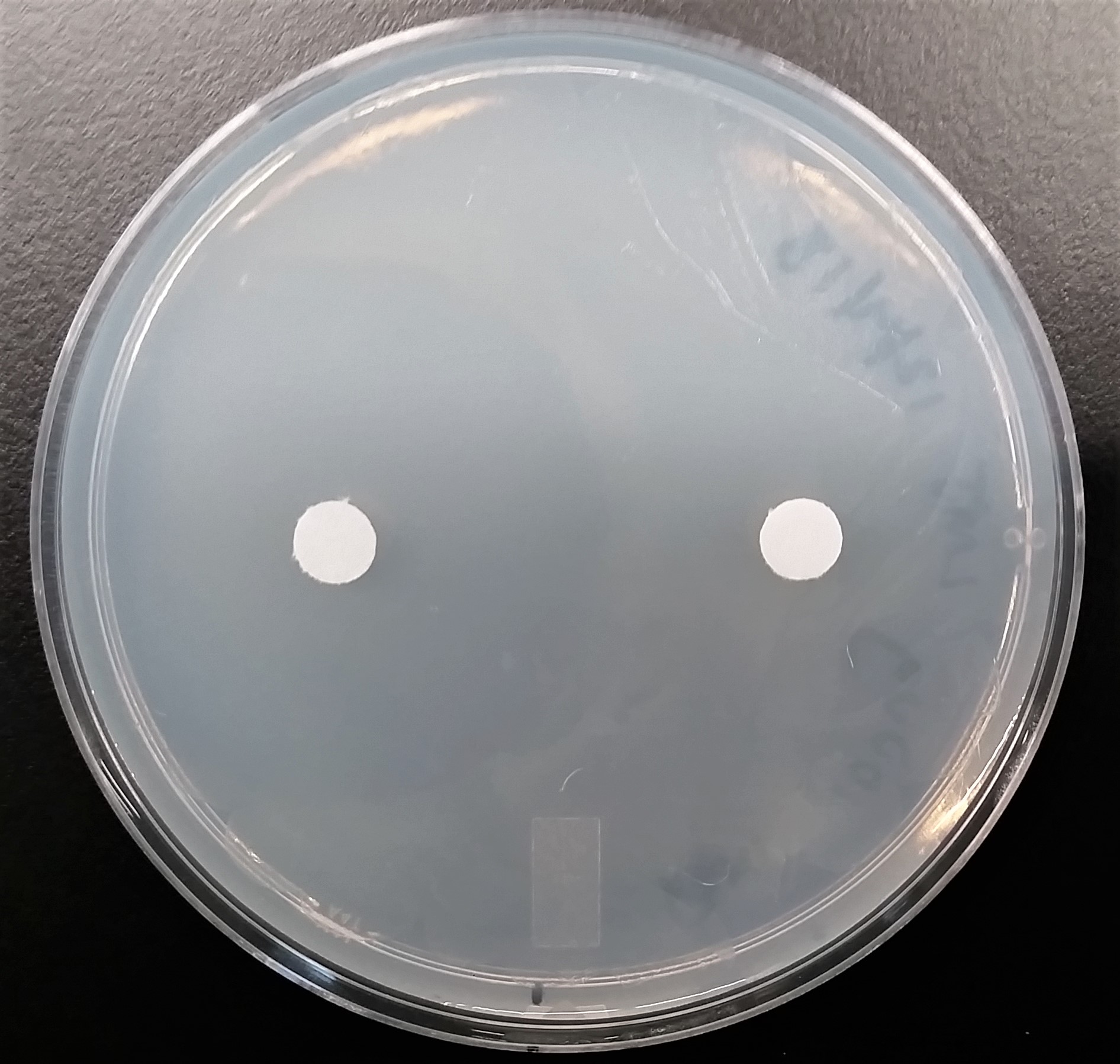

Supplement: Images of antibiotic plates of the bacterial strain LMJ (Bacterium strain clone LIB091_C05_1243 variant 16S ribosomal RNA; GenBank Accession # MN633292.1) and green micro-alga Chlamydomonas from the antibiotic susceptibility disc diffusion tests. — This file contains 16 images of antibiotic plates used for the antibiotic susceptibility tests using the disc diffusion method for Chlamydomonas and the bacterial strain, LMJ. Antibiotics tested are: penicillin, chloramphenicol, polymyxin B and neomycin. Two different doses of antibiotics were used: 50 and 100 micrograms of each antibiotics. On the LMJ antibiotic plates, the filter paper disc on the right contains the antibiotic and that on the left contains sterile water (control). On the Chlamydomonas antibiotic plates, the filter paper disc on the left contains the antibiotic and that on the right contains sterile water (control). [file f1000research-9-27224-s0001.tgz › 20181207_112859Penicillin72hrs100ug.jpg]

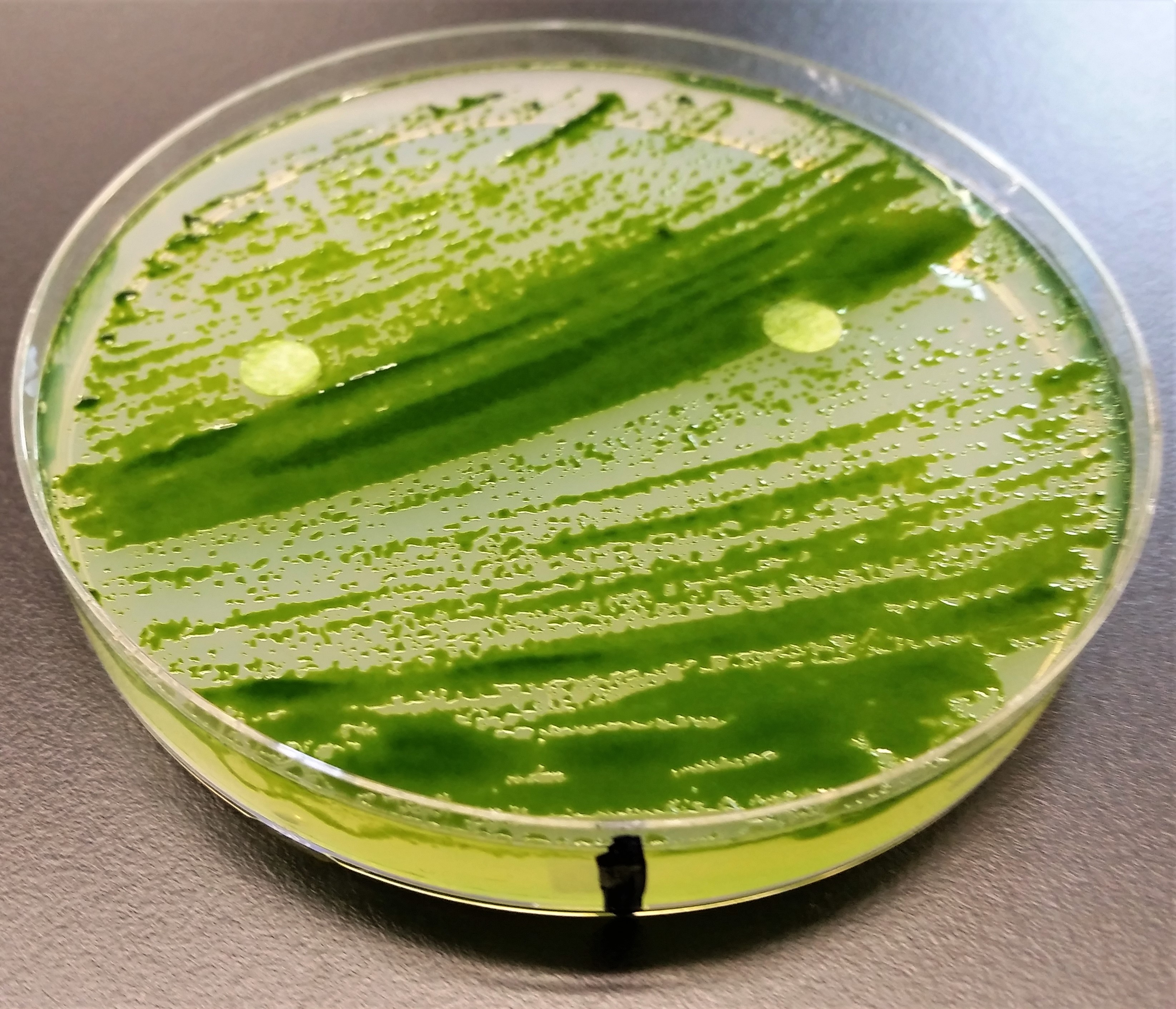

Supplement: Images of antibiotic plates of the bacterial strain LMJ (Bacterium strain clone LIB091_C05_1243 variant 16S ribosomal RNA; GenBank Accession # MN633292.1) and green micro-alga Chlamydomonas from the antibiotic susceptibility disc diffusion tests. — This file contains 16 images of antibiotic plates used for the antibiotic susceptibility tests using the disc diffusion method for Chlamydomonas and the bacterial strain, LMJ. Antibiotics tested are: penicillin, chloramphenicol, polymyxin B and neomycin. Two different doses of antibiotics were used: 50 and 100 micrograms of each antibiotics. On the LMJ antibiotic plates, the filter paper disc on the right contains the antibiotic and that on the left contains sterile water (control). On the Chlamydomonas antibiotic plates, the filter paper disc on the left contains the antibiotic and that on the right contains sterile water (control). [file f1000research-9-27224-s0001.tgz › 50microChloroChlamycropped.jpg]

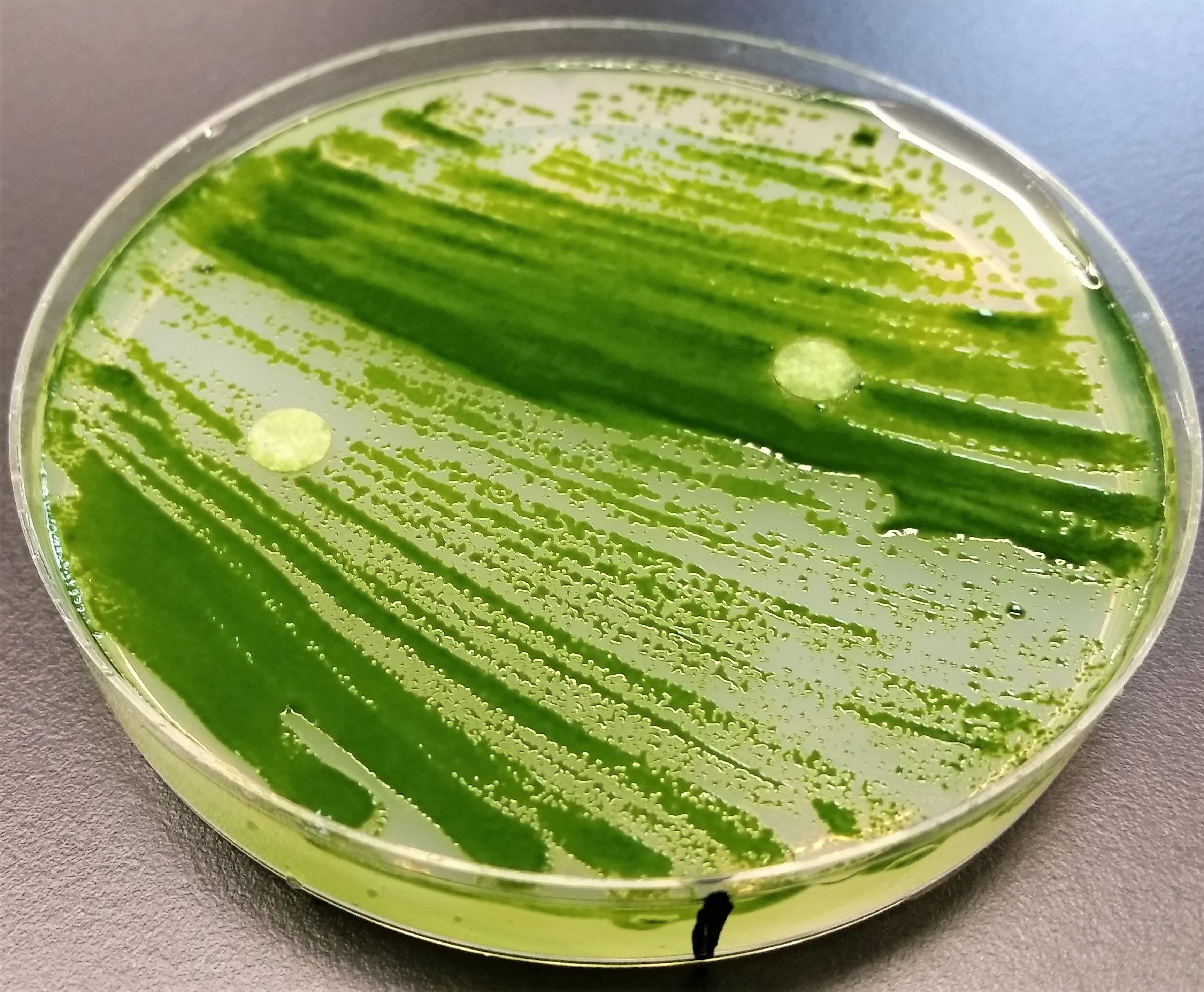

Supplement: Images of antibiotic plates of the bacterial strain LMJ (Bacterium strain clone LIB091_C05_1243 variant 16S ribosomal RNA; GenBank Accession # MN633292.1) and green micro-alga Chlamydomonas from the antibiotic susceptibility disc diffusion tests. — This file contains 16 images of antibiotic plates used for the antibiotic susceptibility tests using the disc diffusion method for Chlamydomonas and the bacterial strain, LMJ. Antibiotics tested are: penicillin, chloramphenicol, polymyxin B and neomycin. Two different doses of antibiotics were used: 50 and 100 micrograms of each antibiotics. On the LMJ antibiotic plates, the filter paper disc on the right contains the antibiotic and that on the left contains sterile water (control). On the Chlamydomonas antibiotic plates, the filter paper disc on the left contains the antibiotic and that on the right contains sterile water (control). [file f1000research-9-27224-s0001.tgz › 100microchlorocropped.jpg]

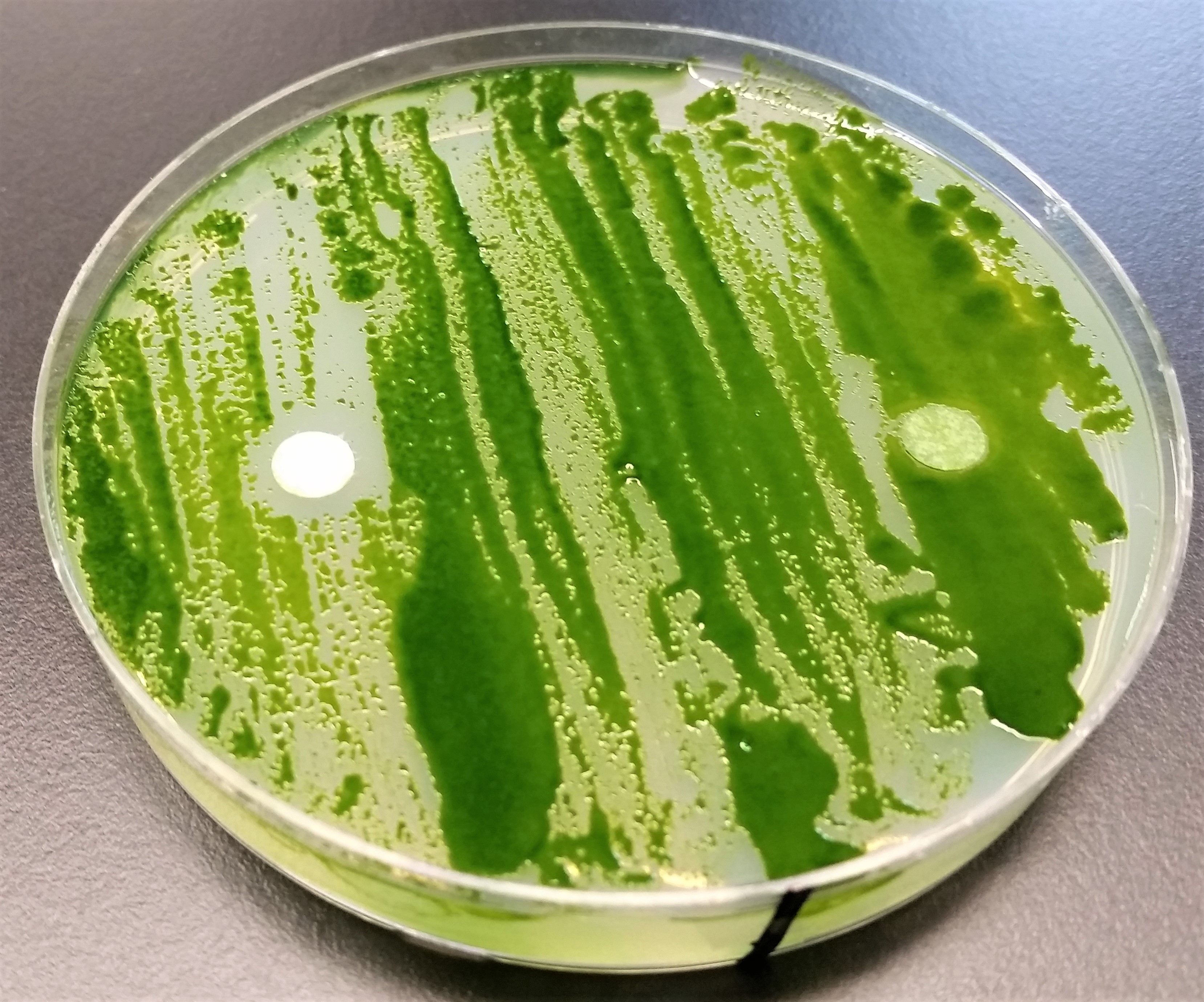

Supplement: Images of antibiotic plates of the bacterial strain LMJ (Bacterium strain clone LIB091_C05_1243 variant 16S ribosomal RNA; GenBank Accession # MN633292.1) and green micro-alga Chlamydomonas from the antibiotic susceptibility disc diffusion tests. — This file contains 16 images of antibiotic plates used for the antibiotic susceptibility tests using the disc diffusion method for Chlamydomonas and the bacterial strain, LMJ. Antibiotics tested are: penicillin, chloramphenicol, polymyxin B and neomycin. Two different doses of antibiotics were used: 50 and 100 micrograms of each antibiotics. On the LMJ antibiotic plates, the filter paper disc on the right contains the antibiotic and that on the left contains sterile water (control). On the Chlamydomonas antibiotic plates, the filter paper disc on the left contains the antibiotic and that on the right contains sterile water (control). [file f1000research-9-27224-s0001.tgz › 50microNeoChlamycropped.jpg]

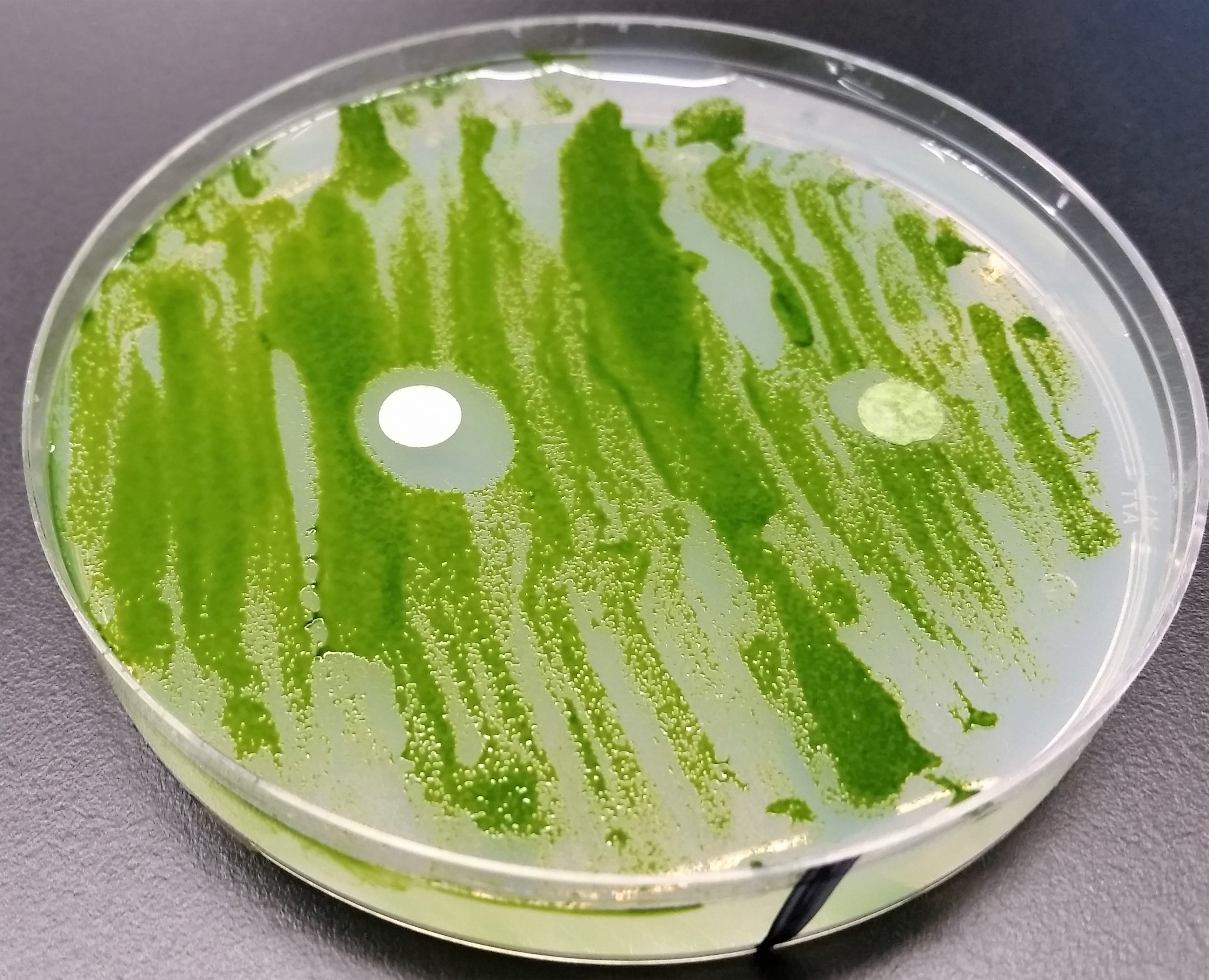

Supplement: Images of antibiotic plates of the bacterial strain LMJ (Bacterium strain clone LIB091_C05_1243 variant 16S ribosomal RNA; GenBank Accession # MN633292.1) and green micro-alga Chlamydomonas from the antibiotic susceptibility disc diffusion tests. — This file contains 16 images of antibiotic plates used for the antibiotic susceptibility tests using the disc diffusion method for Chlamydomonas and the bacterial strain, LMJ. Antibiotics tested are: penicillin, chloramphenicol, polymyxin B and neomycin. Two different doses of antibiotics were used: 50 and 100 micrograms of each antibiotics. On the LMJ antibiotic plates, the filter paper disc on the right contains the antibiotic and that on the left contains sterile water (control). On the Chlamydomonas antibiotic plates, the filter paper disc on the left contains the antibiotic and that on the right contains sterile water (control). [file f1000research-9-27224-s0001.tgz › 100microNeocropped.jpg]

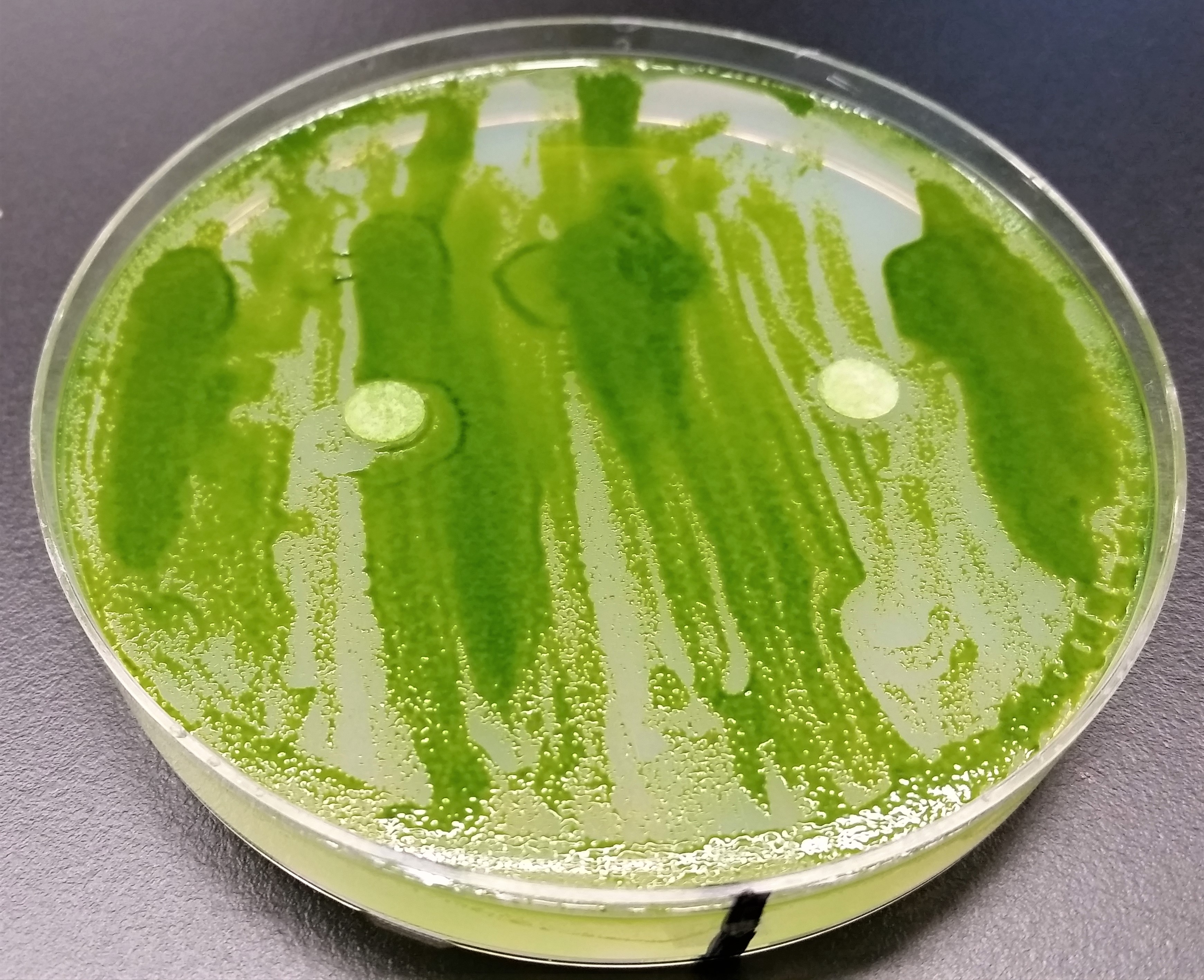

Supplement: Images of antibiotic plates of the bacterial strain LMJ (Bacterium strain clone LIB091_C05_1243 variant 16S ribosomal RNA; GenBank Accession # MN633292.1) and green micro-alga Chlamydomonas from the antibiotic susceptibility disc diffusion tests. — This file contains 16 images of antibiotic plates used for the antibiotic susceptibility tests using the disc diffusion method for Chlamydomonas and the bacterial strain, LMJ. Antibiotics tested are: penicillin, chloramphenicol, polymyxin B and neomycin. Two different doses of antibiotics were used: 50 and 100 micrograms of each antibiotics. On the LMJ antibiotic plates, the filter paper disc on the right contains the antibiotic and that on the left contains sterile water (control). On the Chlamydomonas antibiotic plates, the filter paper disc on the left contains the antibiotic and that on the right contains sterile water (control). [file f1000research-9-27224-s0001.tgz › 50microPeniChlamycropped.jpg]

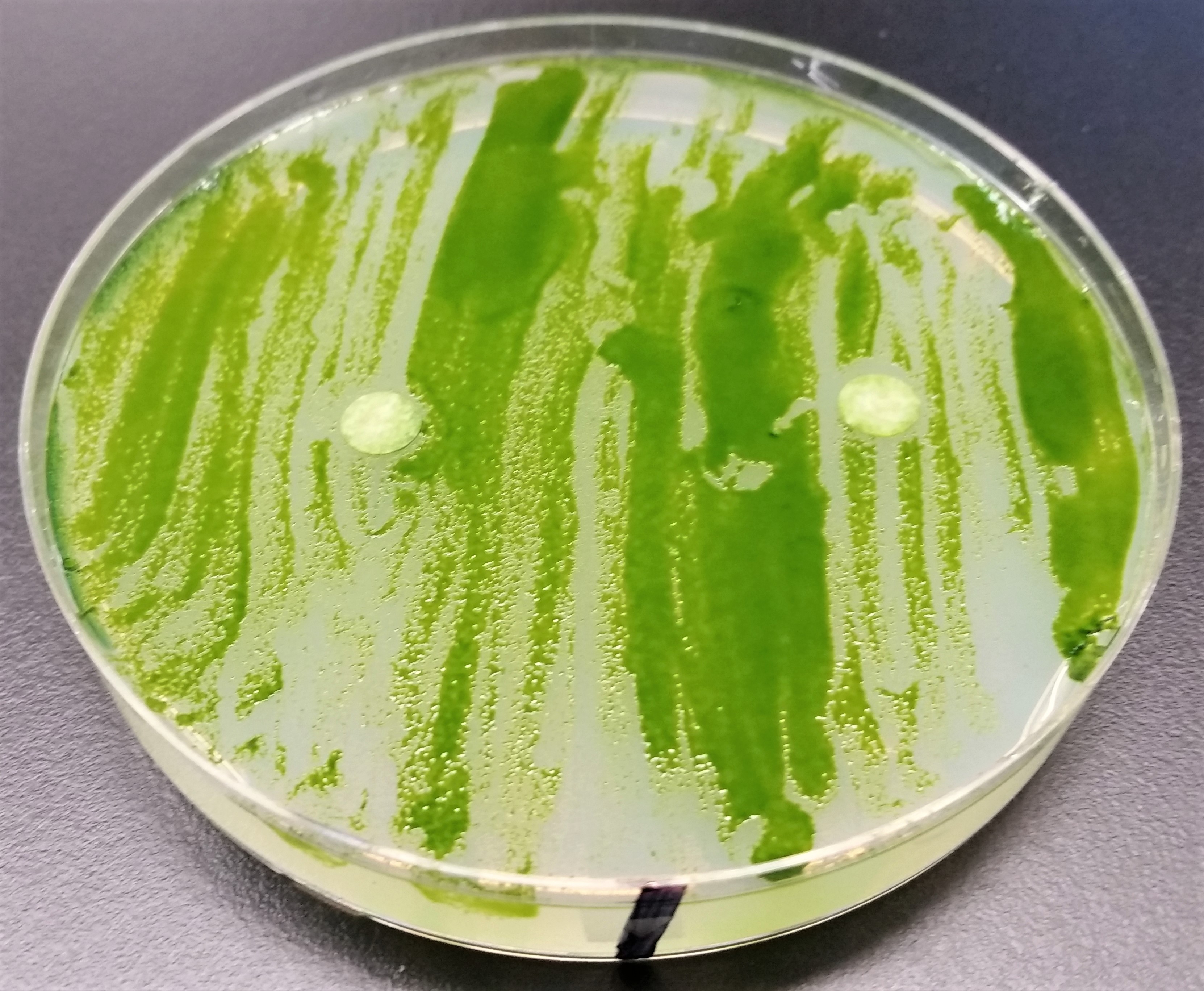

Supplement: Images of antibiotic plates of the bacterial strain LMJ (Bacterium strain clone LIB091_C05_1243 variant 16S ribosomal RNA; GenBank Accession # MN633292.1) and green micro-alga Chlamydomonas from the antibiotic susceptibility disc diffusion tests. — This file contains 16 images of antibiotic plates used for the antibiotic susceptibility tests using the disc diffusion method for Chlamydomonas and the bacterial strain, LMJ. Antibiotics tested are: penicillin, chloramphenicol, polymyxin B and neomycin. Two different doses of antibiotics were used: 50 and 100 micrograms of each antibiotics. On the LMJ antibiotic plates, the filter paper disc on the right contains the antibiotic and that on the left contains sterile water (control). On the Chlamydomonas antibiotic plates, the filter paper disc on the left contains the antibiotic and that on the right contains sterile water (control). [file f1000research-9-27224-s0001.tgz › 100microPenicropped.jpg]

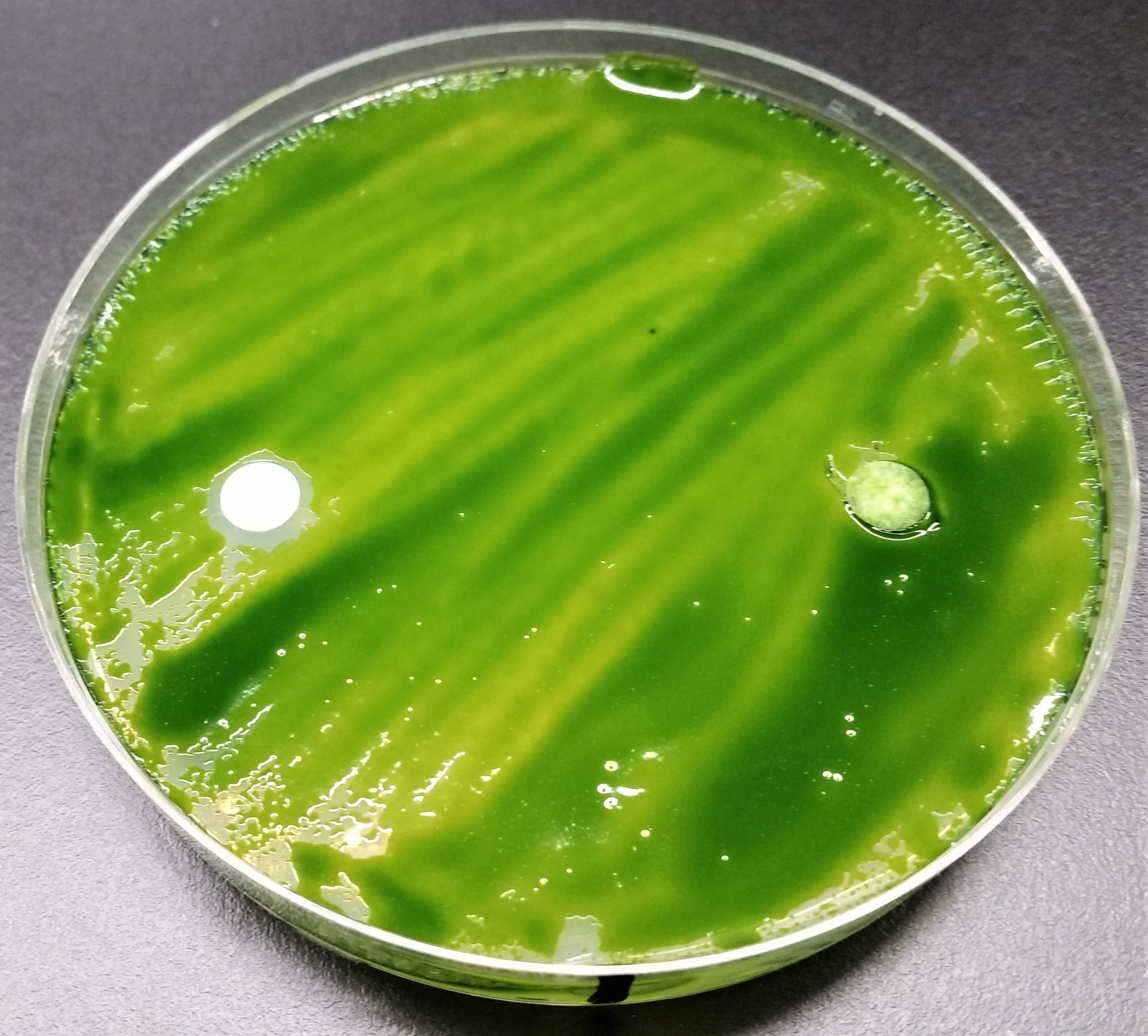

Supplement: Images of antibiotic plates of the bacterial strain LMJ (Bacterium strain clone LIB091_C05_1243 variant 16S ribosomal RNA; GenBank Accession # MN633292.1) and green micro-alga Chlamydomonas from the antibiotic susceptibility disc diffusion tests. — This file contains 16 images of antibiotic plates used for the antibiotic susceptibility tests using the disc diffusion method for Chlamydomonas and the bacterial strain, LMJ. Antibiotics tested are: penicillin, chloramphenicol, polymyxin B and neomycin. Two different doses of antibiotics were used: 50 and 100 micrograms of each antibiotics. On the LMJ antibiotic plates, the filter paper disc on the right contains the antibiotic and that on the left contains sterile water (control). On the Chlamydomonas antibiotic plates, the filter paper disc on the left contains the antibiotic and that on the right contains sterile water (control). [file f1000research-9-27224-s0001.tgz › 50micropolymyxinB2ndbatchcropped.jpg]

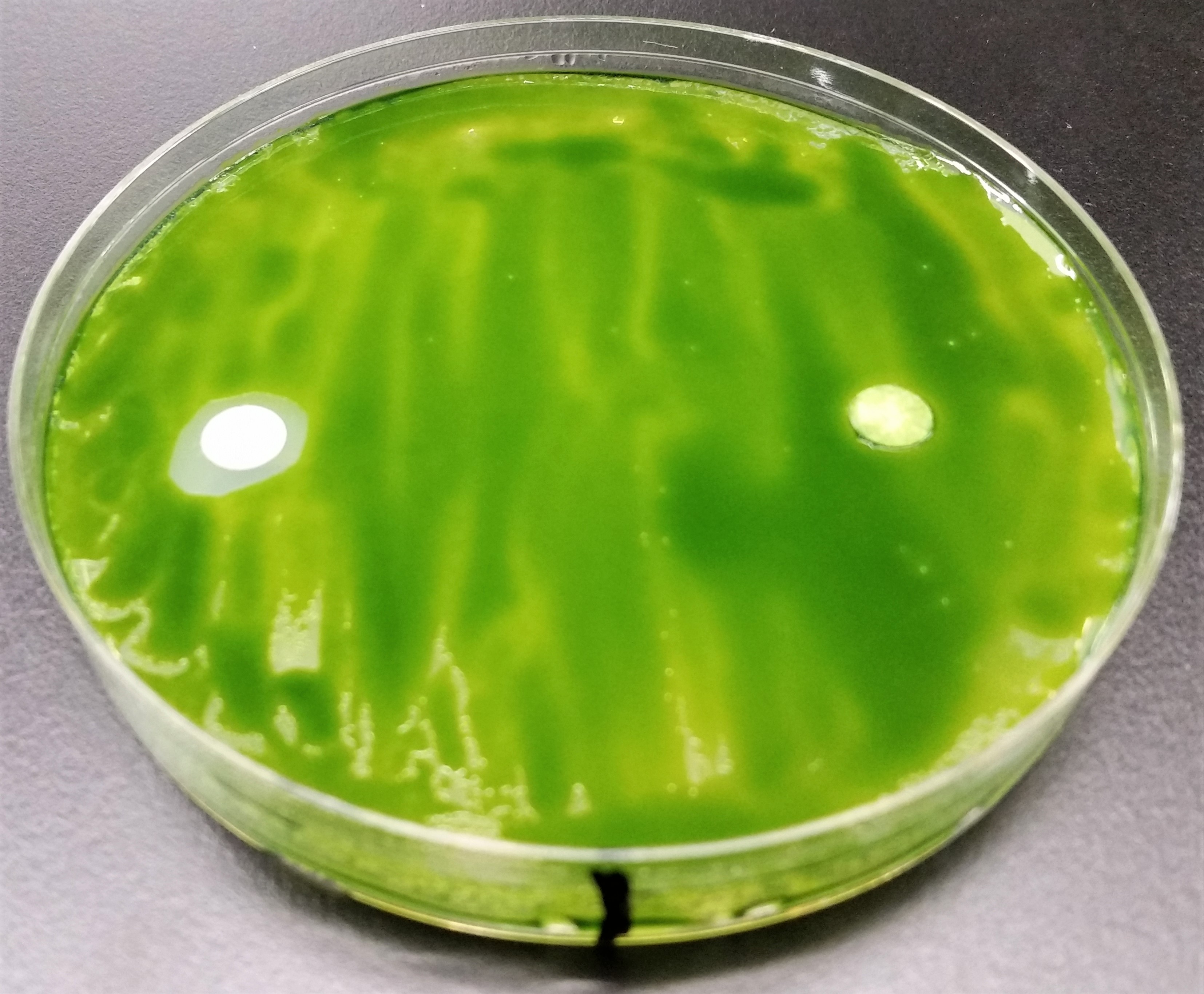

Supplement: Images of antibiotic plates of the bacterial strain LMJ (Bacterium strain clone LIB091_C05_1243 variant 16S ribosomal RNA; GenBank Accession # MN633292.1) and green micro-alga Chlamydomonas from the antibiotic susceptibility disc diffusion tests. — This file contains 16 images of antibiotic plates used for the antibiotic susceptibility tests using the disc diffusion method for Chlamydomonas and the bacterial strain, LMJ. Antibiotics tested are: penicillin, chloramphenicol, polymyxin B and neomycin. Two different doses of antibiotics were used: 50 and 100 micrograms of each antibiotics. On the LMJ antibiotic plates, the filter paper disc on the right contains the antibiotic and that on the left contains sterile water (control). On the Chlamydomonas antibiotic plates, the filter paper disc on the left contains the antibiotic and that on the right contains sterile water (control). [file f1000research-9-27224-s0001.tgz › 100micropolymyxin2ndbatchcropped.jpg]

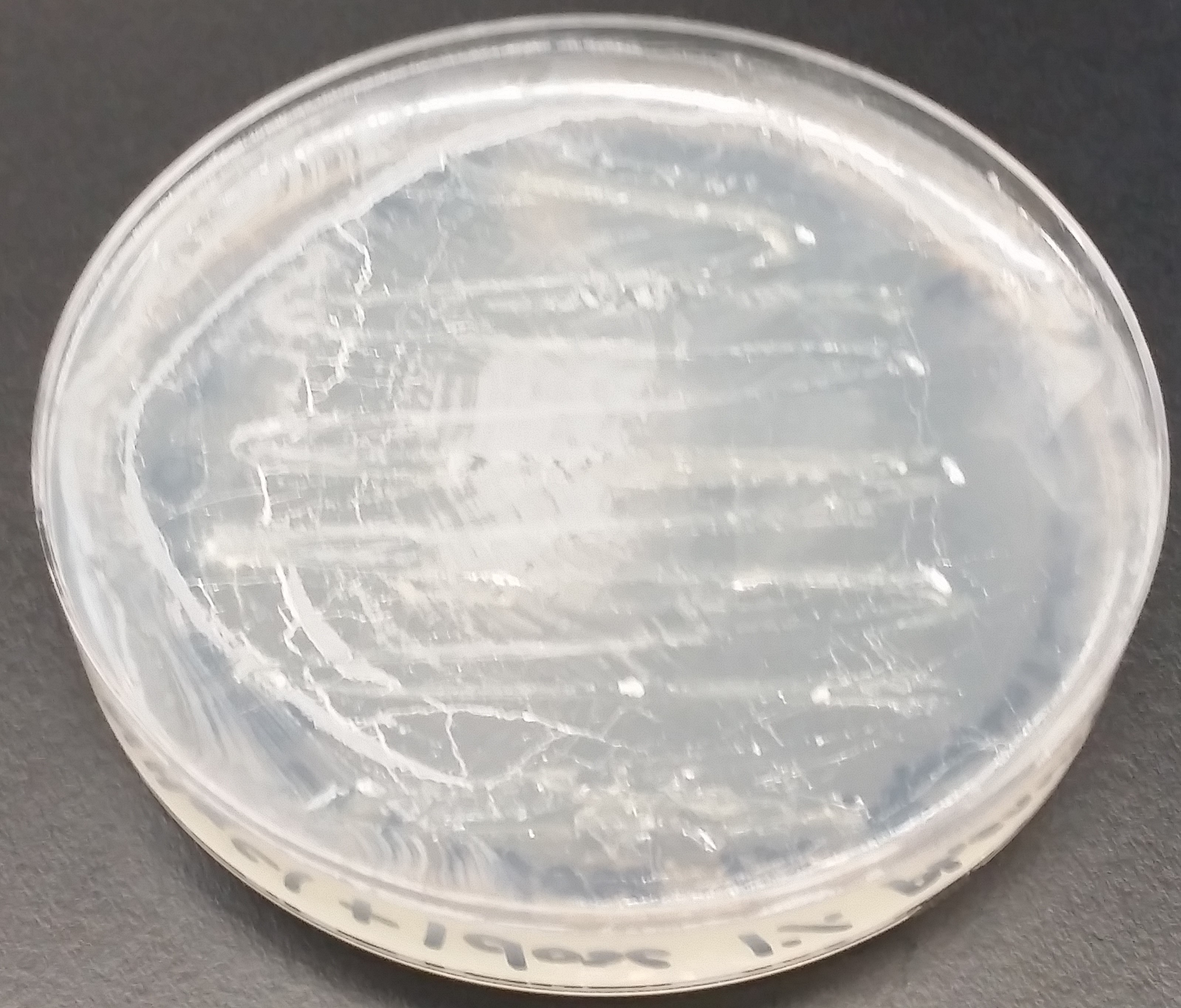

Supplement: Tests using Tris-Phosphate medium (TP) to see if hydrocarbons, aromatic compounds and polyhydroxyalkanoates can be used by the bacterium LMJ (Bacterium strain clone LIB091_C05_1243 variant 16S ribosomal RNA; GenBank Accession # MN633292.1) as the sole carbon source. — This file contains 23 images of TP (Tris-Phosphate) medium plates containing different alternative carbon sources. Bacterium LMJ was streaked on these chemical plates to test if LMJ can utilize these chemicals as the sole carbon source for energy and growth. 1% stocks of the following chemicals were tested: cyclohexyl chloride, phenanthrene, napthalene, benzoic acid, phenyl acetate. 2% (v/v) stocks of fresh and used car motor oil 10W30 were also tested. The doses used are given in mL in the file name. [file f1000research-9-27224-s0002.tgz › LMJphenanthrene2mL.jpg]

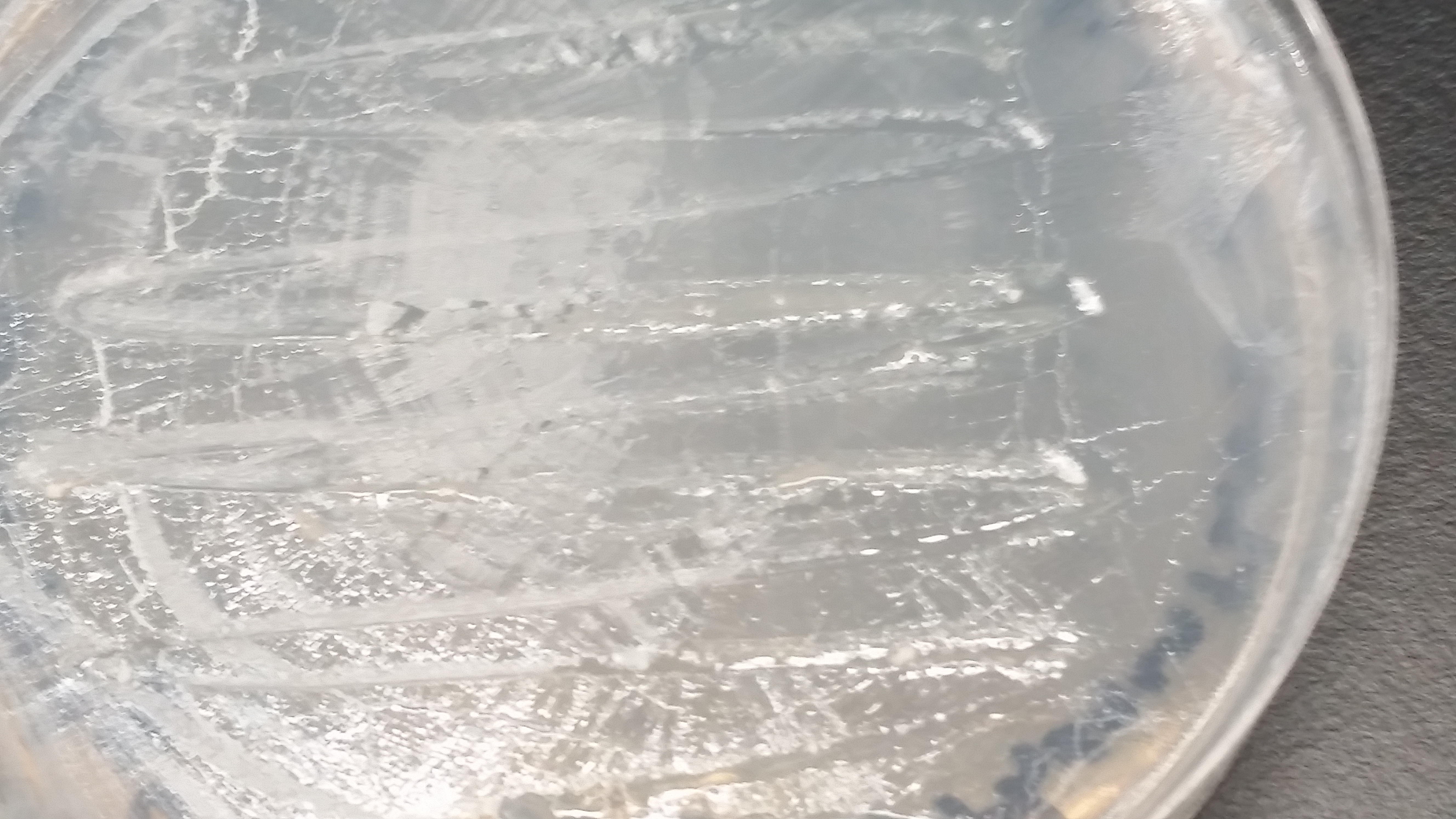

Supplement: Tests using Tris-Phosphate medium (TP) to see if hydrocarbons, aromatic compounds and polyhydroxyalkanoates can be used by the bacterium LMJ (Bacterium strain clone LIB091_C05_1243 variant 16S ribosomal RNA; GenBank Accession # MN633292.1) as the sole carbon source. — This file contains 23 images of TP (Tris-Phosphate) medium plates containing different alternative carbon sources. Bacterium LMJ was streaked on these chemical plates to test if LMJ can utilize these chemicals as the sole carbon source for energy and growth. 1% stocks of the following chemicals were tested: cyclohexyl chloride, phenanthrene, napthalene, benzoic acid, phenyl acetate. 2% (v/v) stocks of fresh and used car motor oil 10W30 were also tested. The doses used are given in mL in the file name. [file f1000research-9-27224-s0002.tgz › LMJLMJphenanthrenezoomup2mLB.jpg]

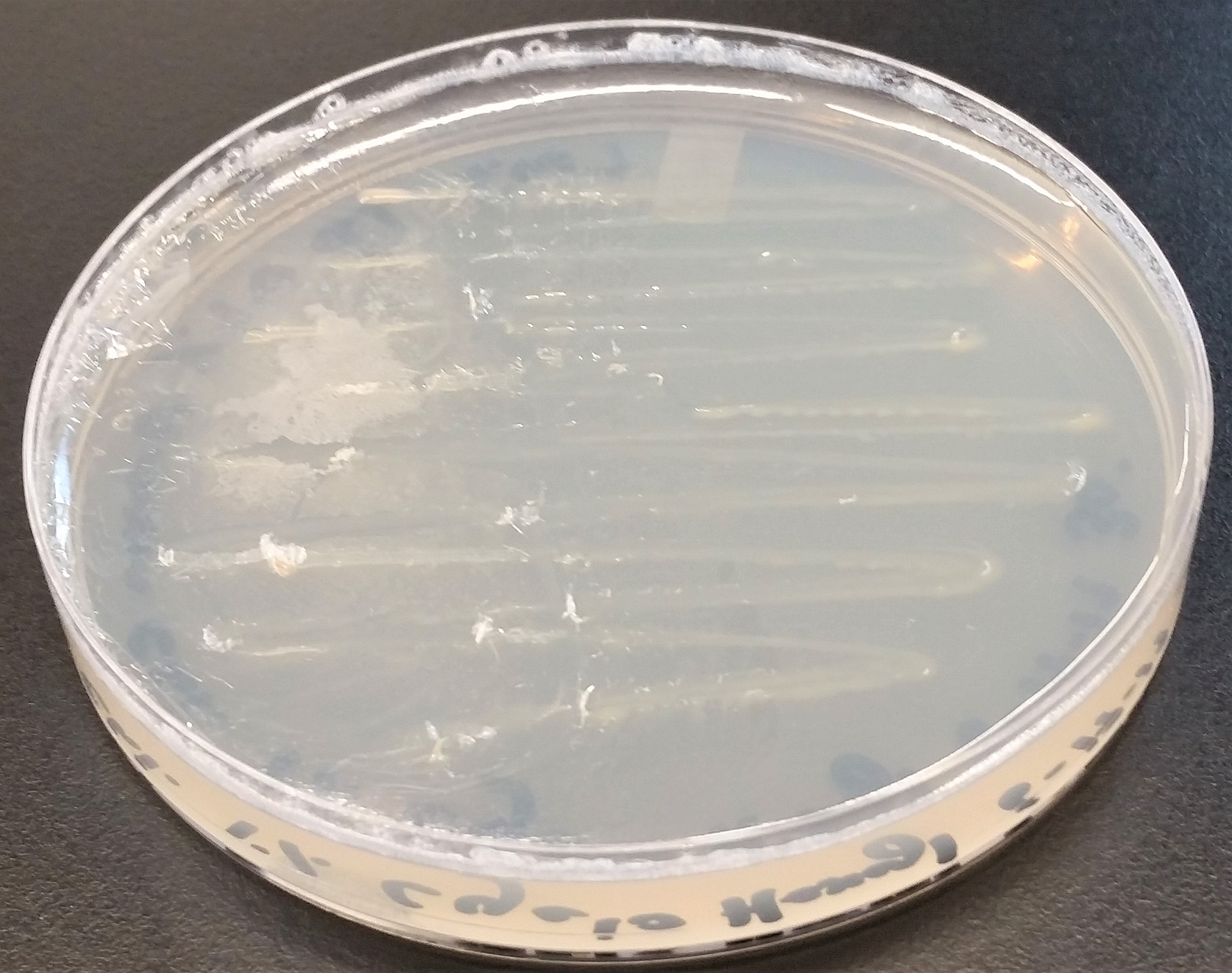

Supplement: Tests using Tris-Phosphate medium (TP) to see if hydrocarbons, aromatic compounds and polyhydroxyalkanoates can be used by the bacterium LMJ (Bacterium strain clone LIB091_C05_1243 variant 16S ribosomal RNA; GenBank Accession # MN633292.1) as the sole carbon source. — This file contains 23 images of TP (Tris-Phosphate) medium plates containing different alternative carbon sources. Bacterium LMJ was streaked on these chemical plates to test if LMJ can utilize these chemicals as the sole carbon source for energy and growth. 1% stocks of the following chemicals were tested: cyclohexyl chloride, phenanthrene, napthalene, benzoic acid, phenyl acetate. 2% (v/v) stocks of fresh and used car motor oil 10W30 were also tested. The doses used are given in mL in the file name. [file f1000research-9-27224-s0002.tgz › 4mLLMJcyclohexylchloride.jpg]

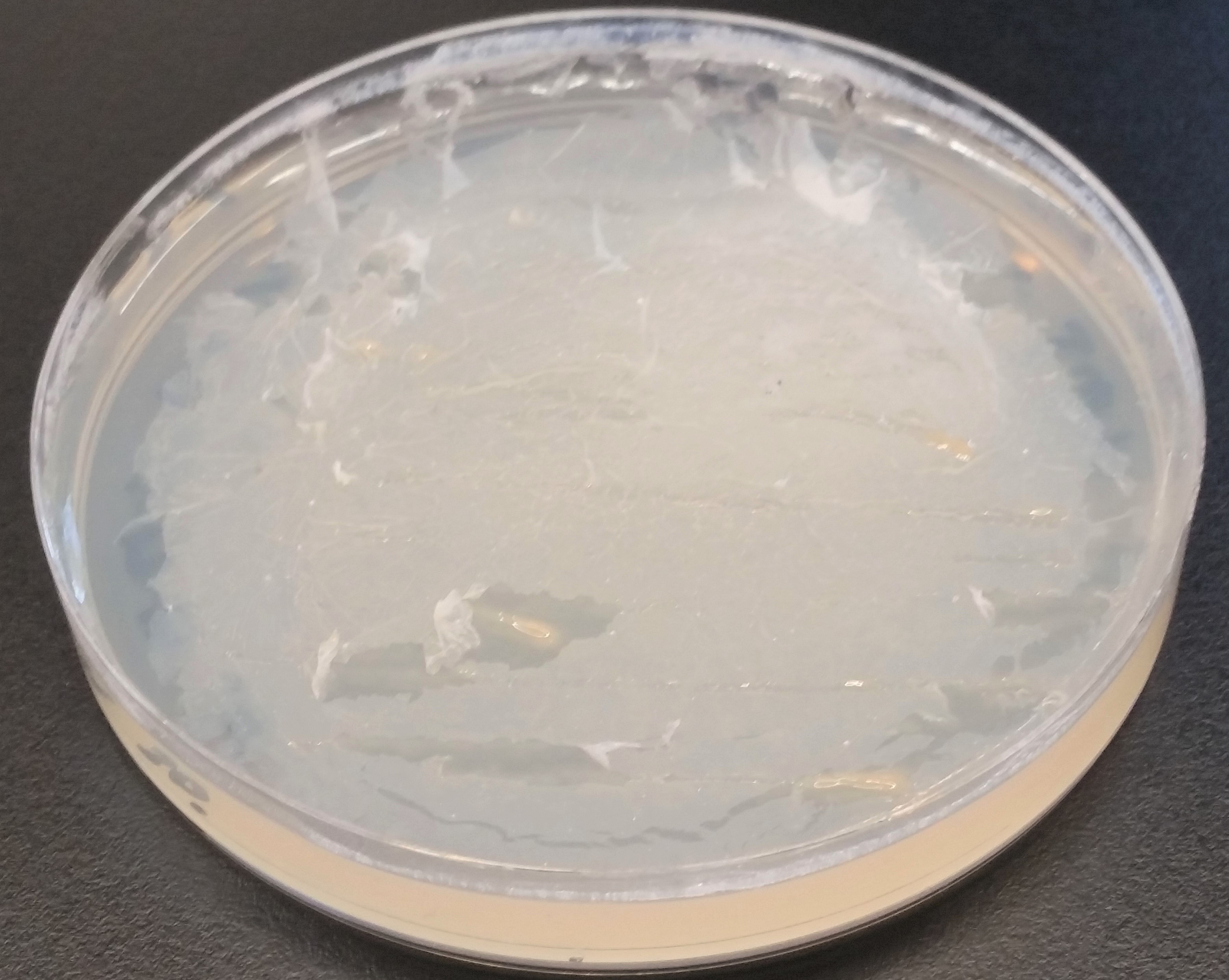

Supplement: Tests using Tris-Phosphate medium (TP) to see if hydrocarbons, aromatic compounds and polyhydroxyalkanoates can be used by the bacterium LMJ (Bacterium strain clone LIB091_C05_1243 variant 16S ribosomal RNA; GenBank Accession # MN633292.1) as the sole carbon source. — This file contains 23 images of TP (Tris-Phosphate) medium plates containing different alternative carbon sources. Bacterium LMJ was streaked on these chemical plates to test if LMJ can utilize these chemicals as the sole carbon source for energy and growth. 1% stocks of the following chemicals were tested: cyclohexyl chloride, phenanthrene, napthalene, benzoic acid, phenyl acetate. 2% (v/v) stocks of fresh and used car motor oil 10W30 were also tested. The doses used are given in mL in the file name. [file f1000research-9-27224-s0002.tgz › 4mLPHBLMJ.jpg]

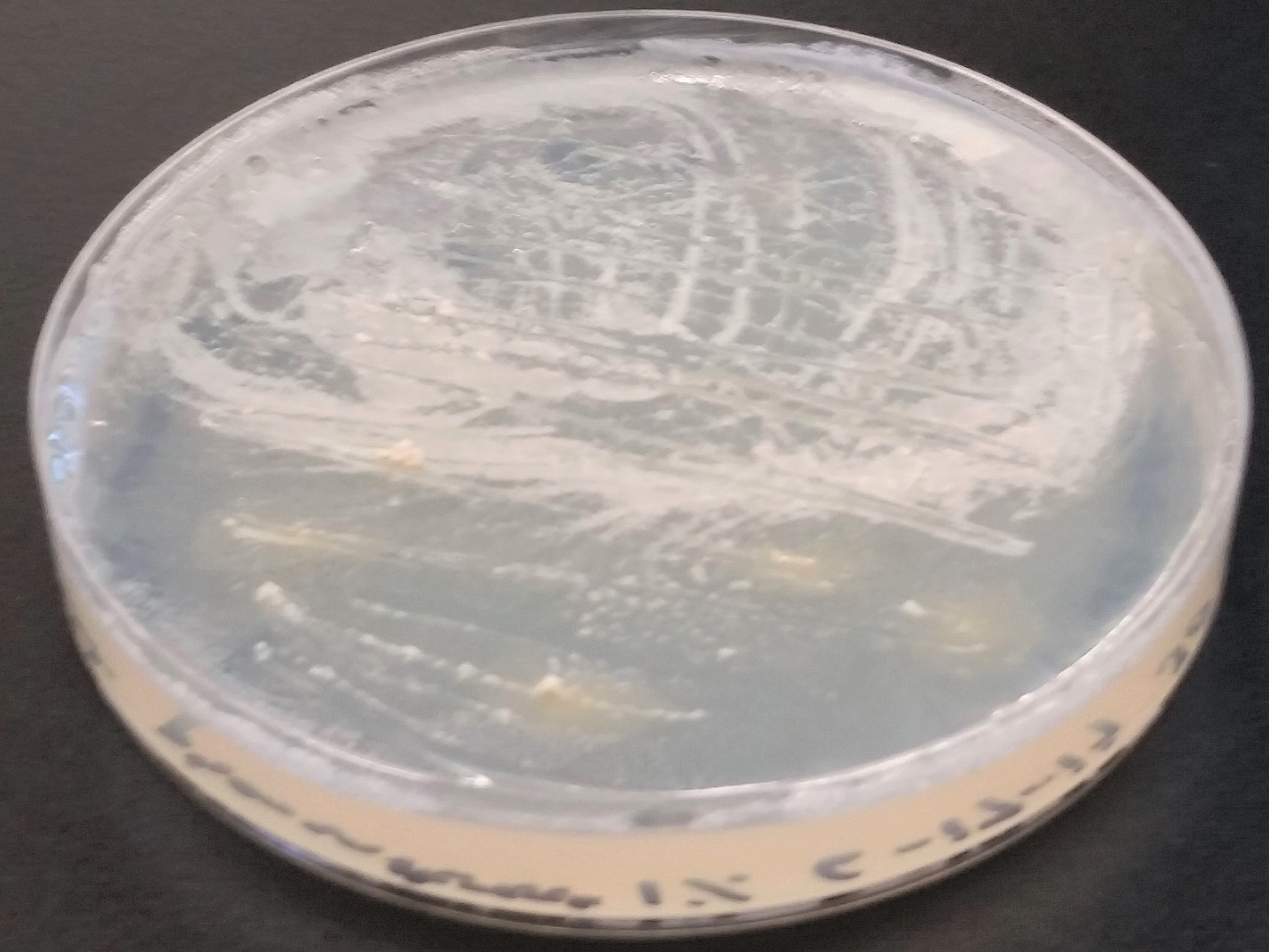

Supplement: Tests using Tris-Phosphate medium (TP) to see if hydrocarbons, aromatic compounds and polyhydroxyalkanoates can be used by the bacterium LMJ (Bacterium strain clone LIB091_C05_1243 variant 16S ribosomal RNA; GenBank Accession # MN633292.1) as the sole carbon source. — This file contains 23 images of TP (Tris-Phosphate) medium plates containing different alternative carbon sources. Bacterium LMJ was streaked on these chemical plates to test if LMJ can utilize these chemicals as the sole carbon source for energy and growth. 1% stocks of the following chemicals were tested: cyclohexyl chloride, phenanthrene, napthalene, benzoic acid, phenyl acetate. 2% (v/v) stocks of fresh and used car motor oil 10W30 were also tested. The doses used are given in mL in the file name. [file f1000research-9-27224-s0002.tgz › 4mLphenanthreneLMJ.jpg]

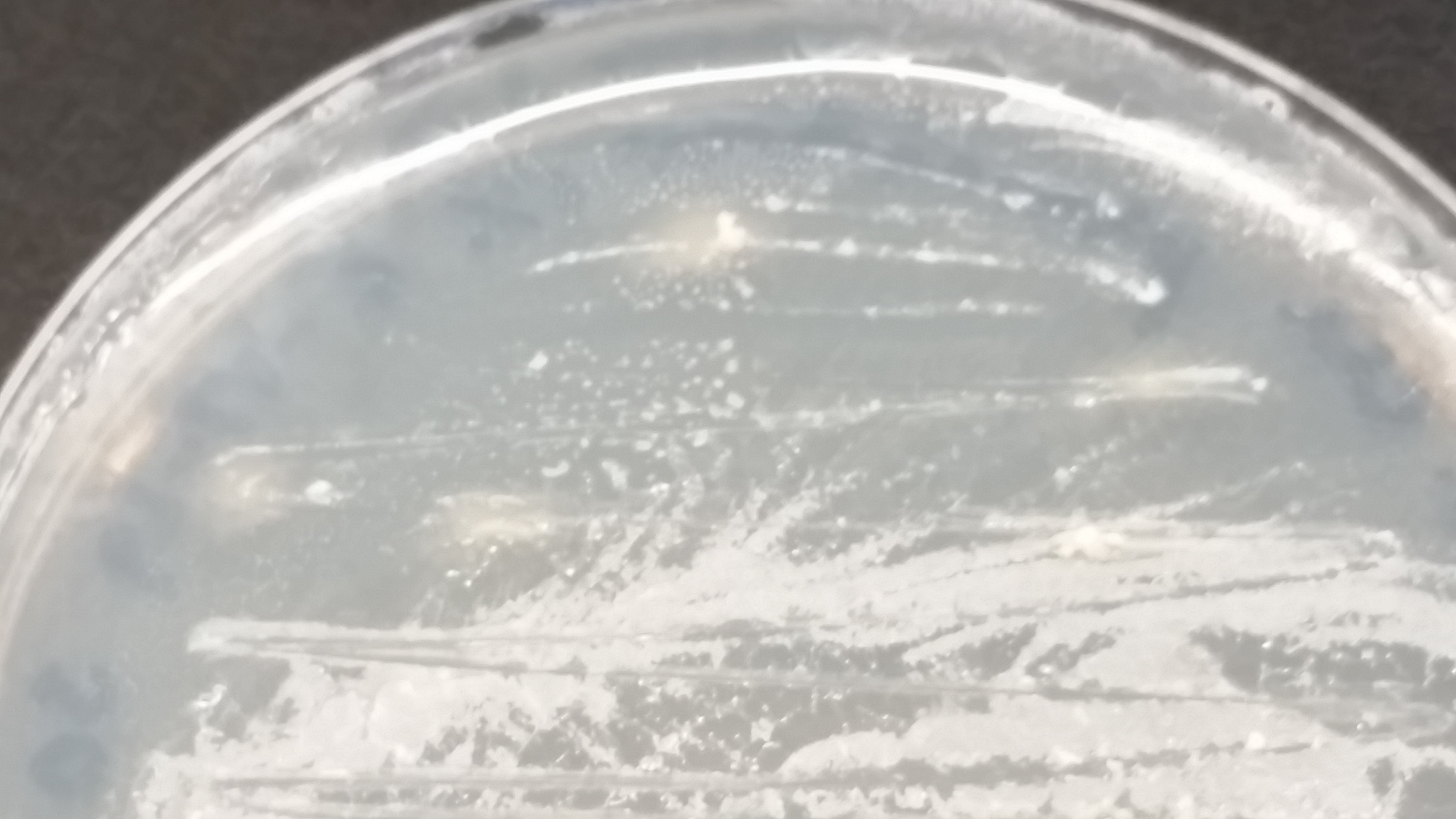

Supplement: Tests using Tris-Phosphate medium (TP) to see if hydrocarbons, aromatic compounds and polyhydroxyalkanoates can be used by the bacterium LMJ (Bacterium strain clone LIB091_C05_1243 variant 16S ribosomal RNA; GenBank Accession # MN633292.1) as the sole carbon source. — This file contains 23 images of TP (Tris-Phosphate) medium plates containing different alternative carbon sources. Bacterium LMJ was streaked on these chemical plates to test if LMJ can utilize these chemicals as the sole carbon source for energy and growth. 1% stocks of the following chemicals were tested: cyclohexyl chloride, phenanthrene, napthalene, benzoic acid, phenyl acetate. 2% (v/v) stocks of fresh and used car motor oil 10W30 were also tested. The doses used are given in mL in the file name. [file f1000research-9-27224-s0002.tgz › zoomupof4mLphenanthrene.jpg]

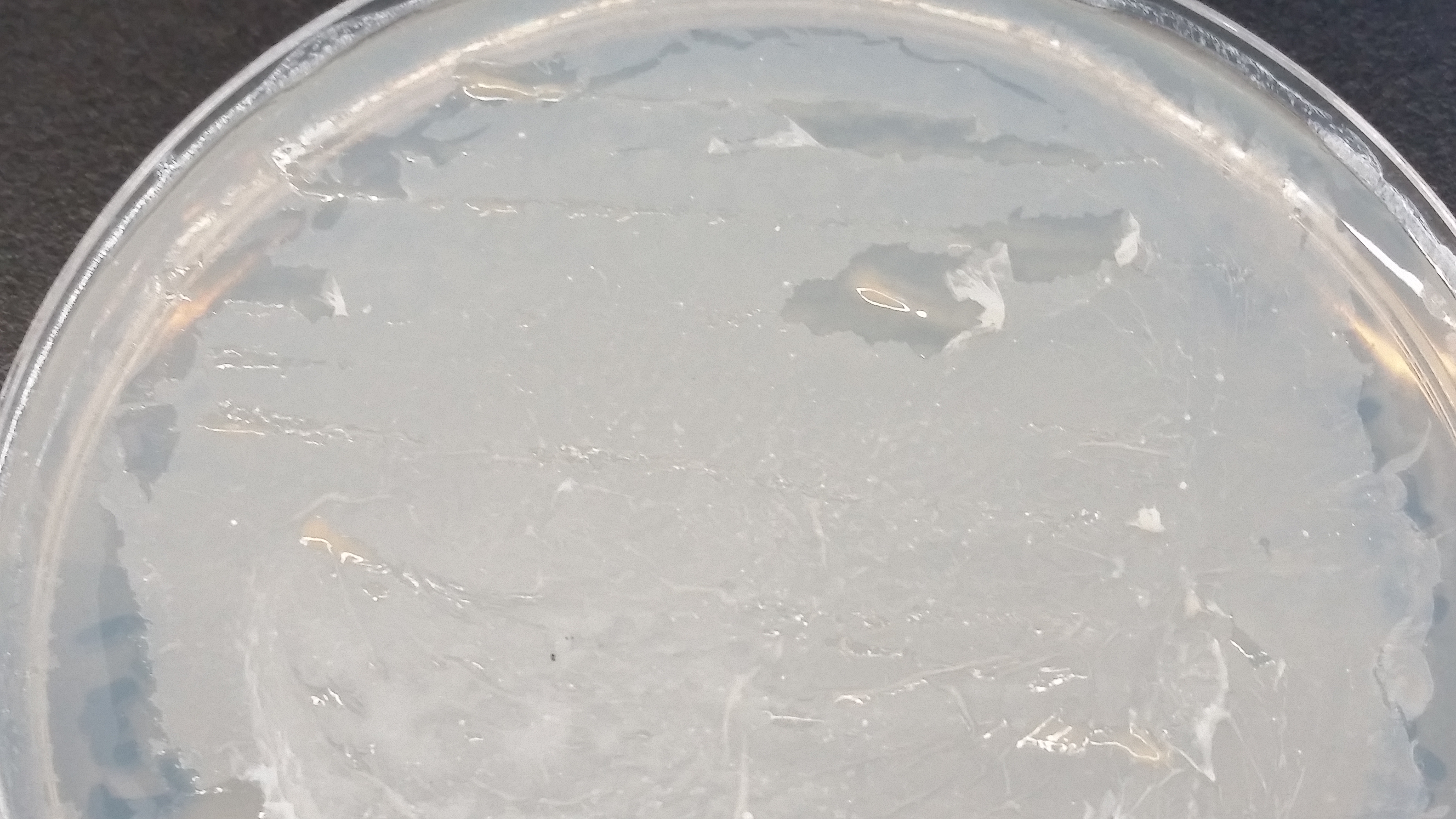

Supplement: Tests using Tris-Phosphate medium (TP) to see if hydrocarbons, aromatic compounds and polyhydroxyalkanoates can be used by the bacterium LMJ (Bacterium strain clone LIB091_C05_1243 variant 16S ribosomal RNA; GenBank Accession # MN633292.1) as the sole carbon source. — This file contains 23 images of TP (Tris-Phosphate) medium plates containing different alternative carbon sources. Bacterium LMJ was streaked on these chemical plates to test if LMJ can utilize these chemicals as the sole carbon source for energy and growth. 1% stocks of the following chemicals were tested: cyclohexyl chloride, phenanthrene, napthalene, benzoic acid, phenyl acetate. 2% (v/v) stocks of fresh and used car motor oil 10W30 were also tested. The doses used are given in mL in the file name. [file f1000research-9-27224-s0002.tgz › zoomupofLMJPHB4mL.jpg]

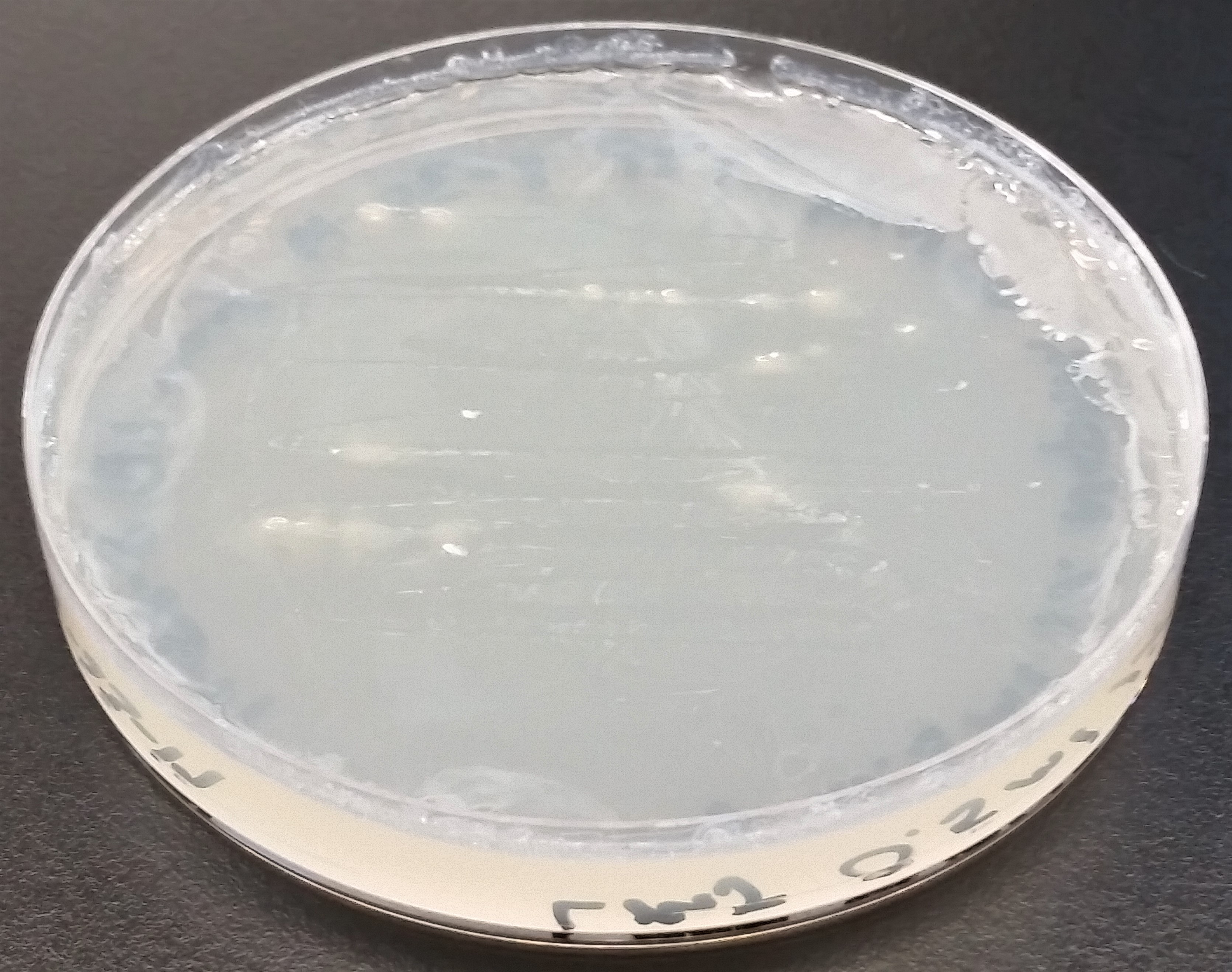

Supplement: Tests using Tris-Phosphate medium (TP) to see if hydrocarbons, aromatic compounds and polyhydroxyalkanoates can be used by the bacterium LMJ (Bacterium strain clone LIB091_C05_1243 variant 16S ribosomal RNA; GenBank Accession # MN633292.1) as the sole carbon source. — This file contains 23 images of TP (Tris-Phosphate) medium plates containing different alternative carbon sources. Bacterium LMJ was streaked on these chemical plates to test if LMJ can utilize these chemicals as the sole carbon source for energy and growth. 1% stocks of the following chemicals were tested: cyclohexyl chloride, phenanthrene, napthalene, benzoic acid, phenyl acetate. 2% (v/v) stocks of fresh and used car motor oil 10W30 were also tested. The doses used are given in mL in the file name. [file f1000research-9-27224-s0002.tgz › LMJ0.5mL1napthalene82319.jpg]

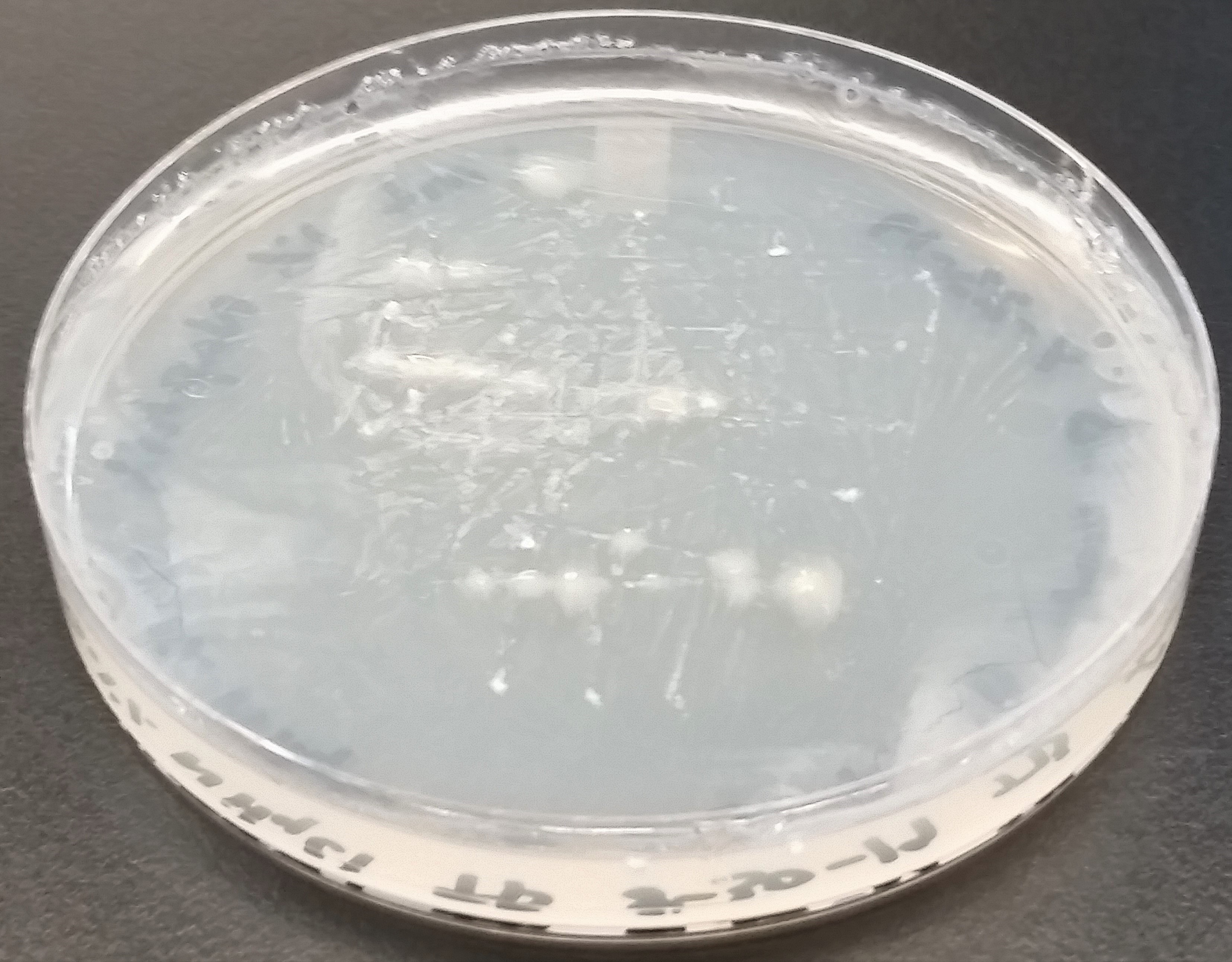

Supplement: Tests using Tris-Phosphate medium (TP) to see if hydrocarbons, aromatic compounds and polyhydroxyalkanoates can be used by the bacterium LMJ (Bacterium strain clone LIB091_C05_1243 variant 16S ribosomal RNA; GenBank Accession # MN633292.1) as the sole carbon source. — This file contains 23 images of TP (Tris-Phosphate) medium plates containing different alternative carbon sources. Bacterium LMJ was streaked on these chemical plates to test if LMJ can utilize these chemicals as the sole carbon source for energy and growth. 1% stocks of the following chemicals were tested: cyclohexyl chloride, phenanthrene, napthalene, benzoic acid, phenyl acetate. 2% (v/v) stocks of fresh and used car motor oil 10W30 were also tested. The doses used are given in mL in the file name. [file f1000research-9-27224-s0002.tgz › LMJ1ml1napthalene82319.jpg]

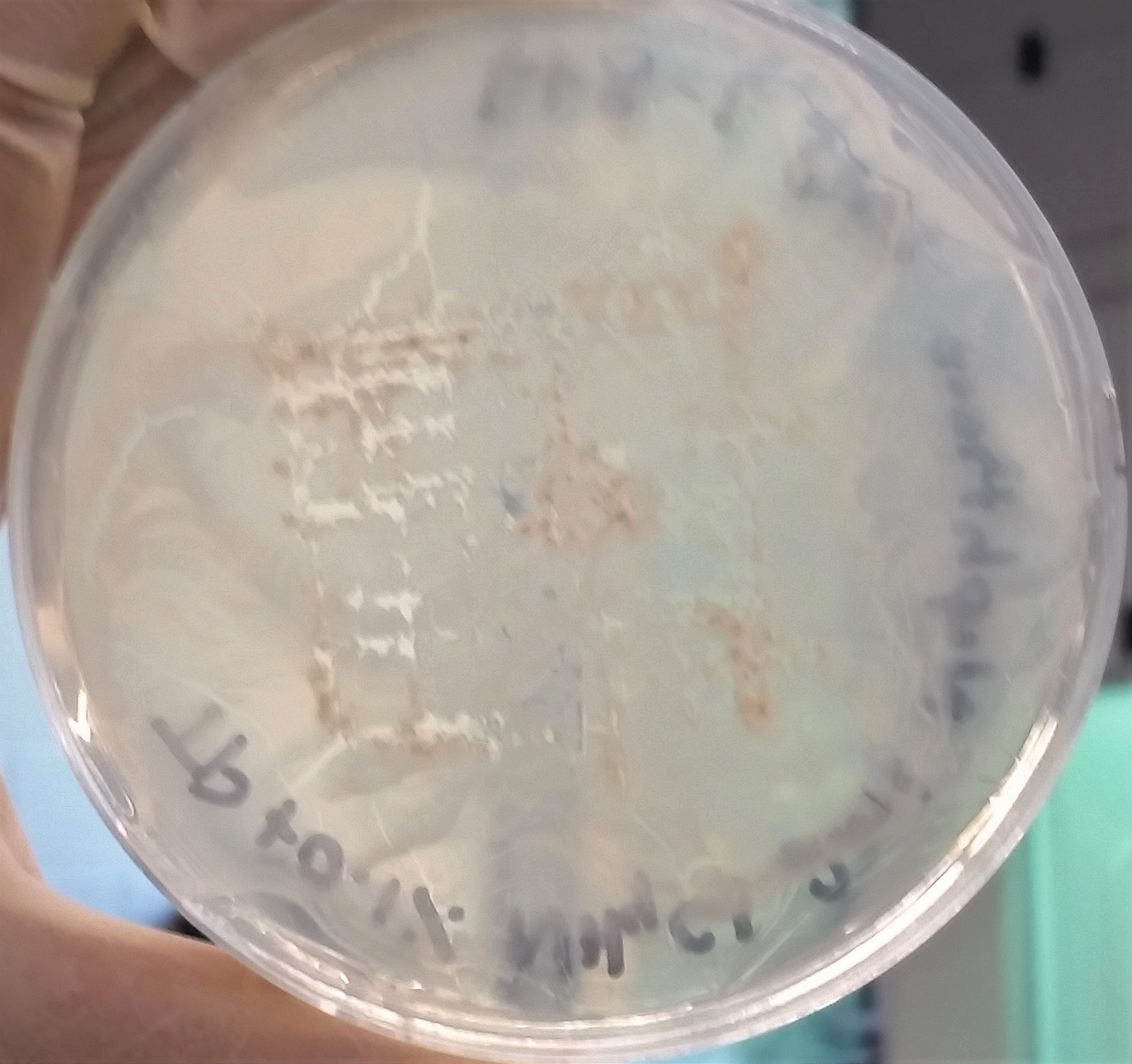

Supplement: Tests using Tris-Phosphate medium (TP) to see if hydrocarbons, aromatic compounds and polyhydroxyalkanoates can be used by the bacterium LMJ (Bacterium strain clone LIB091_C05_1243 variant 16S ribosomal RNA; GenBank Accession # MN633292.1) as the sole carbon source. — This file contains 23 images of TP (Tris-Phosphate) medium plates containing different alternative carbon sources. Bacterium LMJ was streaked on these chemical plates to test if LMJ can utilize these chemicals as the sole carbon source for energy and growth. 1% stocks of the following chemicals were tested: cyclohexyl chloride, phenanthrene, napthalene, benzoic acid, phenyl acetate. 2% (v/v) stocks of fresh and used car motor oil 10W30 were also tested. The doses used are given in mL in the file name. [file f1000research-9-27224-s0002.tgz › LMJnapthalene2mLzoomB.jpg]

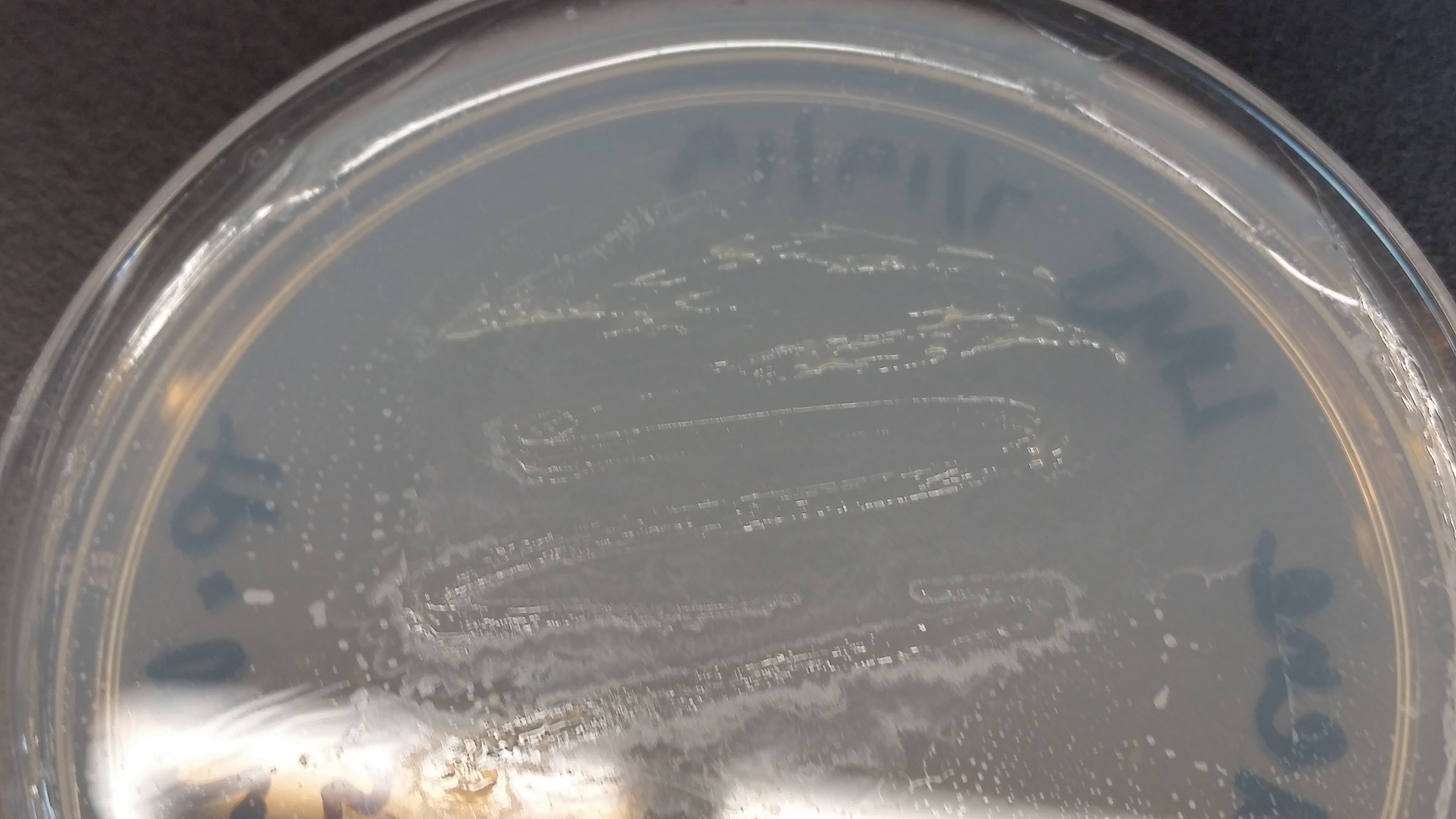

Supplement: Tests using Tris-Phosphate medium (TP) to see if hydrocarbons, aromatic compounds and polyhydroxyalkanoates can be used by the bacterium LMJ (Bacterium strain clone LIB091_C05_1243 variant 16S ribosomal RNA; GenBank Accession # MN633292.1) as the sole carbon source. — This file contains 23 images of TP (Tris-Phosphate) medium plates containing different alternative carbon sources. Bacterium LMJ was streaked on these chemical plates to test if LMJ can utilize these chemicals as the sole carbon source for energy and growth. 1% stocks of the following chemicals were tested: cyclohexyl chloride, phenanthrene, napthalene, benzoic acid, phenyl acetate. 2% (v/v) stocks of fresh and used car motor oil 10W30 were also tested. The doses used are given in mL in the file name. [file f1000research-9-27224-s0002.tgz › LMJzoomupof0.5mLnapthalene.jpg]

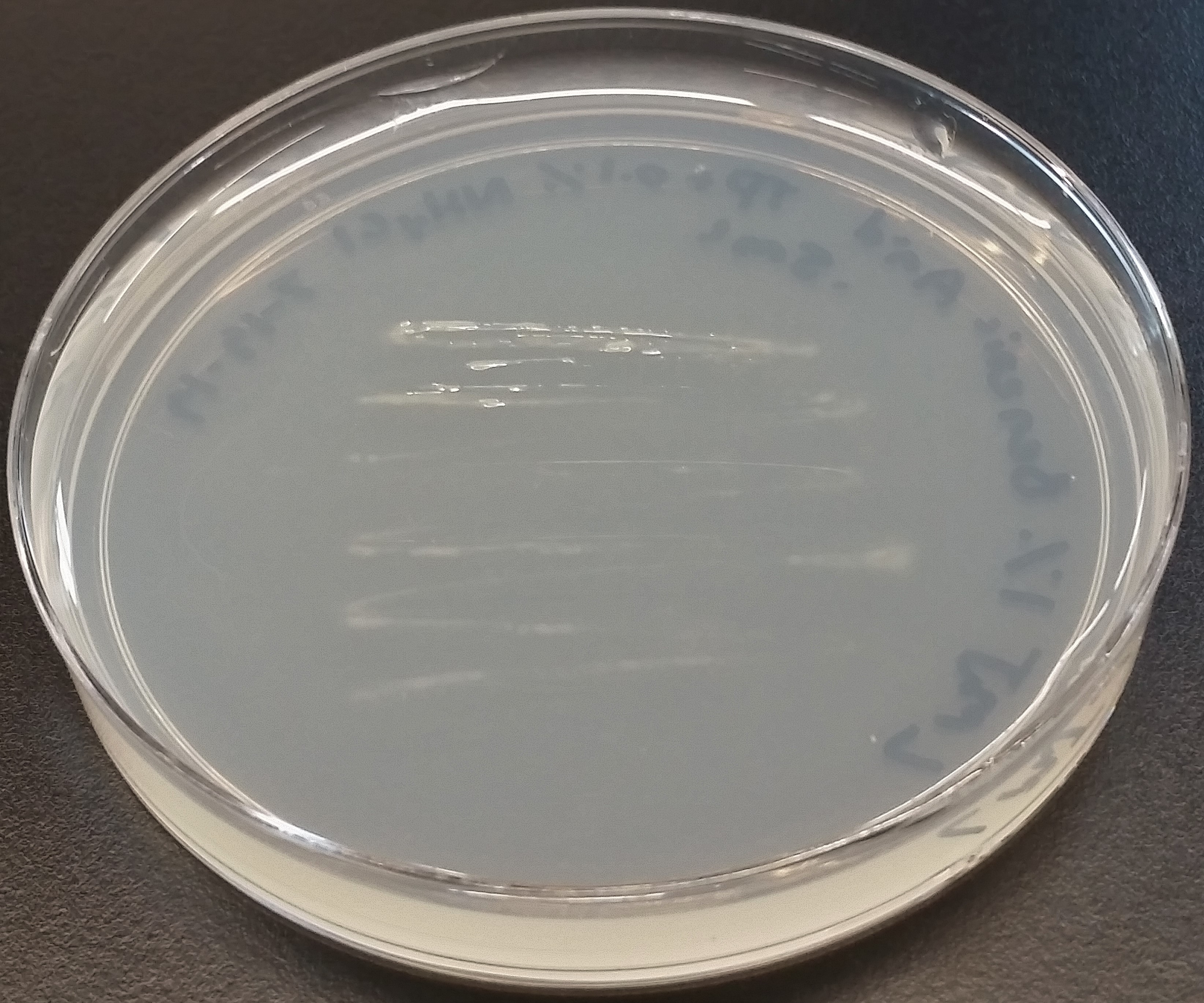

Supplement: Tests using Tris-Phosphate medium (TP) to see if hydrocarbons, aromatic compounds and polyhydroxyalkanoates can be used by the bacterium LMJ (Bacterium strain clone LIB091_C05_1243 variant 16S ribosomal RNA; GenBank Accession # MN633292.1) as the sole carbon source. — This file contains 23 images of TP (Tris-Phosphate) medium plates containing different alternative carbon sources. Bacterium LMJ was streaked on these chemical plates to test if LMJ can utilize these chemicals as the sole carbon source for energy and growth. 1% stocks of the following chemicals were tested: cyclohexyl chloride, phenanthrene, napthalene, benzoic acid, phenyl acetate. 2% (v/v) stocks of fresh and used car motor oil 10W30 were also tested. The doses used are given in mL in the file name. [file f1000research-9-27224-s0002.tgz › LMJ0.5mLbenzoicacid.jpg]

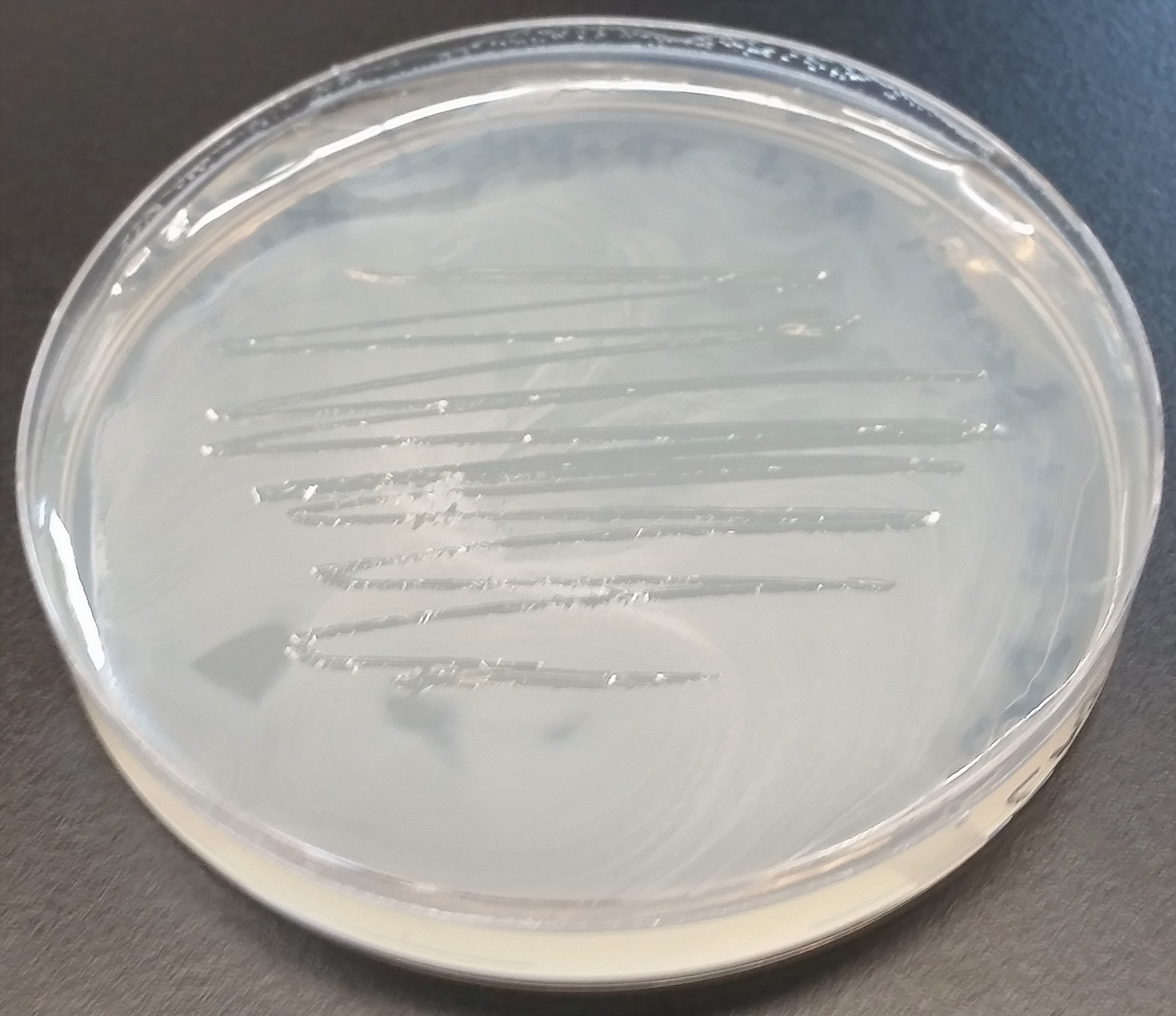

Supplement: Tests using Tris-Phosphate medium (TP) to see if hydrocarbons, aromatic compounds and polyhydroxyalkanoates can be used by the bacterium LMJ (Bacterium strain clone LIB091_C05_1243 variant 16S ribosomal RNA; GenBank Accession # MN633292.1) as the sole carbon source. — This file contains 23 images of TP (Tris-Phosphate) medium plates containing different alternative carbon sources. Bacterium LMJ was streaked on these chemical plates to test if LMJ can utilize these chemicals as the sole carbon source for energy and growth. 1% stocks of the following chemicals were tested: cyclohexyl chloride, phenanthrene, napthalene, benzoic acid, phenyl acetate. 2% (v/v) stocks of fresh and used car motor oil 10W30 were also tested. The doses used are given in mL in the file name. [file f1000research-9-27224-s0002.tgz › LMJ1mLbenzoicacid.jpg]

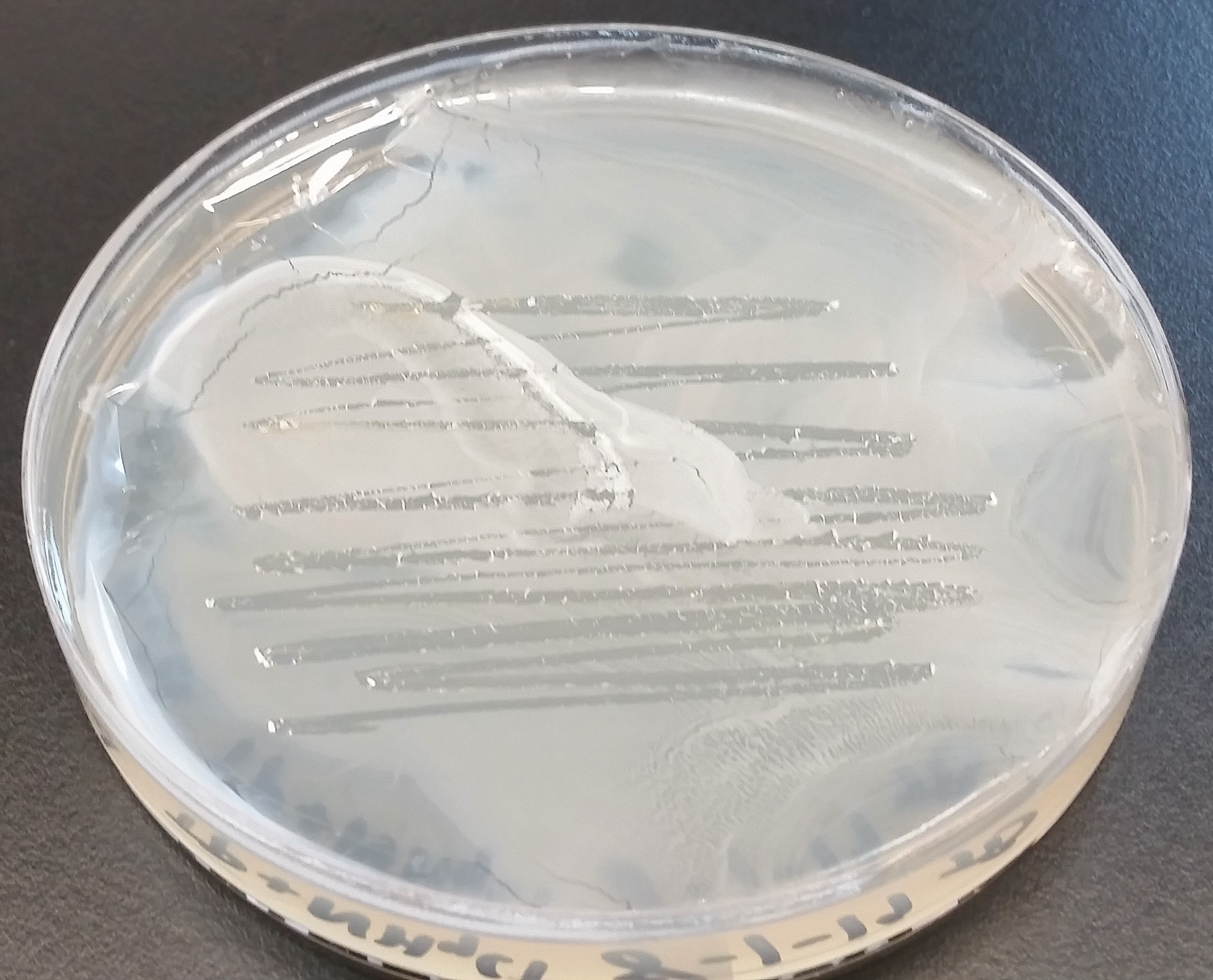

Supplement: Tests using Tris-Phosphate medium (TP) to see if hydrocarbons, aromatic compounds and polyhydroxyalkanoates can be used by the bacterium LMJ (Bacterium strain clone LIB091_C05_1243 variant 16S ribosomal RNA; GenBank Accession # MN633292.1) as the sole carbon source. — This file contains 23 images of TP (Tris-Phosphate) medium plates containing different alternative carbon sources. Bacterium LMJ was streaked on these chemical plates to test if LMJ can utilize these chemicals as the sole carbon source for energy and growth. 1% stocks of the following chemicals were tested: cyclohexyl chloride, phenanthrene, napthalene, benzoic acid, phenyl acetate. 2% (v/v) stocks of fresh and used car motor oil 10W30 were also tested. The doses used are given in mL in the file name. [file f1000research-9-27224-s0002.tgz › LMJ2mLbenzoicacid.jpg]

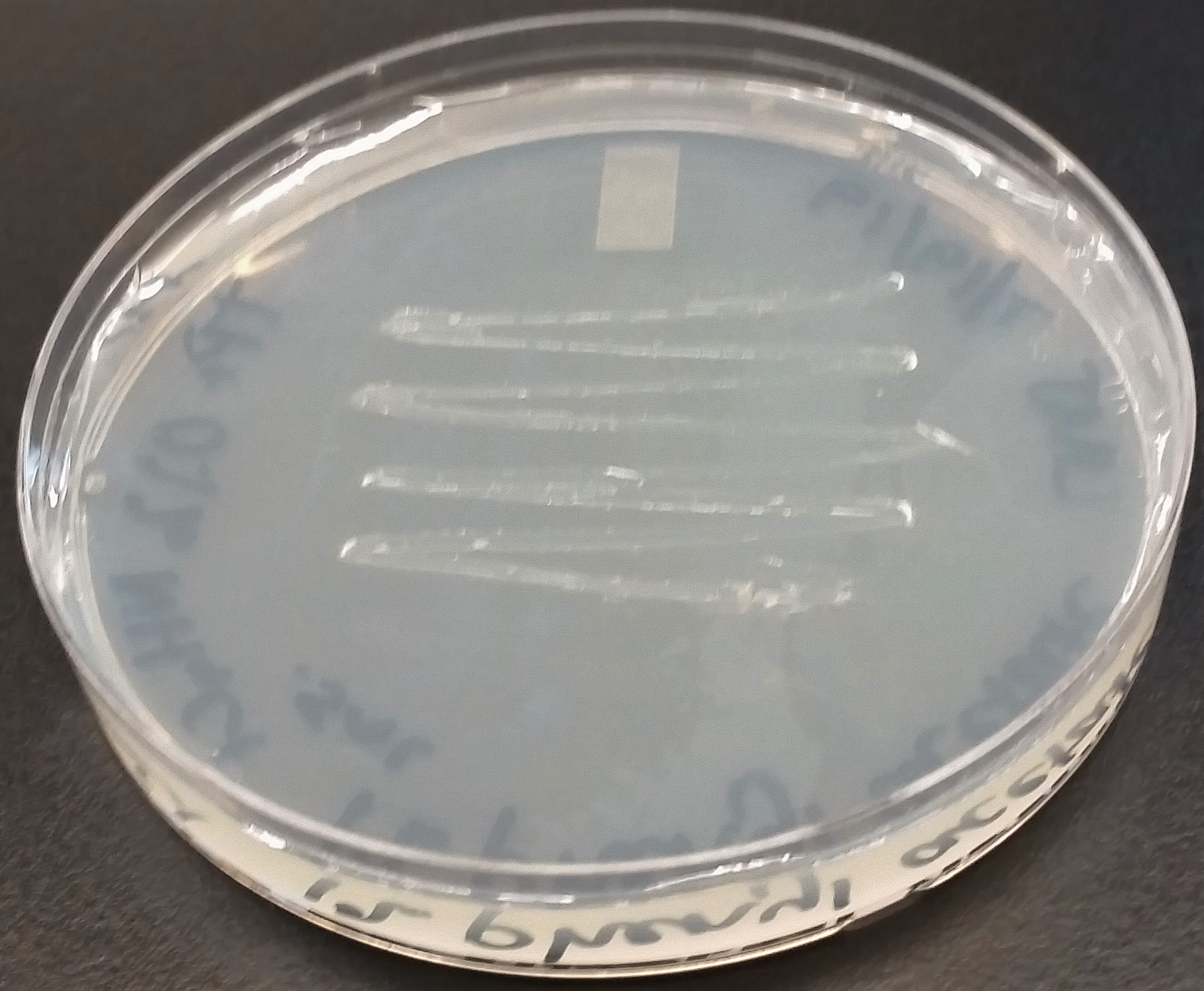

Supplement: Tests using Tris-Phosphate medium (TP) to see if hydrocarbons, aromatic compounds and polyhydroxyalkanoates can be used by the bacterium LMJ (Bacterium strain clone LIB091_C05_1243 variant 16S ribosomal RNA; GenBank Accession # MN633292.1) as the sole carbon source. — This file contains 23 images of TP (Tris-Phosphate) medium plates containing different alternative carbon sources. Bacterium LMJ was streaked on these chemical plates to test if LMJ can utilize these chemicals as the sole carbon source for energy and growth. 1% stocks of the following chemicals were tested: cyclohexyl chloride, phenanthrene, napthalene, benzoic acid, phenyl acetate. 2% (v/v) stocks of fresh and used car motor oil 10W30 were also tested. The doses used are given in mL in the file name. [file f1000research-9-27224-s0002.tgz › LMJ0.5mLphenylacetate.jpg]

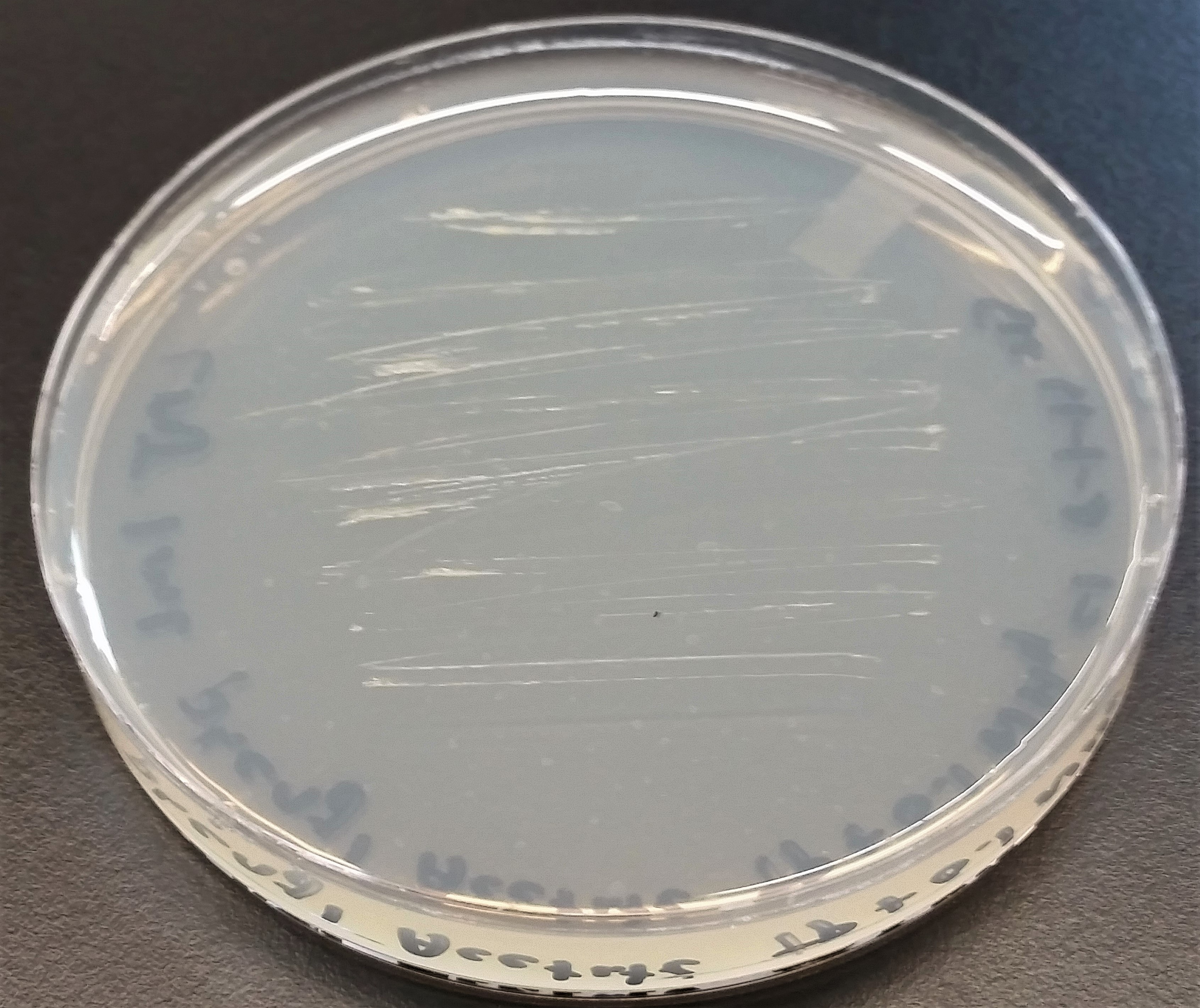

Supplement: Tests using Tris-Phosphate medium (TP) to see if hydrocarbons, aromatic compounds and polyhydroxyalkanoates can be used by the bacterium LMJ (Bacterium strain clone LIB091_C05_1243 variant 16S ribosomal RNA; GenBank Accession # MN633292.1) as the sole carbon source. — This file contains 23 images of TP (Tris-Phosphate) medium plates containing different alternative carbon sources. Bacterium LMJ was streaked on these chemical plates to test if LMJ can utilize these chemicals as the sole carbon source for energy and growth. 1% stocks of the following chemicals were tested: cyclohexyl chloride, phenanthrene, napthalene, benzoic acid, phenyl acetate. 2% (v/v) stocks of fresh and used car motor oil 10W30 were also tested. The doses used are given in mL in the file name. [file f1000research-9-27224-s0002.tgz › LMJ1mLphenylacetate.jpg]

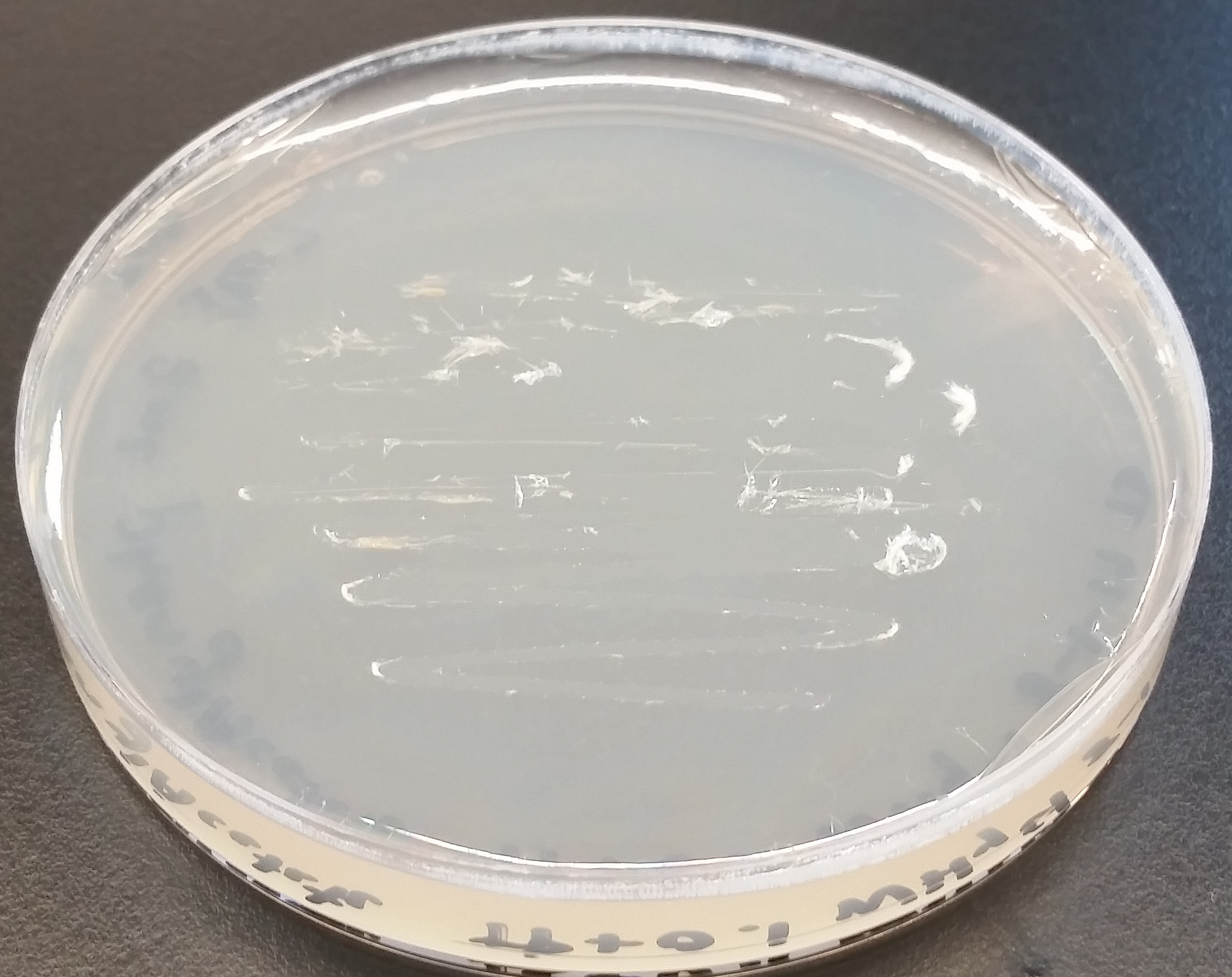

Supplement: Tests using Tris-Phosphate medium (TP) to see if hydrocarbons, aromatic compounds and polyhydroxyalkanoates can be used by the bacterium LMJ (Bacterium strain clone LIB091_C05_1243 variant 16S ribosomal RNA; GenBank Accession # MN633292.1) as the sole carbon source. — This file contains 23 images of TP (Tris-Phosphate) medium plates containing different alternative carbon sources. Bacterium LMJ was streaked on these chemical plates to test if LMJ can utilize these chemicals as the sole carbon source for energy and growth. 1% stocks of the following chemicals were tested: cyclohexyl chloride, phenanthrene, napthalene, benzoic acid, phenyl acetate. 2% (v/v) stocks of fresh and used car motor oil 10W30 were also tested. The doses used are given in mL in the file name. [file f1000research-9-27224-s0002.tgz › LMJ2mLphenylacetaterep.jpg]

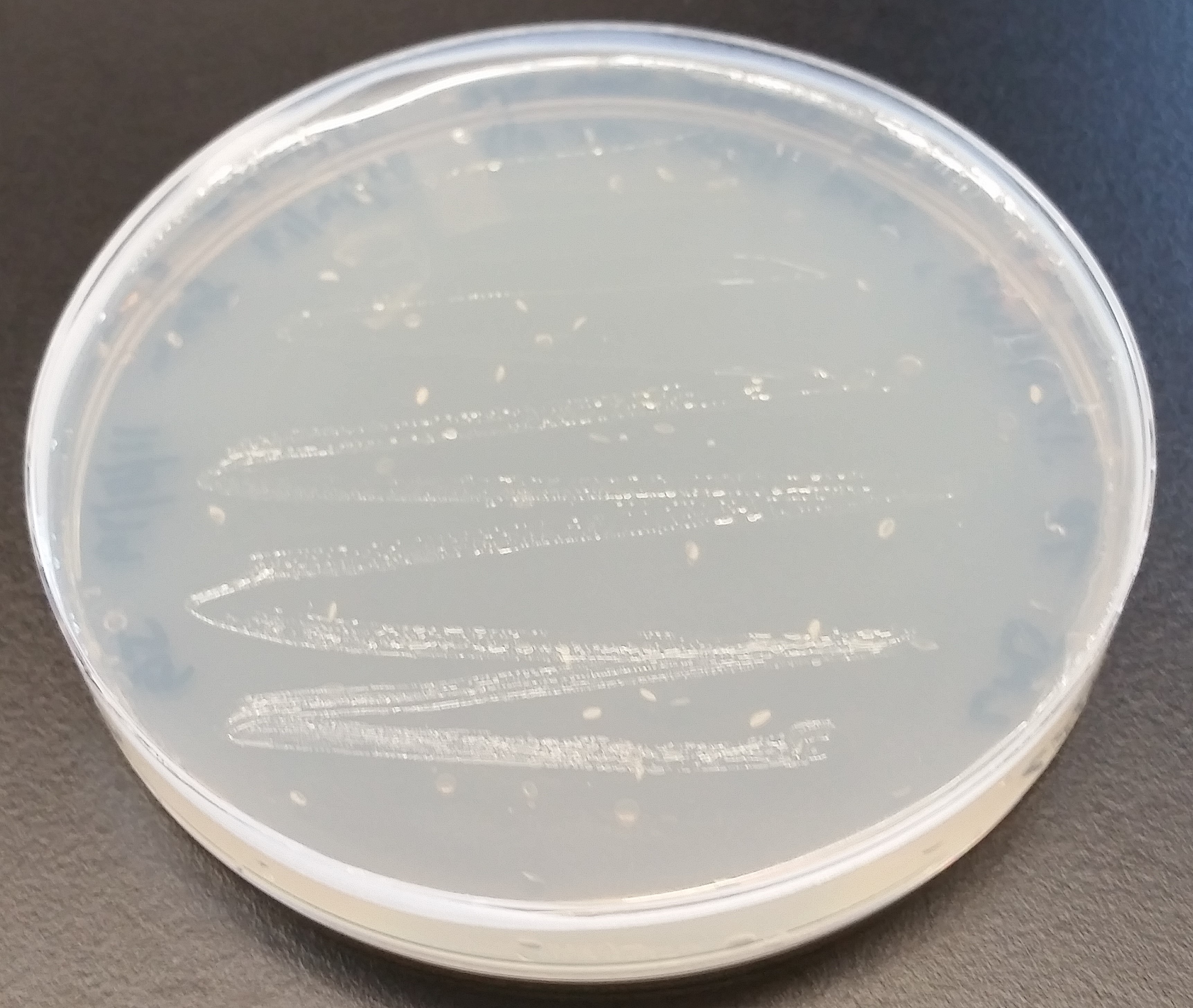

Supplement: Tests using Tris-Phosphate medium (TP) to see if hydrocarbons, aromatic compounds and polyhydroxyalkanoates can be used by the bacterium LMJ (Bacterium strain clone LIB091_C05_1243 variant 16S ribosomal RNA; GenBank Accession # MN633292.1) as the sole carbon source. — This file contains 23 images of TP (Tris-Phosphate) medium plates containing different alternative carbon sources. Bacterium LMJ was streaked on these chemical plates to test if LMJ can utilize these chemicals as the sole carbon source for energy and growth. 1% stocks of the following chemicals were tested: cyclohexyl chloride, phenanthrene, napthalene, benzoic acid, phenyl acetate. 2% (v/v) stocks of fresh and used car motor oil 10W30 were also tested. The doses used are given in mL in the file name. [file f1000research-9-27224-s0002.tgz › 10W30LMJ0.5mLfresh.jpg]

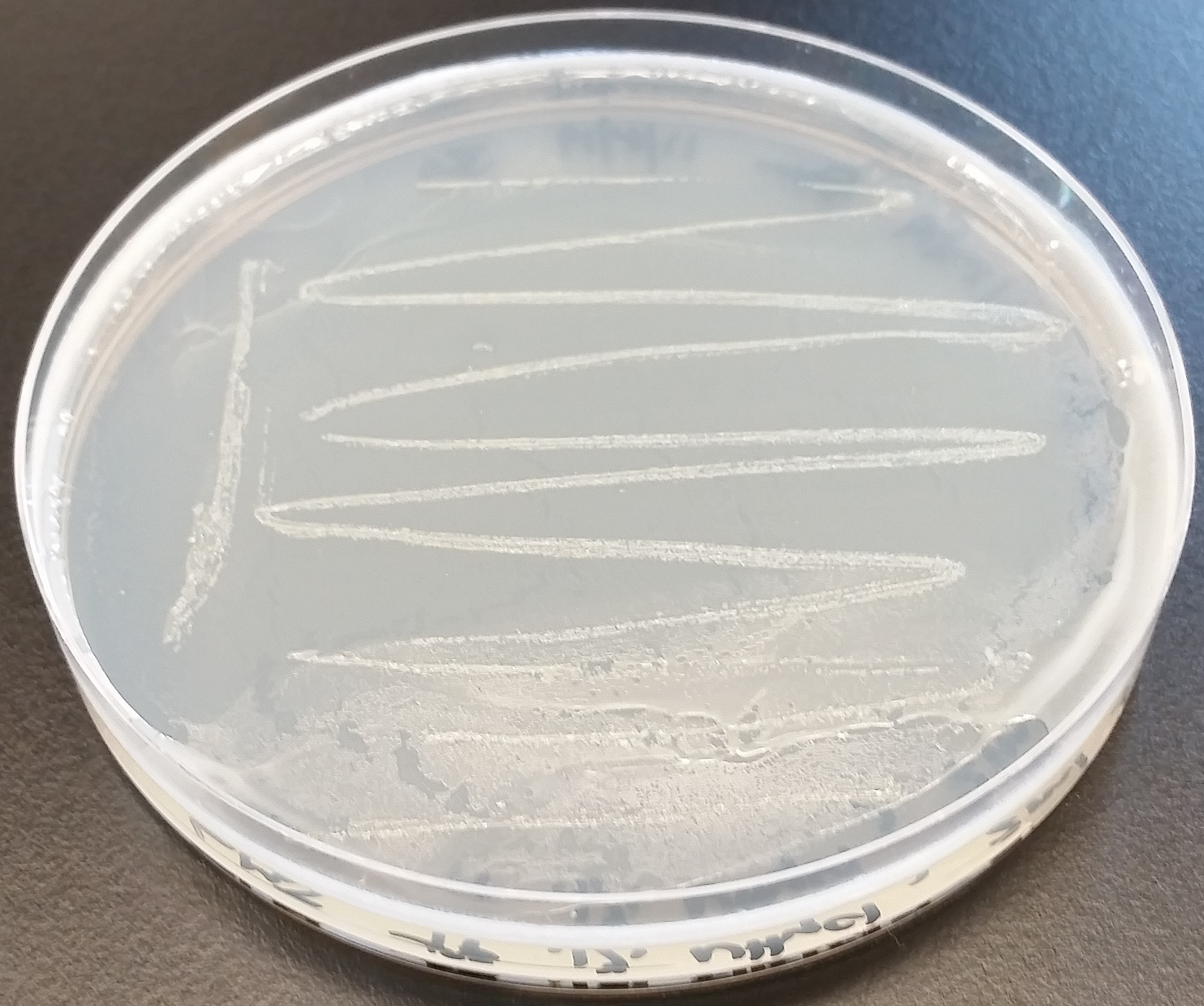

Supplement: Tests using Tris-Phosphate medium (TP) to see if hydrocarbons, aromatic compounds and polyhydroxyalkanoates can be used by the bacterium LMJ (Bacterium strain clone LIB091_C05_1243 variant 16S ribosomal RNA; GenBank Accession # MN633292.1) as the sole carbon source. — This file contains 23 images of TP (Tris-Phosphate) medium plates containing different alternative carbon sources. Bacterium LMJ was streaked on these chemical plates to test if LMJ can utilize these chemicals as the sole carbon source for energy and growth. 1% stocks of the following chemicals were tested: cyclohexyl chloride, phenanthrene, napthalene, benzoic acid, phenyl acetate. 2% (v/v) stocks of fresh and used car motor oil 10W30 were also tested. The doses used are given in mL in the file name. [file f1000research-9-27224-s0002.tgz › 10W30LMJ2mLfresh.jpg]

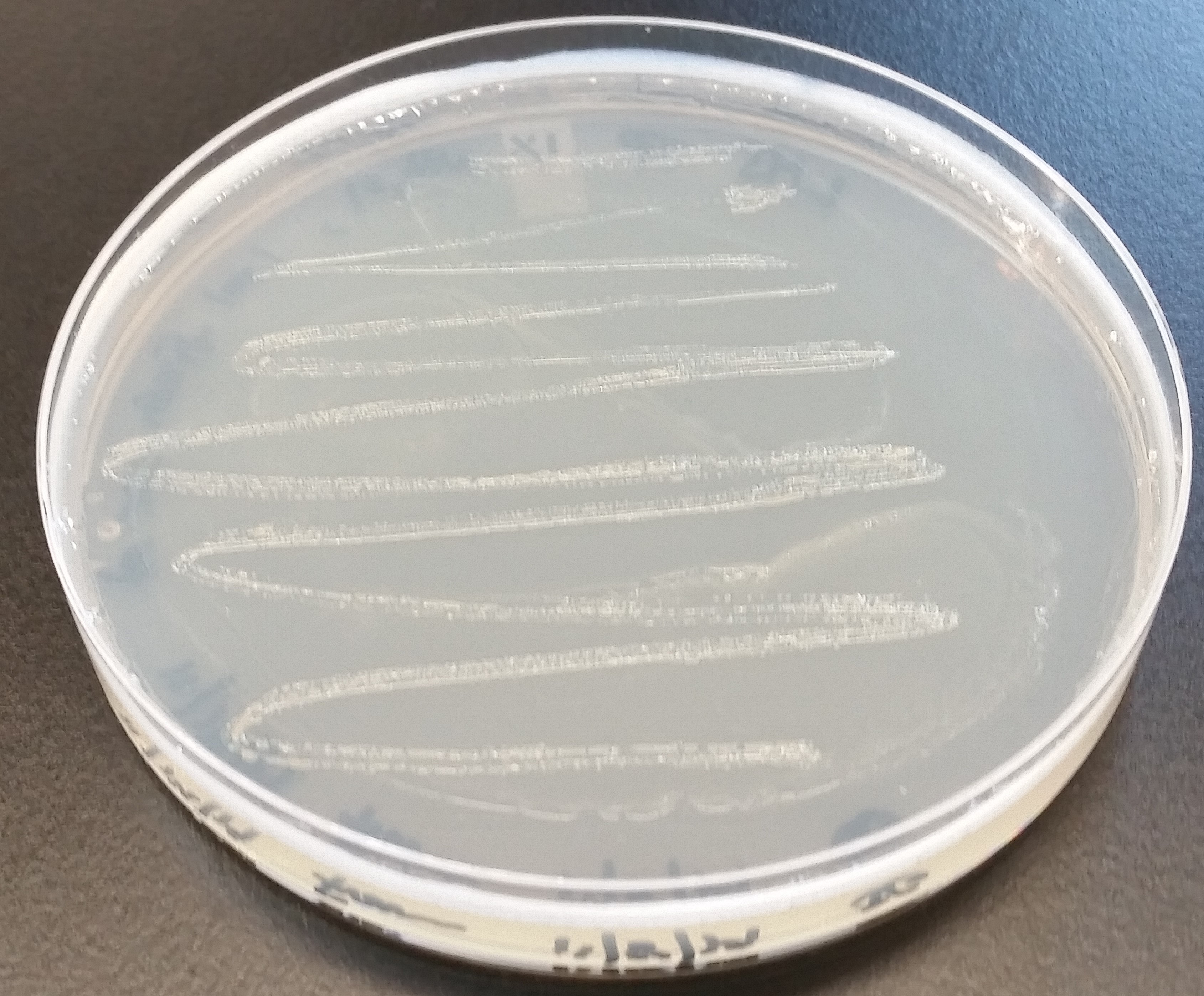

Supplement: Tests using Tris-Phosphate medium (TP) to see if hydrocarbons, aromatic compounds and polyhydroxyalkanoates can be used by the bacterium LMJ (Bacterium strain clone LIB091_C05_1243 variant 16S ribosomal RNA; GenBank Accession # MN633292.1) as the sole carbon source. — This file contains 23 images of TP (Tris-Phosphate) medium plates containing different alternative carbon sources. Bacterium LMJ was streaked on these chemical plates to test if LMJ can utilize these chemicals as the sole carbon source for energy and growth. 1% stocks of the following chemicals were tested: cyclohexyl chloride, phenanthrene, napthalene, benzoic acid, phenyl acetate. 2% (v/v) stocks of fresh and used car motor oil 10W30 were also tested. The doses used are given in mL in the file name. [file f1000research-9-27224-s0002.tgz › 10W30LMJ1mLfresh.jpg]

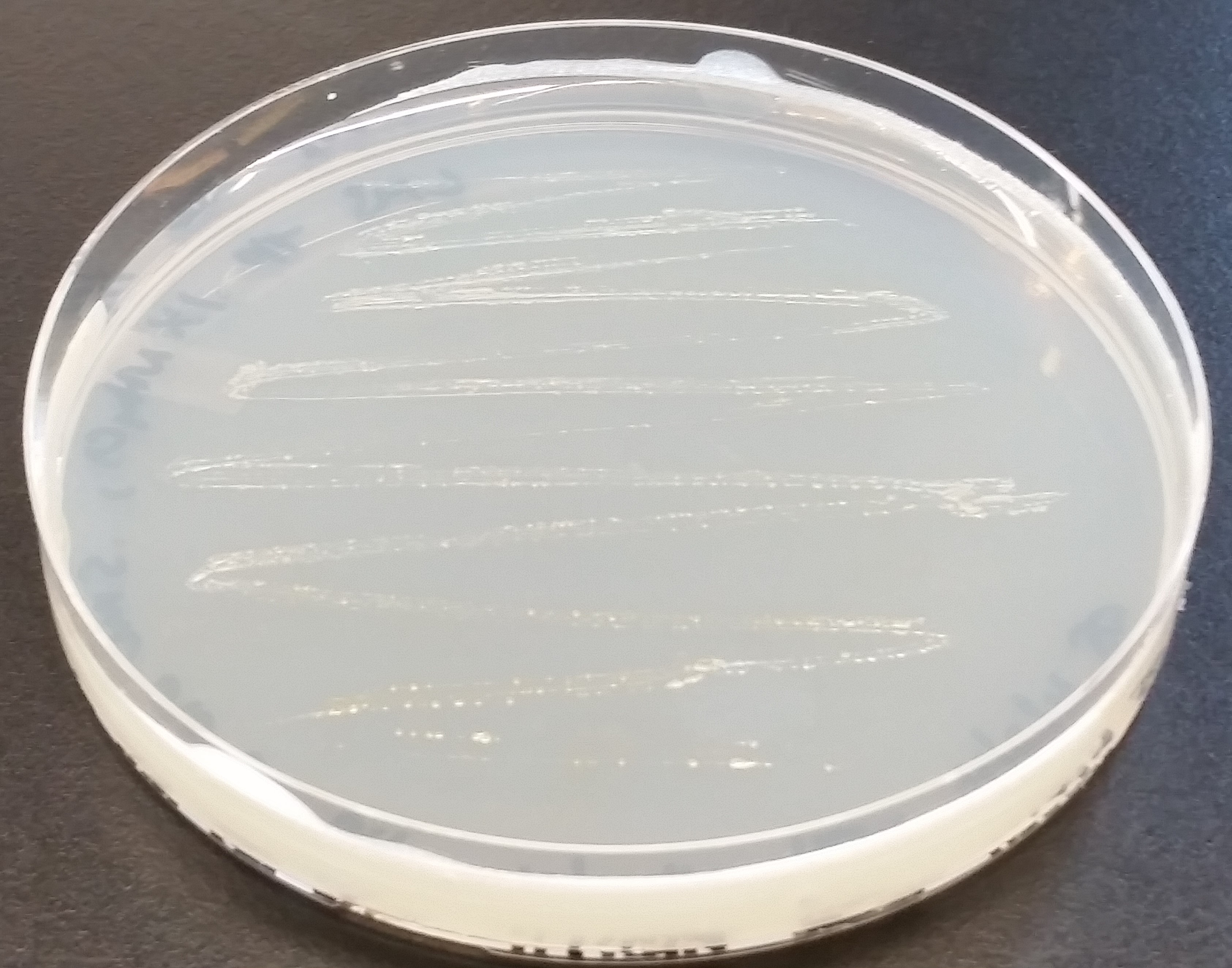

Supplement: Tests using Tris-Phosphate medium (TP) to see if hydrocarbons, aromatic compounds and polyhydroxyalkanoates can be used by the bacterium LMJ (Bacterium strain clone LIB091_C05_1243 variant 16S ribosomal RNA; GenBank Accession # MN633292.1) as the sole carbon source. — This file contains 23 images of TP (Tris-Phosphate) medium plates containing different alternative carbon sources. Bacterium LMJ was streaked on these chemical plates to test if LMJ can utilize these chemicals as the sole carbon source for energy and growth. 1% stocks of the following chemicals were tested: cyclohexyl chloride, phenanthrene, napthalene, benzoic acid, phenyl acetate. 2% (v/v) stocks of fresh and used car motor oil 10W30 were also tested. The doses used are given in mL in the file name. [file f1000research-9-27224-s0002.tgz › 10W30usedLMJ0.5mL.jpg]

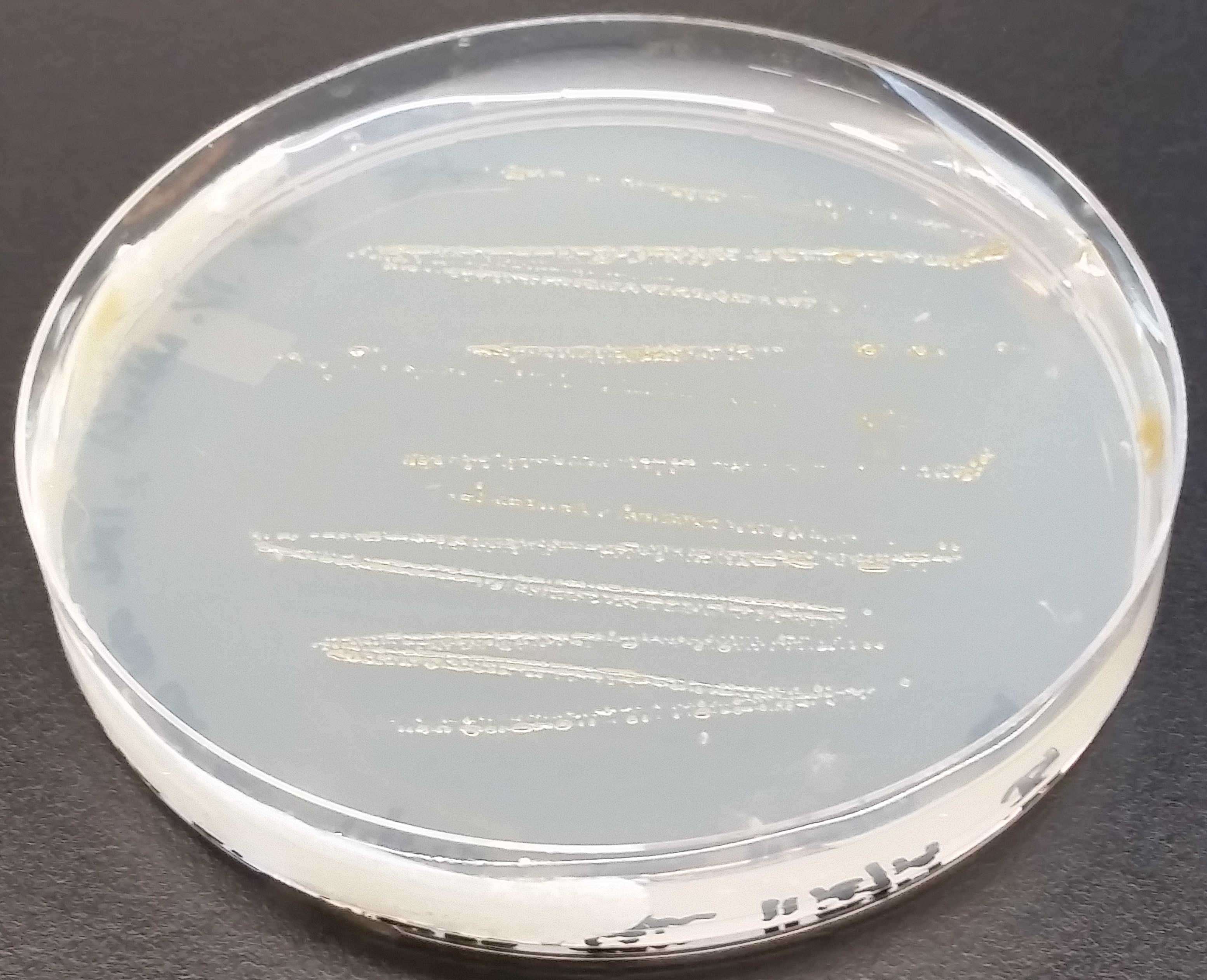

Supplement: Tests using Tris-Phosphate medium (TP) to see if hydrocarbons, aromatic compounds and polyhydroxyalkanoates can be used by the bacterium LMJ (Bacterium strain clone LIB091_C05_1243 variant 16S ribosomal RNA; GenBank Accession # MN633292.1) as the sole carbon source. — This file contains 23 images of TP (Tris-Phosphate) medium plates containing different alternative carbon sources. Bacterium LMJ was streaked on these chemical plates to test if LMJ can utilize these chemicals as the sole carbon source for energy and growth. 1% stocks of the following chemicals were tested: cyclohexyl chloride, phenanthrene, napthalene, benzoic acid, phenyl acetate. 2% (v/v) stocks of fresh and used car motor oil 10W30 were also tested. The doses used are given in mL in the file name. [file f1000research-9-27224-s0002.tgz › 10W30usedLMJ1mL.jpg]

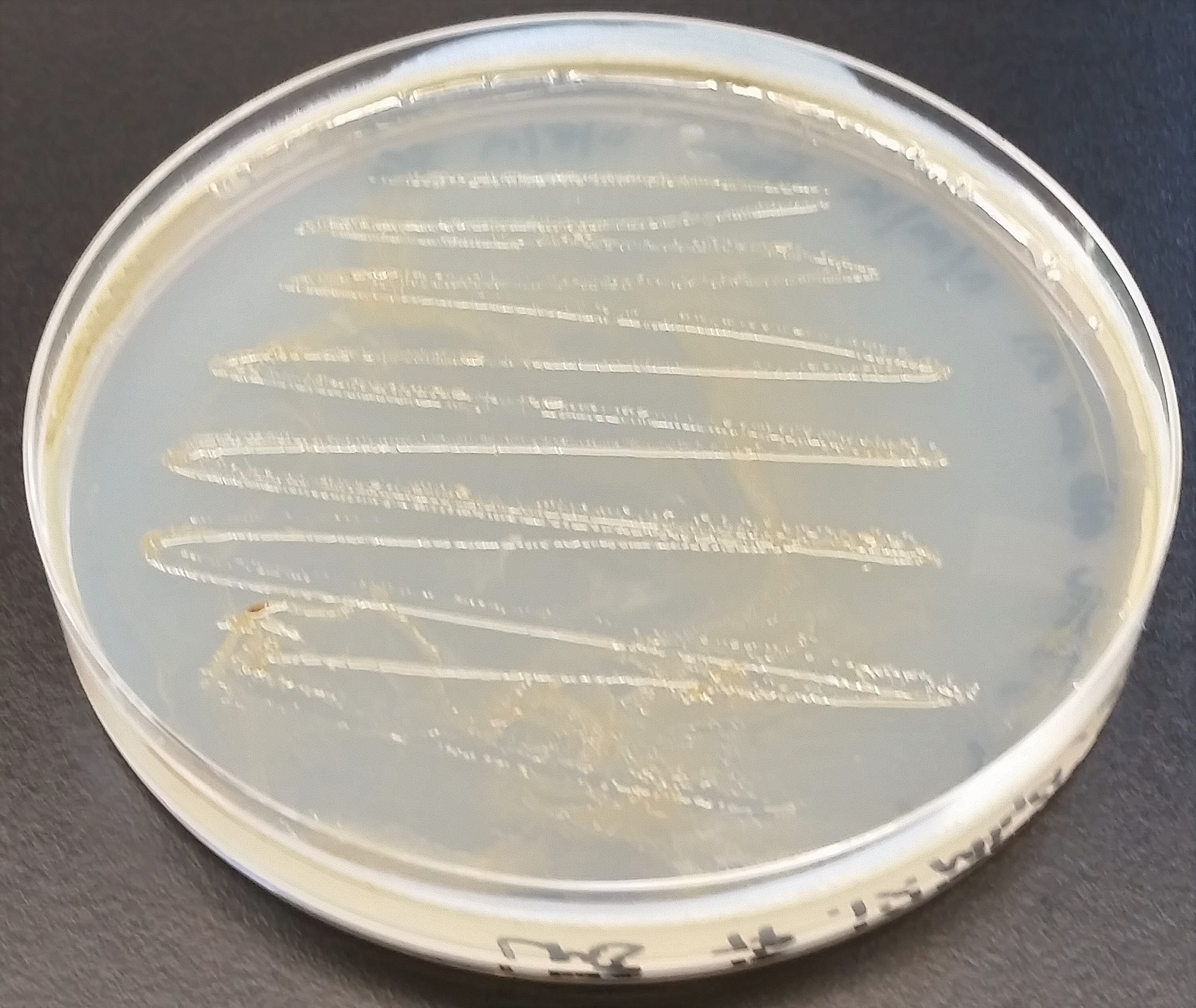

Supplement: Tests using Tris-Phosphate medium (TP) to see if hydrocarbons, aromatic compounds and polyhydroxyalkanoates can be used by the bacterium LMJ (Bacterium strain clone LIB091_C05_1243 variant 16S ribosomal RNA; GenBank Accession # MN633292.1) as the sole carbon source. — This file contains 23 images of TP (Tris-Phosphate) medium plates containing different alternative carbon sources. Bacterium LMJ was streaked on these chemical plates to test if LMJ can utilize these chemicals as the sole carbon source for energy and growth. 1% stocks of the following chemicals were tested: cyclohexyl chloride, phenanthrene, napthalene, benzoic acid, phenyl acetate. 2% (v/v) stocks of fresh and used car motor oil 10W30 were also tested. The doses used are given in mL in the file name. [file f1000research-9-27224-s0002.tgz › 10W30usedLMJ2mL.jpg]

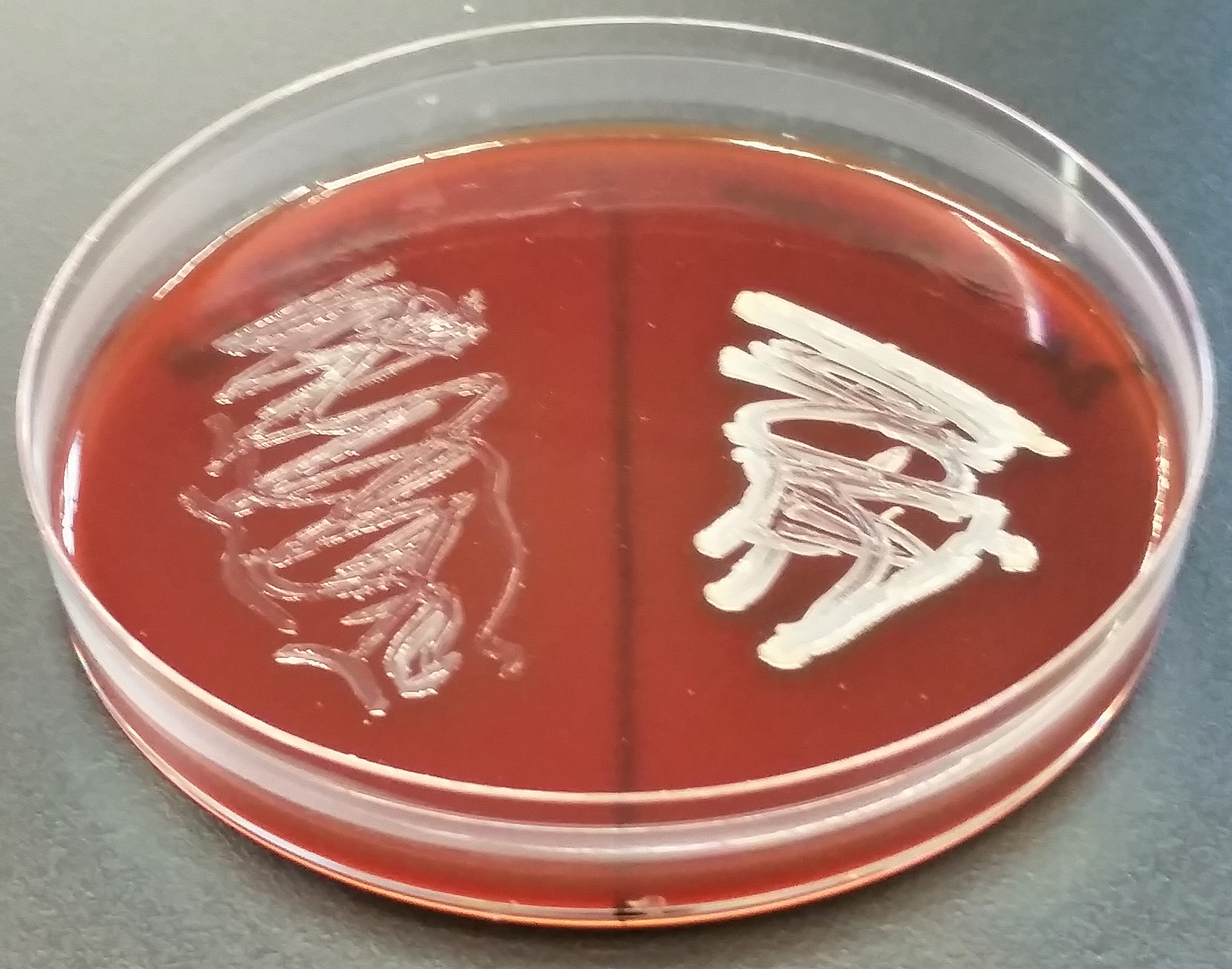

Supplement: Growth of bacterial strain LMJ (Bacterium strain clone LIB091_C05_1243 variant 16S ribosomal RNA; GenBank Accession # MN633292.1) and Staphylococcus aureus on Tryptic Soy Agar medium plates containing 5% sheep blood. — This file contains five images. We have included two figures showing the 24 hours-growth on the blood agar media plates, the growth of the bacterial strain LMJ is on the left on the blood agar plate and that of Staphylococcus aureus is on the right of the blood agar plate. Images were taken after 1 day of growth at 37C. S. aureus is beta-hemolytic while LMJ is gamma hemolytic. We have also included three additional images of LMJ growth on Tryptic Soy Agar medium for 24 hours, 48 hours and 72 hours at 37C to show that even with prolonged incubation at 37C, LMJ does not show complete or partial hemolysis on blood agar medium plates. Tryptic Soy Agar medium plates containing 5% sheep blood were purchased from Carolina Biological (Burlington, NC). [file f1000research-9-27224-s0004.tgz › Bloodagar24hrsgrowth.jpg]

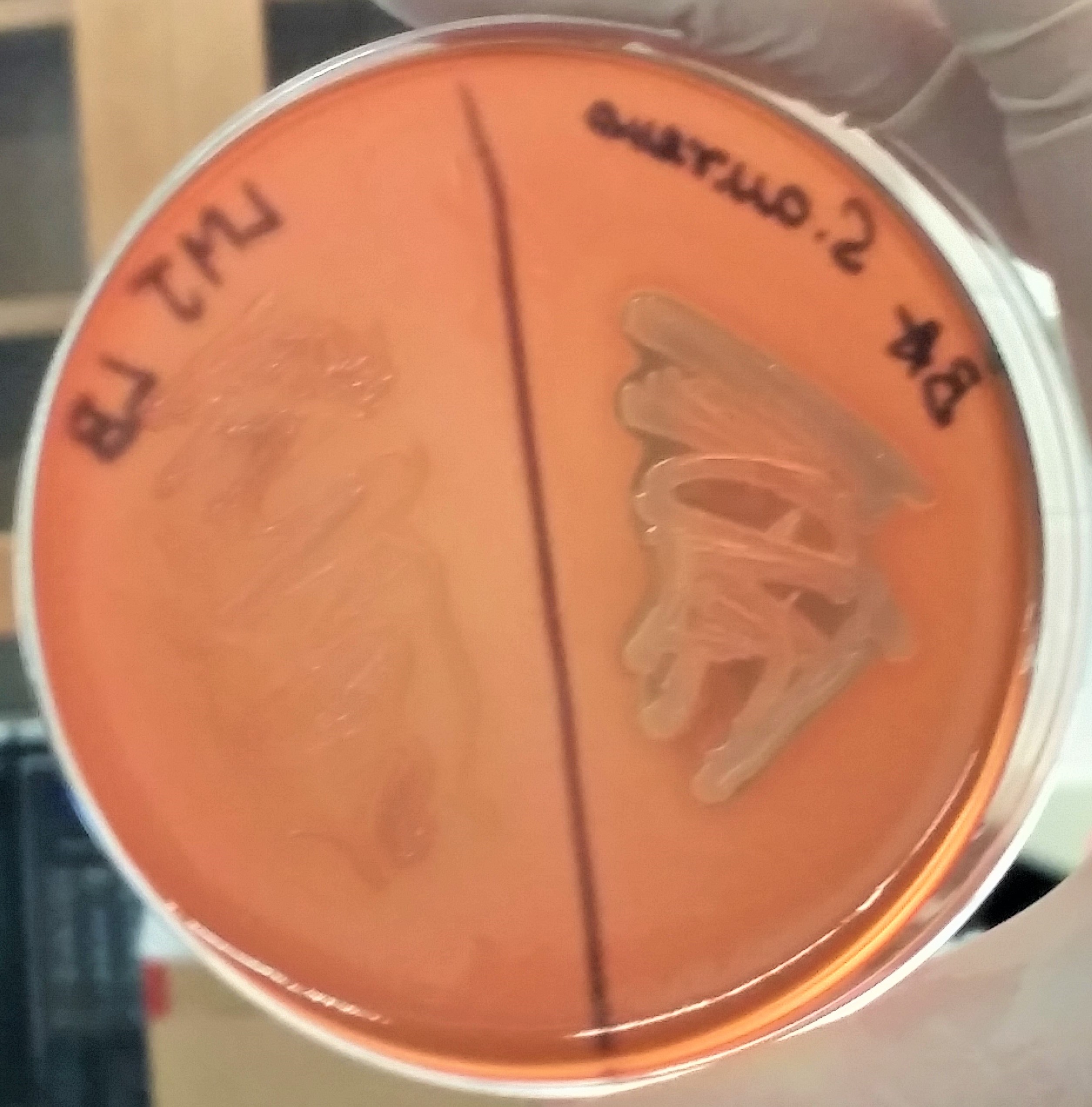

Supplement: Growth of bacterial strain LMJ (Bacterium strain clone LIB091_C05_1243 variant 16S ribosomal RNA; GenBank Accession # MN633292.1) and Staphylococcus aureus on Tryptic Soy Agar medium plates containing 5% sheep blood. — This file contains five images. We have included two figures showing the 24 hours-growth on the blood agar media plates, the growth of the bacterial strain LMJ is on the left on the blood agar plate and that of Staphylococcus aureus is on the right of the blood agar plate. Images were taken after 1 day of growth at 37C. S. aureus is beta-hemolytic while LMJ is gamma hemolytic. We have also included three additional images of LMJ growth on Tryptic Soy Agar medium for 24 hours, 48 hours and 72 hours at 37C to show that even with prolonged incubation at 37C, LMJ does not show complete or partial hemolysis on blood agar medium plates. Tryptic Soy Agar medium plates containing 5% sheep blood were purchased from Carolina Biological (Burlington, NC). [file f1000research-9-27224-s0004.tgz › Bloodagarlysisafter24hrs.jpg]

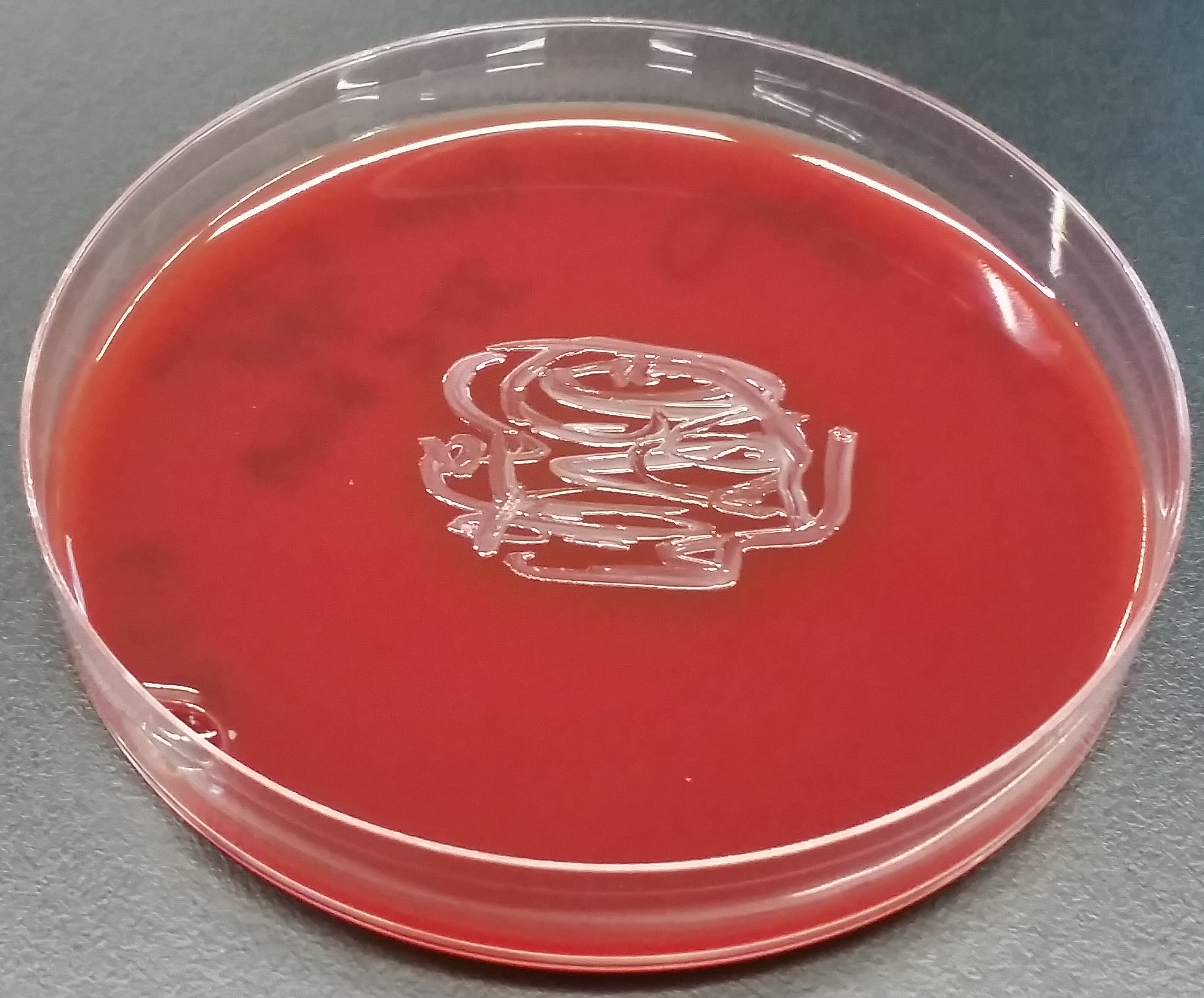

Supplement: Growth of bacterial strain LMJ (Bacterium strain clone LIB091_C05_1243 variant 16S ribosomal RNA; GenBank Accession # MN633292.1) and Staphylococcus aureus on Tryptic Soy Agar medium plates containing 5% sheep blood. — This file contains five images. We have included two figures showing the 24 hours-growth on the blood agar media plates, the growth of the bacterial strain LMJ is on the left on the blood agar plate and that of Staphylococcus aureus is on the right of the blood agar plate. Images were taken after 1 day of growth at 37C. S. aureus is beta-hemolytic while LMJ is gamma hemolytic. We have also included three additional images of LMJ growth on Tryptic Soy Agar medium for 24 hours, 48 hours and 72 hours at 37C to show that even with prolonged incubation at 37C, LMJ does not show complete or partial hemolysis on blood agar medium plates. Tryptic Soy Agar medium plates containing 5% sheep blood were purchased from Carolina Biological (Burlington, NC). [file f1000research-9-27224-s0004.tgz › BALMJ24hrs37C.jpg]

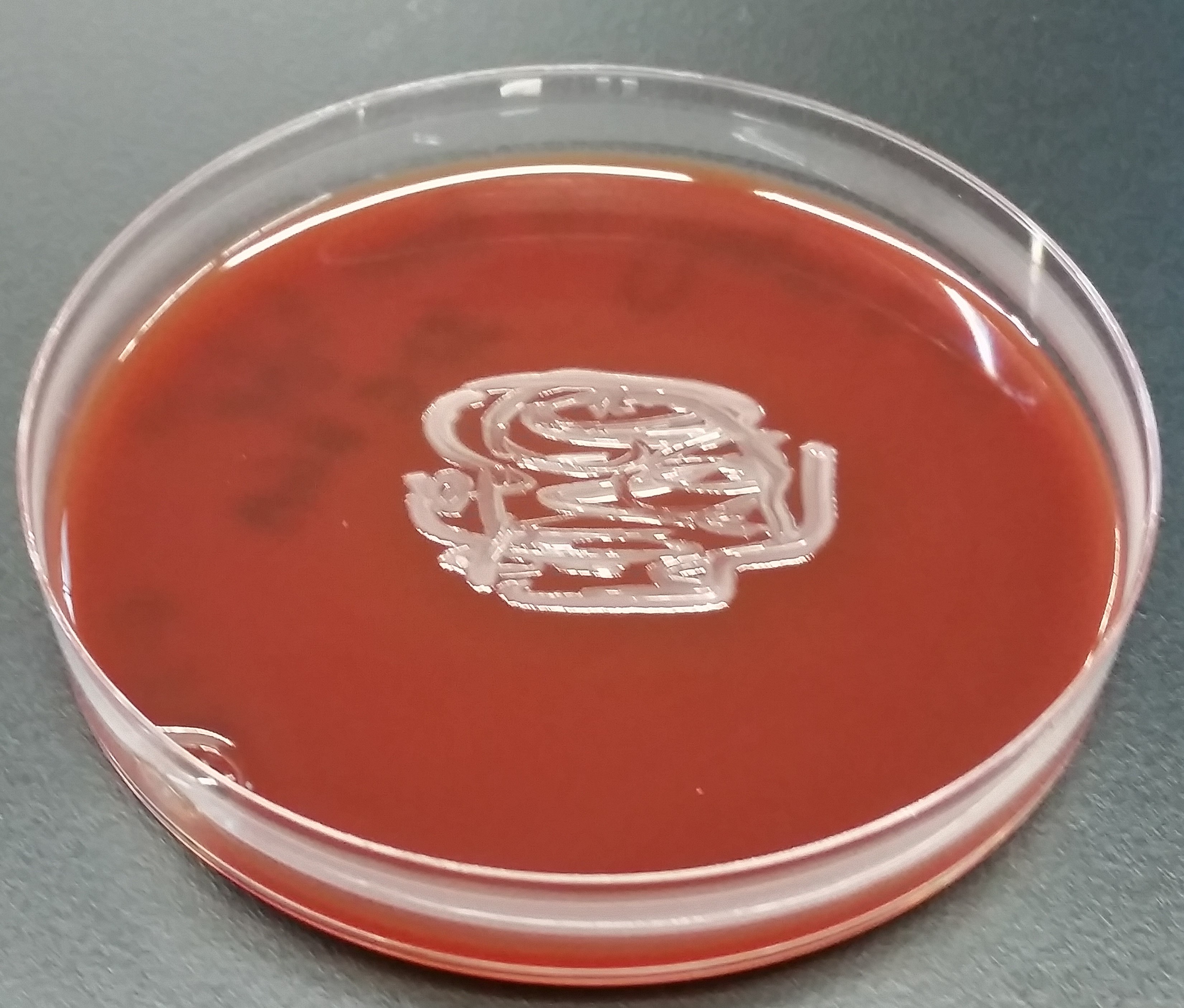

Supplement: Growth of bacterial strain LMJ (Bacterium strain clone LIB091_C05_1243 variant 16S ribosomal RNA; GenBank Accession # MN633292.1) and Staphylococcus aureus on Tryptic Soy Agar medium plates containing 5% sheep blood. — This file contains five images. We have included two figures showing the 24 hours-growth on the blood agar media plates, the growth of the bacterial strain LMJ is on the left on the blood agar plate and that of Staphylococcus aureus is on the right of the blood agar plate. Images were taken after 1 day of growth at 37C. S. aureus is beta-hemolytic while LMJ is gamma hemolytic. We have also included three additional images of LMJ growth on Tryptic Soy Agar medium for 24 hours, 48 hours and 72 hours at 37C to show that even with prolonged incubation at 37C, LMJ does not show complete or partial hemolysis on blood agar medium plates. Tryptic Soy Agar medium plates containing 5% sheep blood were purchased from Carolina Biological (Burlington, NC). [file f1000research-9-27224-s0004.tgz › BALMJ48hrs37C.jpg]

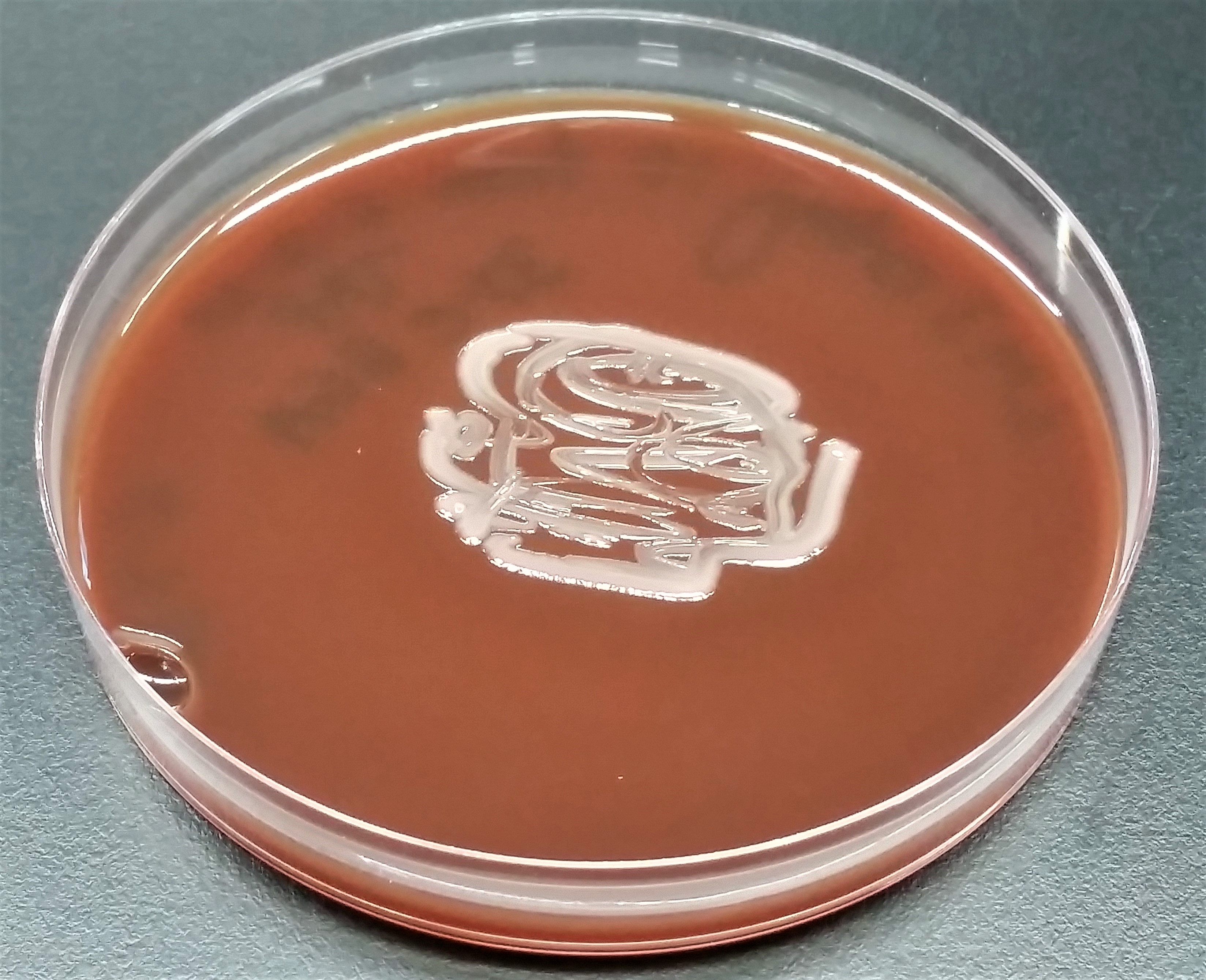

Supplement: Growth of bacterial strain LMJ (Bacterium strain clone LIB091_C05_1243 variant 16S ribosomal RNA; GenBank Accession # MN633292.1) and Staphylococcus aureus on Tryptic Soy Agar medium plates containing 5% sheep blood. — This file contains five images. We have included two figures showing the 24 hours-growth on the blood agar media plates, the growth of the bacterial strain LMJ is on the left on the blood agar plate and that of Staphylococcus aureus is on the right of the blood agar plate. Images were taken after 1 day of growth at 37C. S. aureus is beta-hemolytic while LMJ is gamma hemolytic. We have also included three additional images of LMJ growth on Tryptic Soy Agar medium for 24 hours, 48 hours and 72 hours at 37C to show that even with prolonged incubation at 37C, LMJ does not show complete or partial hemolysis on blood agar medium plates. Tryptic Soy Agar medium plates containing 5% sheep blood were purchased from Carolina Biological (Burlington, NC). [file f1000research-9-27224-s0004.tgz › BALMJ72hrs37C.jpg]

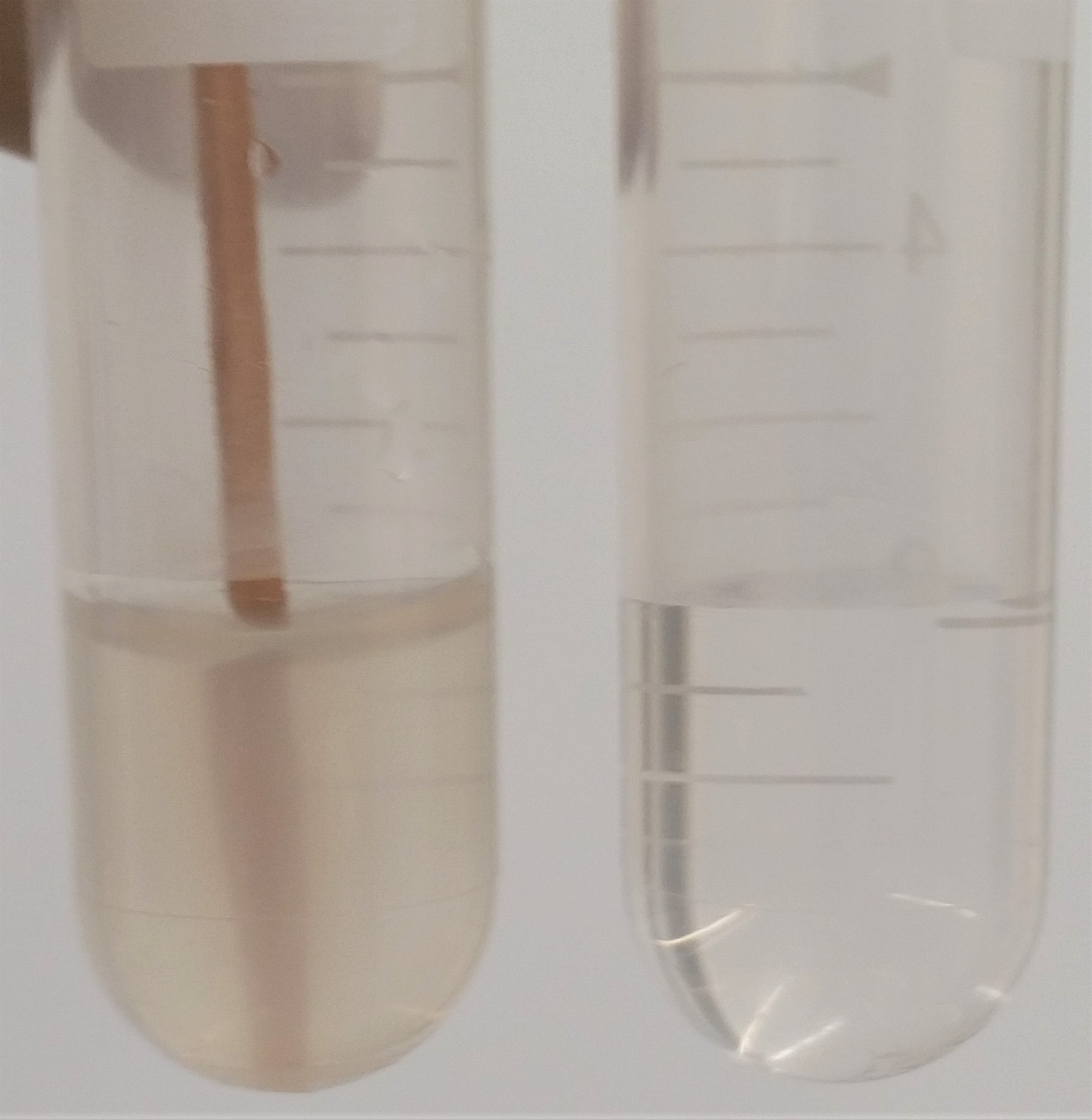

Supplement: TAP and LB liquid cultures of the bacterial strain LMJ (Bacterium strain clone LIB091_C05_1243 variant 16S ribosomal RNA; GenBank Accession # MN633292.1) grown for 4 days and Gram stains of LMJ from these liquid cultures. Growth of LMJ on TAP-agar from a 96 hours-grown TAP liquid culture is also sho — This file contains 11 images. Bacterial strain LMJ was grown in liquid TAP and liquid LB medium for 4 days (96 hours) in an incubator shaker, shaking at 150 rpm at 37C. Images were taken every 24 hours to monitor the turbidity/growth of these liquid cultures over 96 hours. Gram stains were performed on the 24 hours-grown liquid TAP and LB cultures. There were cell debris, membrane fragments, some small rods and round shaped cells were visible after gram staining. 96 hours liquid TAP culture was plated on a TAP-agar medium plate. LMJ grew back within 4 days at room temperature. This result shows that although gram stain could not detect many intact cells in the liquid culture, cells were not dead. LMJ could not form a biofilm in shaking cultures properly and this induced cell lysis in liquid cultures. It can form biofilms on TAP-agar when plated from the liquid TAP culture. [file f1000research-9-27224-s0005.tgz › 24hrsgrowthTAP.jpg]

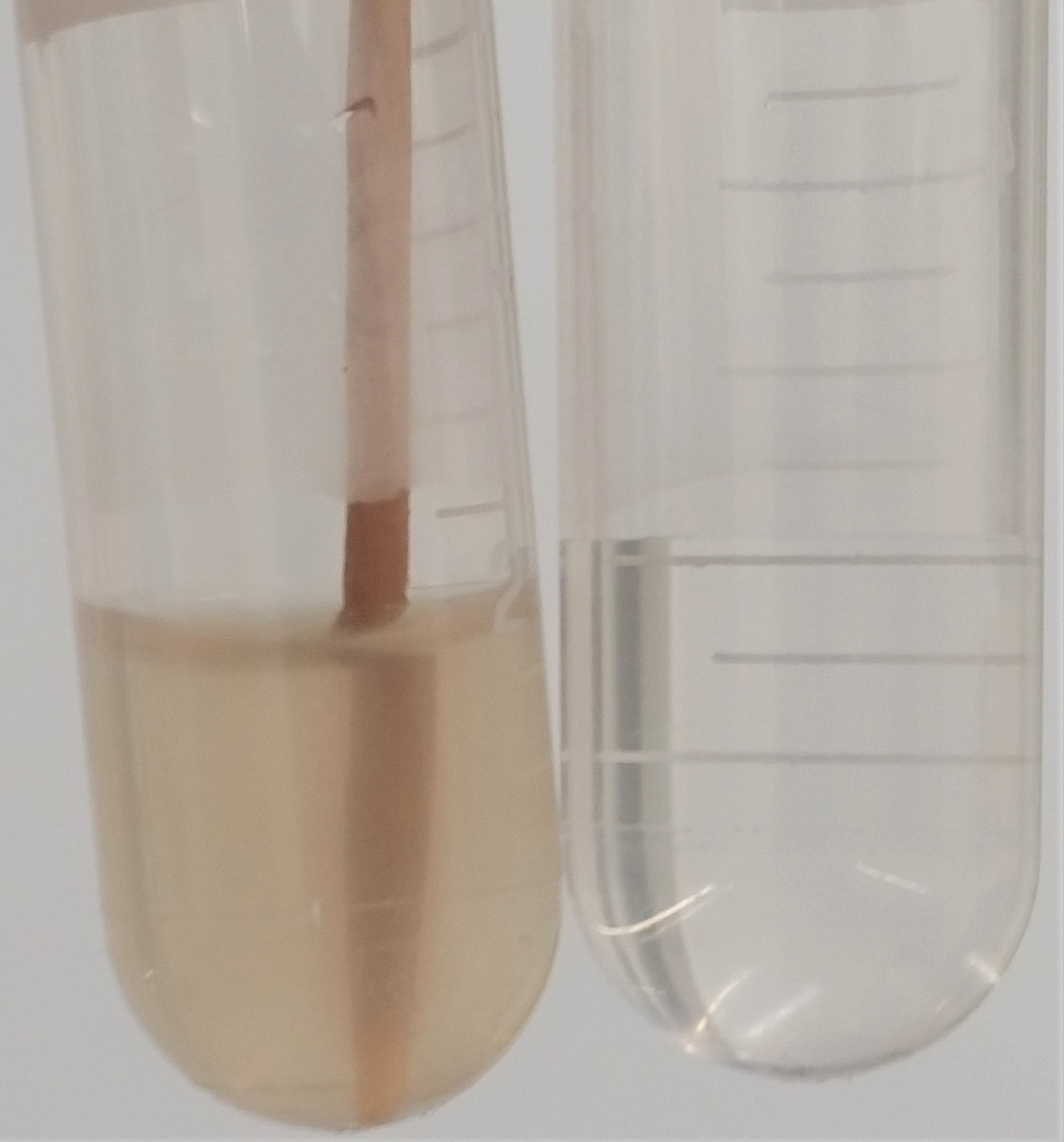

Supplement: TAP and LB liquid cultures of the bacterial strain LMJ (Bacterium strain clone LIB091_C05_1243 variant 16S ribosomal RNA; GenBank Accession # MN633292.1) grown for 4 days and Gram stains of LMJ from these liquid cultures. Growth of LMJ on TAP-agar from a 96 hours-grown TAP liquid culture is also sho — This file contains 11 images. Bacterial strain LMJ was grown in liquid TAP and liquid LB medium for 4 days (96 hours) in an incubator shaker, shaking at 150 rpm at 37C. Images were taken every 24 hours to monitor the turbidity/growth of these liquid cultures over 96 hours. Gram stains were performed on the 24 hours-grown liquid TAP and LB cultures. There were cell debris, membrane fragments, some small rods and round shaped cells were visible after gram staining. 96 hours liquid TAP culture was plated on a TAP-agar medium plate. LMJ grew back within 4 days at room temperature. This result shows that although gram stain could not detect many intact cells in the liquid culture, cells were not dead. LMJ could not form a biofilm in shaking cultures properly and this induced cell lysis in liquid cultures. It can form biofilms on TAP-agar when plated from the liquid TAP culture. [file f1000research-9-27224-s0005.tgz › 48hrsgrowthTAP.jpg]

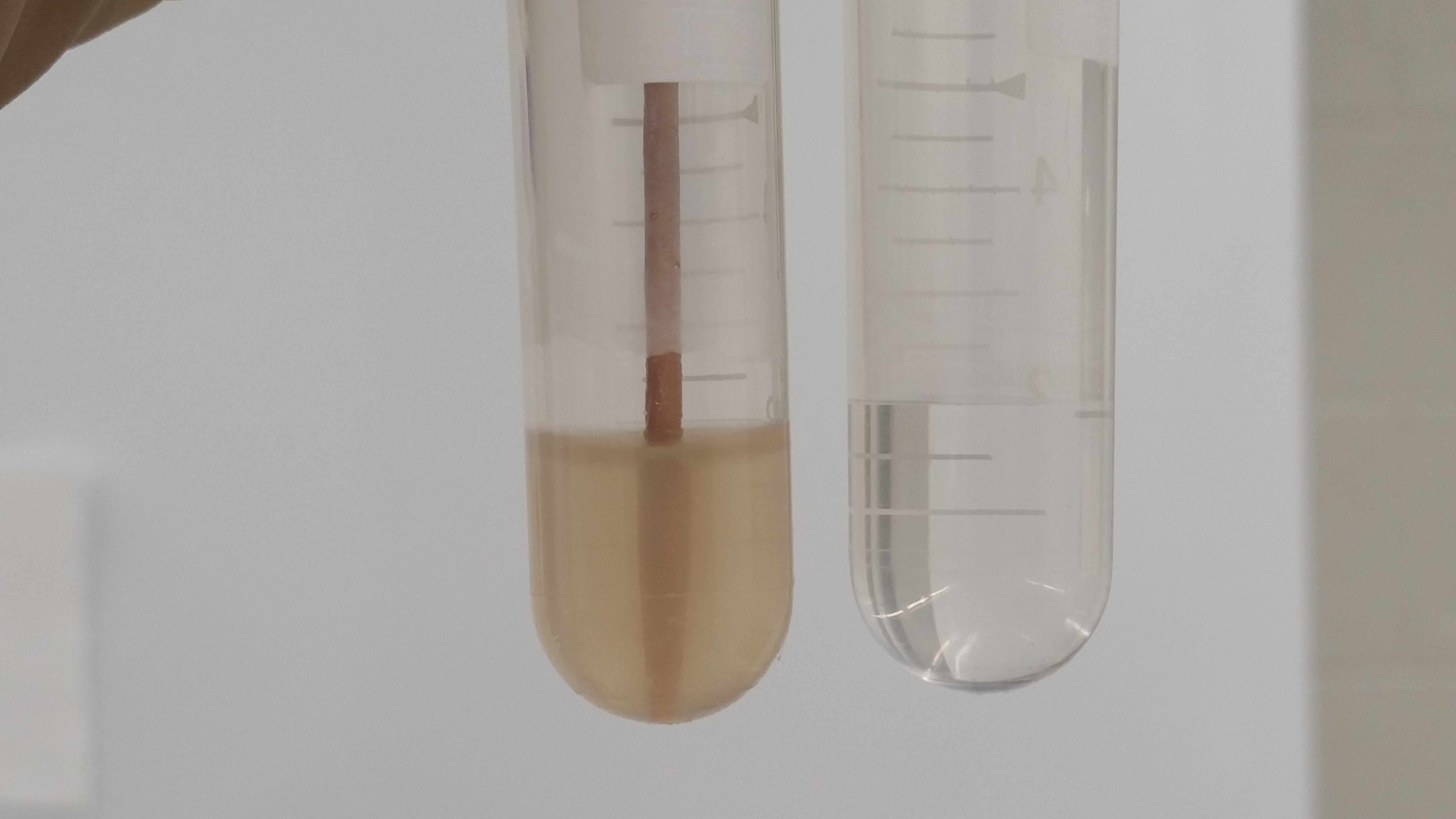

Supplement: TAP and LB liquid cultures of the bacterial strain LMJ (Bacterium strain clone LIB091_C05_1243 variant 16S ribosomal RNA; GenBank Accession # MN633292.1) grown for 4 days and Gram stains of LMJ from these liquid cultures. Growth of LMJ on TAP-agar from a 96 hours-grown TAP liquid culture is also sho — This file contains 11 images. Bacterial strain LMJ was grown in liquid TAP and liquid LB medium for 4 days (96 hours) in an incubator shaker, shaking at 150 rpm at 37C. Images were taken every 24 hours to monitor the turbidity/growth of these liquid cultures over 96 hours. Gram stains were performed on the 24 hours-grown liquid TAP and LB cultures. There were cell debris, membrane fragments, some small rods and round shaped cells were visible after gram staining. 96 hours liquid TAP culture was plated on a TAP-agar medium plate. LMJ grew back within 4 days at room temperature. This result shows that although gram stain could not detect many intact cells in the liquid culture, cells were not dead. LMJ could not form a biofilm in shaking cultures properly and this induced cell lysis in liquid cultures. It can form biofilms on TAP-agar when plated from the liquid TAP culture. [file f1000research-9-27224-s0005.tgz › 72hrsgrowthTAP.jpg]

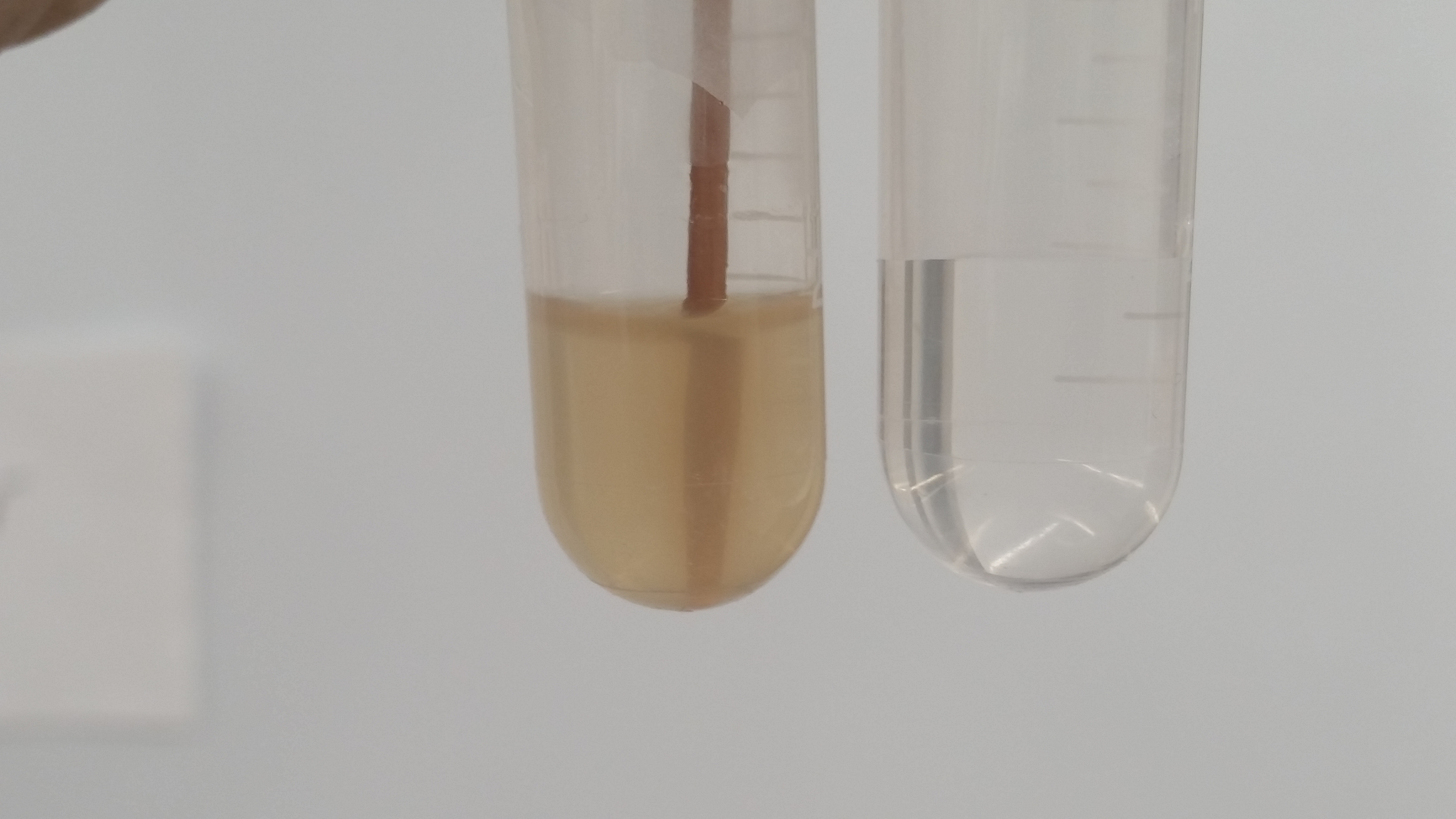

Supplement: TAP and LB liquid cultures of the bacterial strain LMJ (Bacterium strain clone LIB091_C05_1243 variant 16S ribosomal RNA; GenBank Accession # MN633292.1) grown for 4 days and Gram stains of LMJ from these liquid cultures. Growth of LMJ on TAP-agar from a 96 hours-grown TAP liquid culture is also sho — This file contains 11 images. Bacterial strain LMJ was grown in liquid TAP and liquid LB medium for 4 days (96 hours) in an incubator shaker, shaking at 150 rpm at 37C. Images were taken every 24 hours to monitor the turbidity/growth of these liquid cultures over 96 hours. Gram stains were performed on the 24 hours-grown liquid TAP and LB cultures. There were cell debris, membrane fragments, some small rods and round shaped cells were visible after gram staining. 96 hours liquid TAP culture was plated on a TAP-agar medium plate. LMJ grew back within 4 days at room temperature. This result shows that although gram stain could not detect many intact cells in the liquid culture, cells were not dead. LMJ could not form a biofilm in shaking cultures properly and this induced cell lysis in liquid cultures. It can form biofilms on TAP-agar when plated from the liquid TAP culture. [file f1000research-9-27224-s0005.tgz › 96hrsgrowth.jpg]

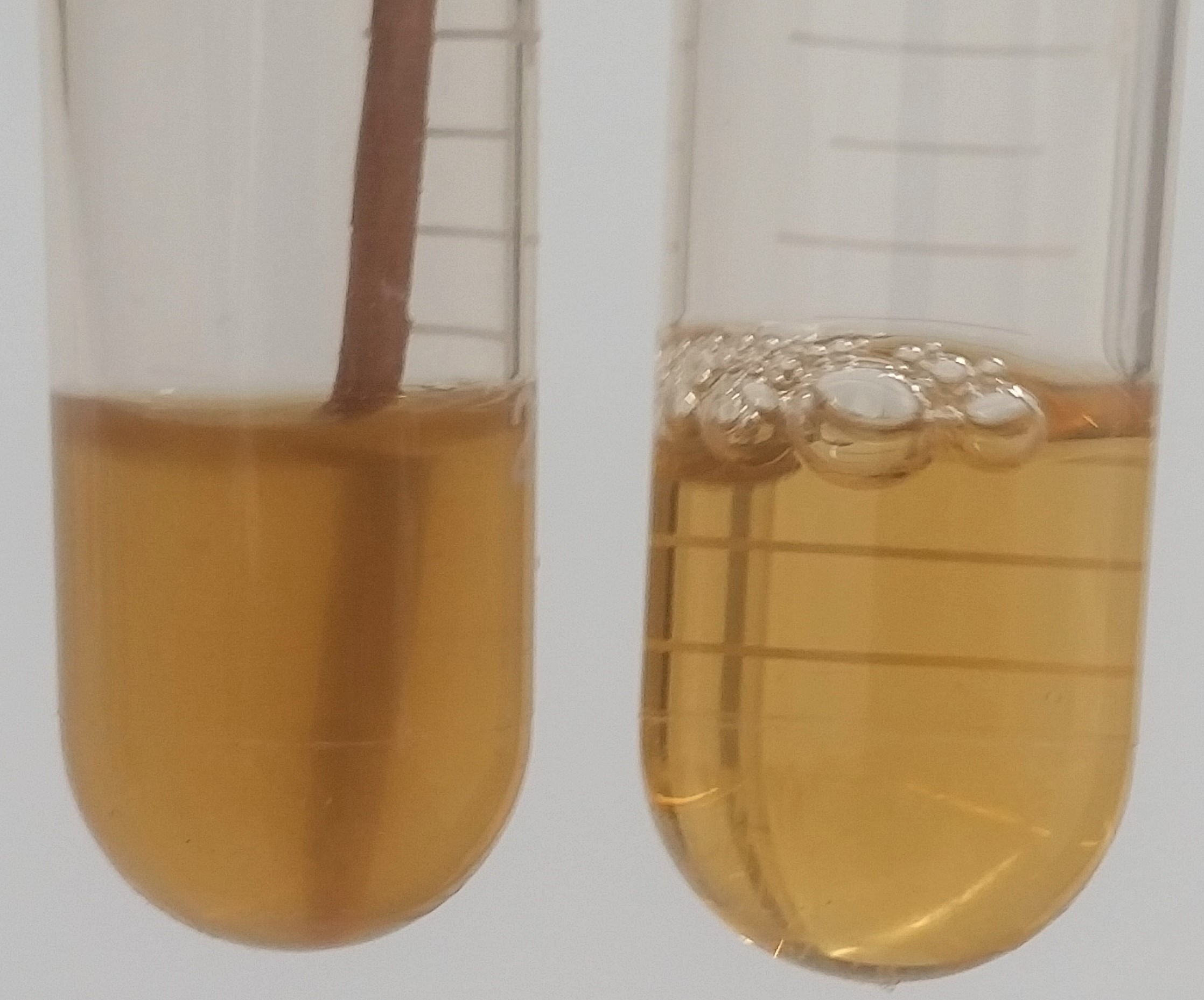

Supplement: TAP and LB liquid cultures of the bacterial strain LMJ (Bacterium strain clone LIB091_C05_1243 variant 16S ribosomal RNA; GenBank Accession # MN633292.1) grown for 4 days and Gram stains of LMJ from these liquid cultures. Growth of LMJ on TAP-agar from a 96 hours-grown TAP liquid culture is also sho — This file contains 11 images. Bacterial strain LMJ was grown in liquid TAP and liquid LB medium for 4 days (96 hours) in an incubator shaker, shaking at 150 rpm at 37C. Images were taken every 24 hours to monitor the turbidity/growth of these liquid cultures over 96 hours. Gram stains were performed on the 24 hours-grown liquid TAP and LB cultures. There were cell debris, membrane fragments, some small rods and round shaped cells were visible after gram staining. 96 hours liquid TAP culture was plated on a TAP-agar medium plate. LMJ grew back within 4 days at room temperature. This result shows that although gram stain could not detect many intact cells in the liquid culture, cells were not dead. LMJ could not form a biofilm in shaking cultures properly and this induced cell lysis in liquid cultures. It can form biofilms on TAP-agar when plated from the liquid TAP culture. [file f1000research-9-27224-s0005.tgz › 24hoursgrowthLB.jpg]

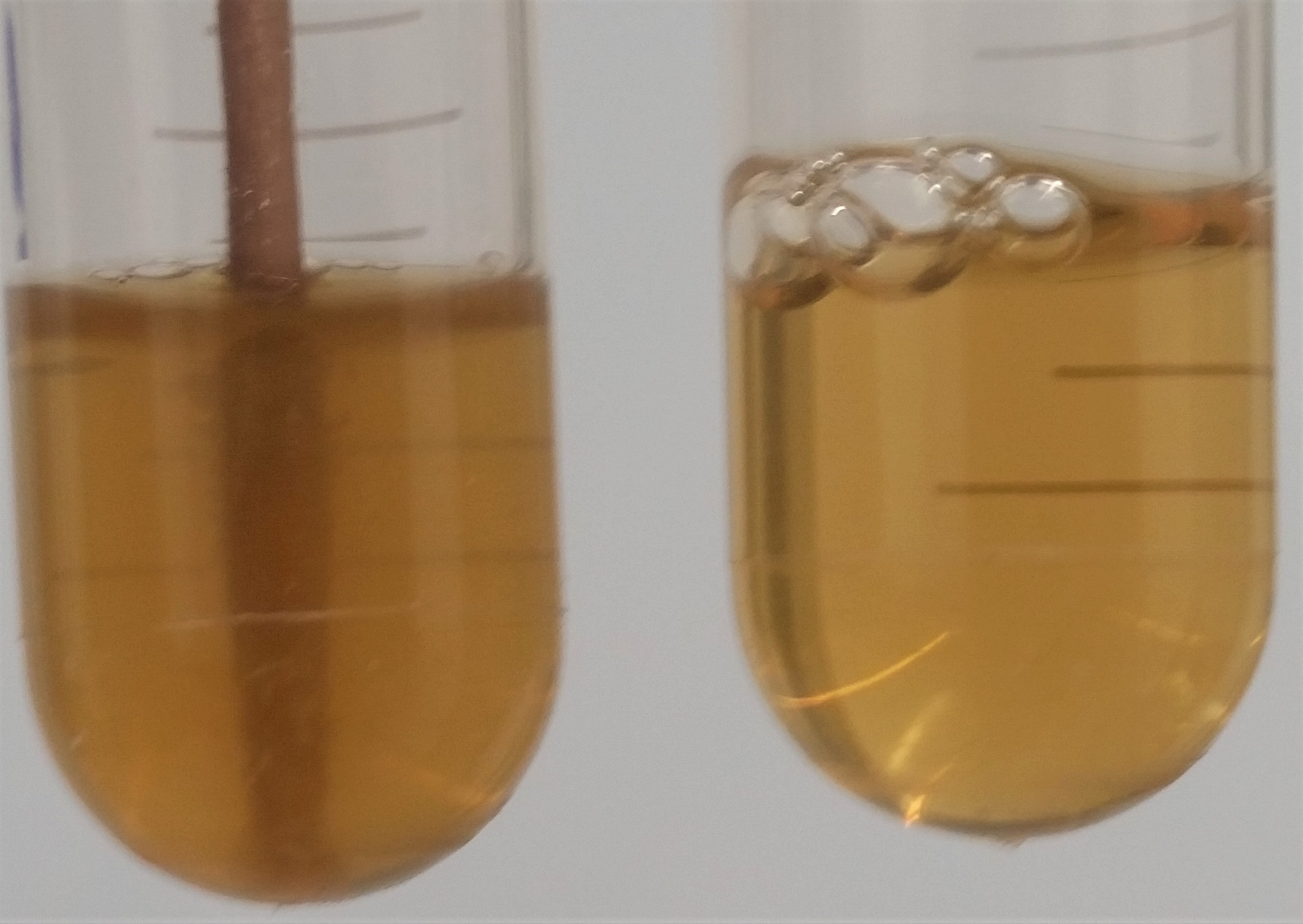

Supplement: TAP and LB liquid cultures of the bacterial strain LMJ (Bacterium strain clone LIB091_C05_1243 variant 16S ribosomal RNA; GenBank Accession # MN633292.1) grown for 4 days and Gram stains of LMJ from these liquid cultures. Growth of LMJ on TAP-agar from a 96 hours-grown TAP liquid culture is also sho — This file contains 11 images. Bacterial strain LMJ was grown in liquid TAP and liquid LB medium for 4 days (96 hours) in an incubator shaker, shaking at 150 rpm at 37C. Images were taken every 24 hours to monitor the turbidity/growth of these liquid cultures over 96 hours. Gram stains were performed on the 24 hours-grown liquid TAP and LB cultures. There were cell debris, membrane fragments, some small rods and round shaped cells were visible after gram staining. 96 hours liquid TAP culture was plated on a TAP-agar medium plate. LMJ grew back within 4 days at room temperature. This result shows that although gram stain could not detect many intact cells in the liquid culture, cells were not dead. LMJ could not form a biofilm in shaking cultures properly and this induced cell lysis in liquid cultures. It can form biofilms on TAP-agar when plated from the liquid TAP culture. [file f1000research-9-27224-s0005.tgz › 48hrsgrowthLB.jpg]

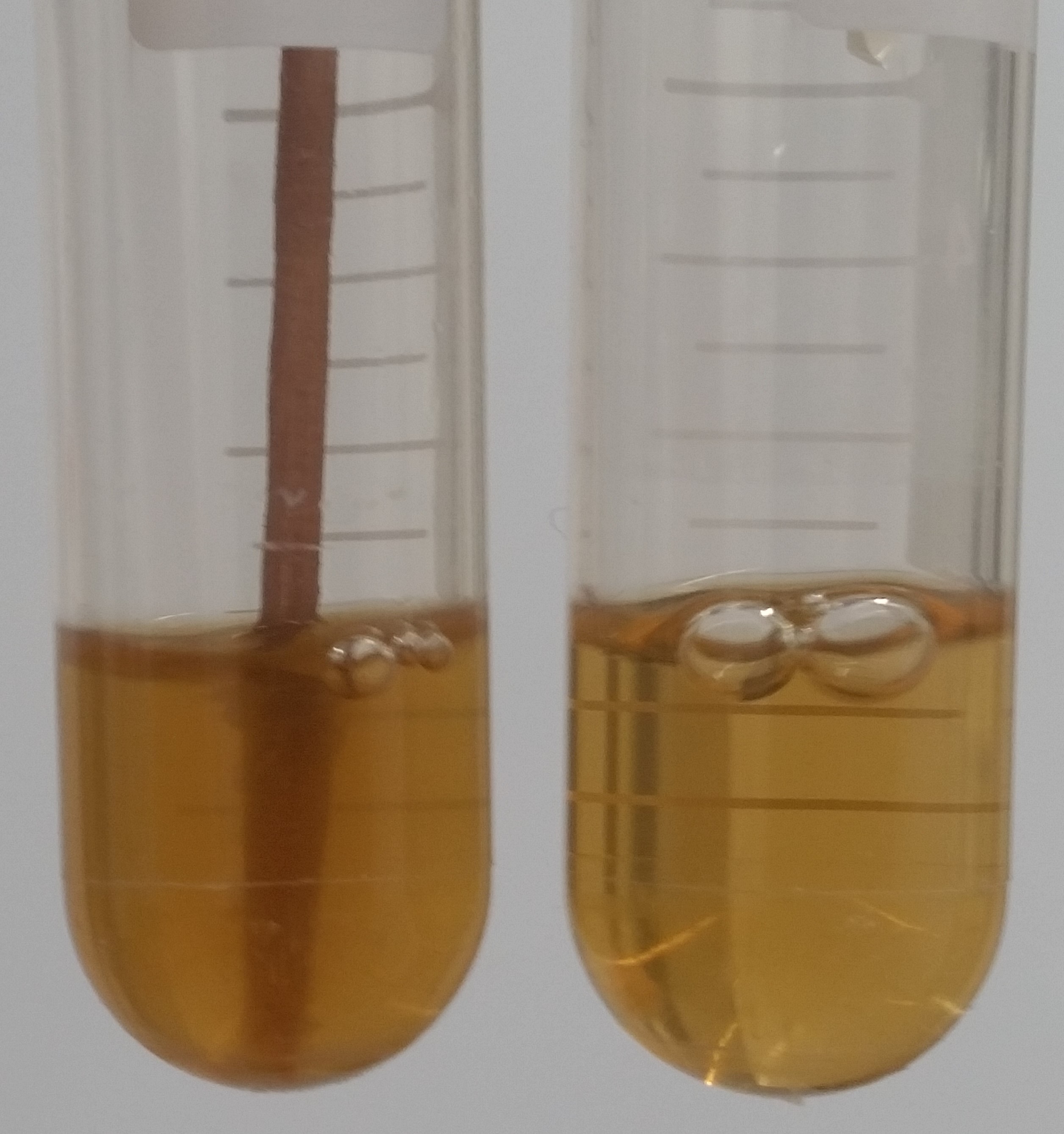

Supplement: TAP and LB liquid cultures of the bacterial strain LMJ (Bacterium strain clone LIB091_C05_1243 variant 16S ribosomal RNA; GenBank Accession # MN633292.1) grown for 4 days and Gram stains of LMJ from these liquid cultures. Growth of LMJ on TAP-agar from a 96 hours-grown TAP liquid culture is also sho — This file contains 11 images. Bacterial strain LMJ was grown in liquid TAP and liquid LB medium for 4 days (96 hours) in an incubator shaker, shaking at 150 rpm at 37C. Images were taken every 24 hours to monitor the turbidity/growth of these liquid cultures over 96 hours. Gram stains were performed on the 24 hours-grown liquid TAP and LB cultures. There were cell debris, membrane fragments, some small rods and round shaped cells were visible after gram staining. 96 hours liquid TAP culture was plated on a TAP-agar medium plate. LMJ grew back within 4 days at room temperature. This result shows that although gram stain could not detect many intact cells in the liquid culture, cells were not dead. LMJ could not form a biofilm in shaking cultures properly and this induced cell lysis in liquid cultures. It can form biofilms on TAP-agar when plated from the liquid TAP culture. [file f1000research-9-27224-s0005.tgz › 72hrsgrowthLB.jpg]

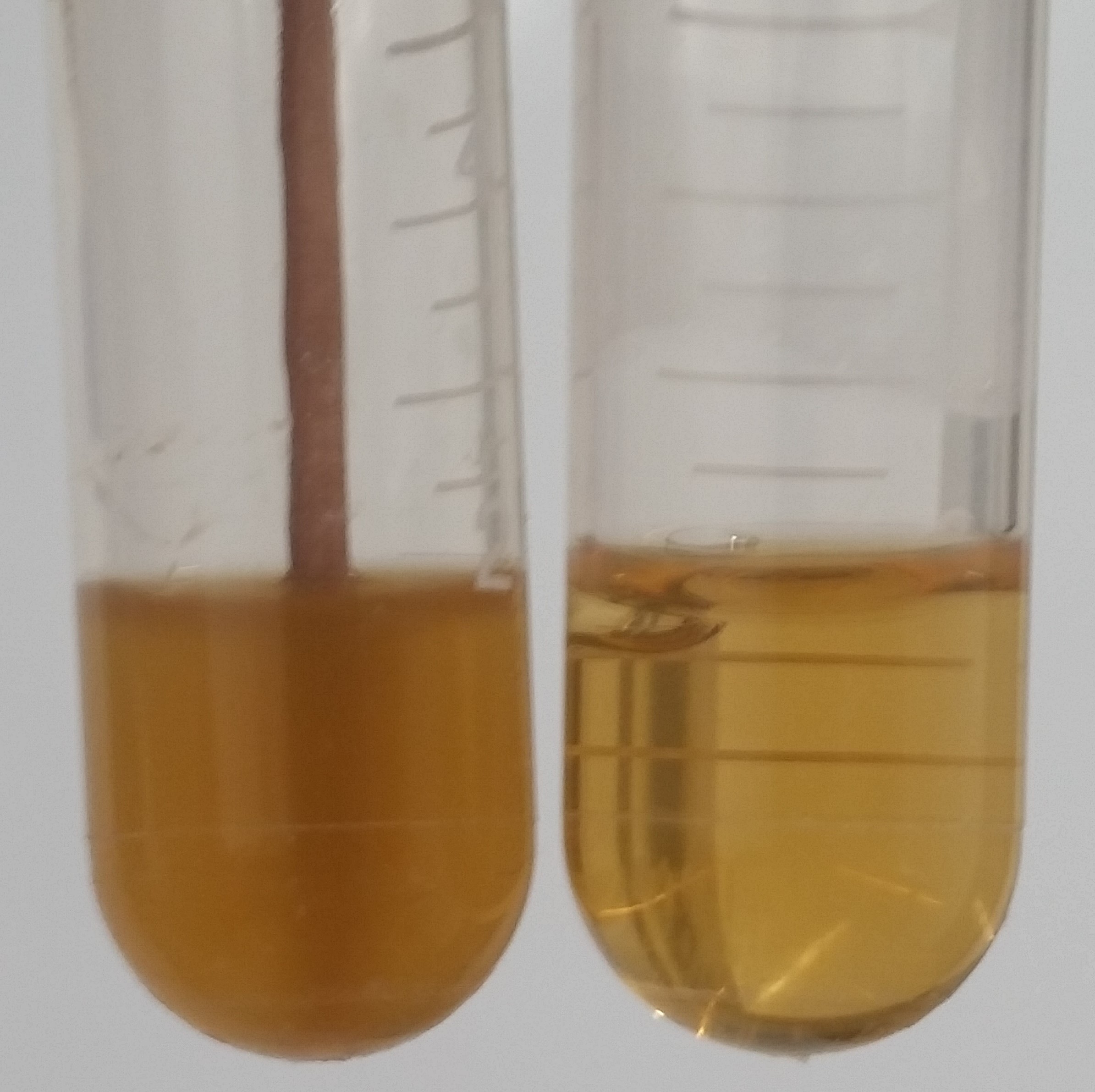

Supplement: TAP and LB liquid cultures of the bacterial strain LMJ (Bacterium strain clone LIB091_C05_1243 variant 16S ribosomal RNA; GenBank Accession # MN633292.1) grown for 4 days and Gram stains of LMJ from these liquid cultures. Growth of LMJ on TAP-agar from a 96 hours-grown TAP liquid culture is also sho — This file contains 11 images. Bacterial strain LMJ was grown in liquid TAP and liquid LB medium for 4 days (96 hours) in an incubator shaker, shaking at 150 rpm at 37C. Images were taken every 24 hours to monitor the turbidity/growth of these liquid cultures over 96 hours. Gram stains were performed on the 24 hours-grown liquid TAP and LB cultures. There were cell debris, membrane fragments, some small rods and round shaped cells were visible after gram staining. 96 hours liquid TAP culture was plated on a TAP-agar medium plate. LMJ grew back within 4 days at room temperature. This result shows that although gram stain could not detect many intact cells in the liquid culture, cells were not dead. LMJ could not form a biofilm in shaking cultures properly and this induced cell lysis in liquid cultures. It can form biofilms on TAP-agar when plated from the liquid TAP culture. [file f1000research-9-27224-s0005.tgz › 96hrsgrowthLB.jpg]

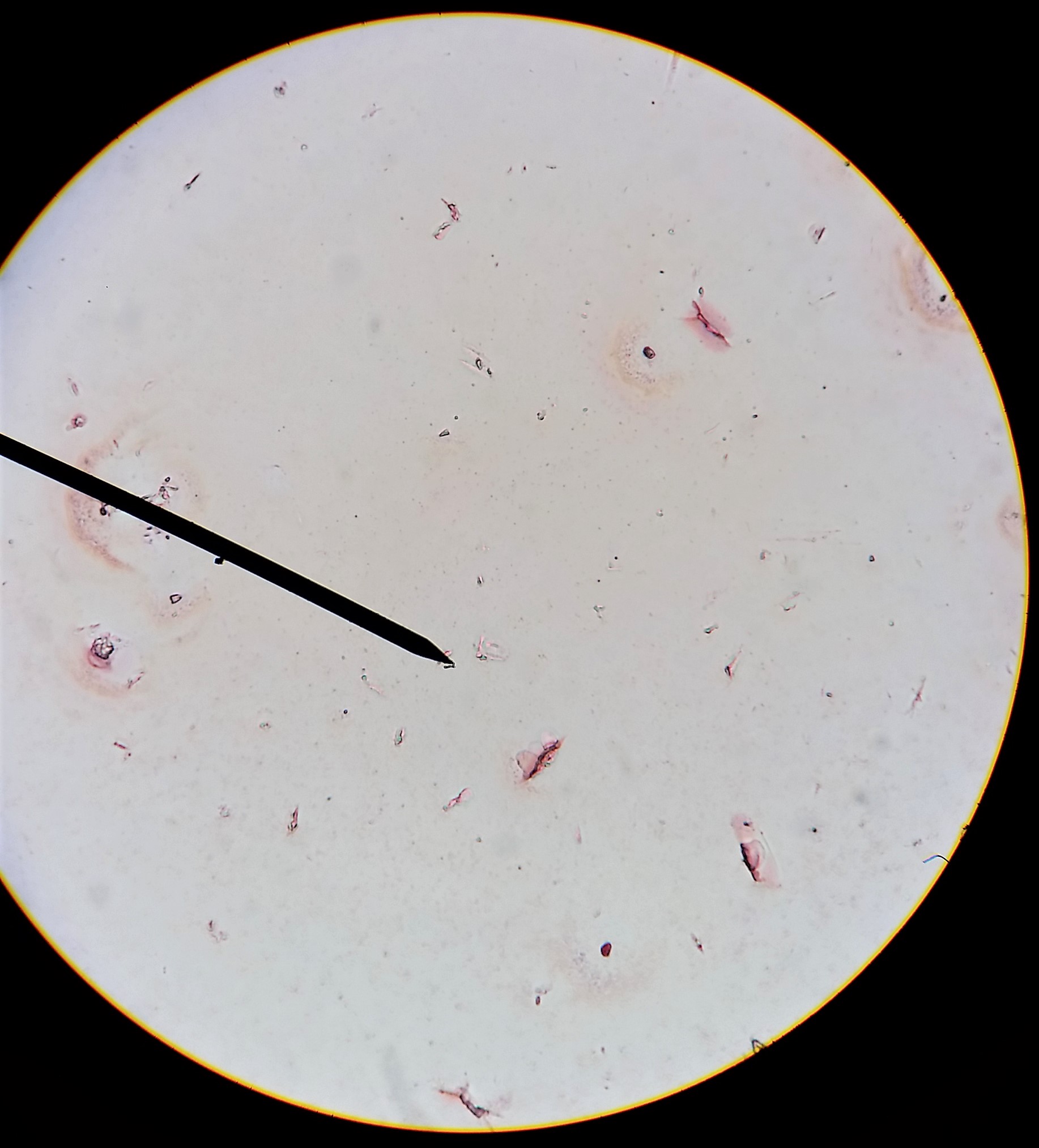

Supplement: TAP and LB liquid cultures of the bacterial strain LMJ (Bacterium strain clone LIB091_C05_1243 variant 16S ribosomal RNA; GenBank Accession # MN633292.1) grown for 4 days and Gram stains of LMJ from these liquid cultures. Growth of LMJ on TAP-agar from a 96 hours-grown TAP liquid culture is also sho — This file contains 11 images. Bacterial strain LMJ was grown in liquid TAP and liquid LB medium for 4 days (96 hours) in an incubator shaker, shaking at 150 rpm at 37C. Images were taken every 24 hours to monitor the turbidity/growth of these liquid cultures over 96 hours. Gram stains were performed on the 24 hours-grown liquid TAP and LB cultures. There were cell debris, membrane fragments, some small rods and round shaped cells were visible after gram staining. 96 hours liquid TAP culture was plated on a TAP-agar medium plate. LMJ grew back within 4 days at room temperature. This result shows that although gram stain could not detect many intact cells in the liquid culture, cells were not dead. LMJ could not form a biofilm in shaking cultures properly and this induced cell lysis in liquid cultures. It can form biofilms on TAP-agar when plated from the liquid TAP culture. [file f1000research-9-27224-s0005.tgz › LMJTAPcelldebrisaftergramstaining.jpg]

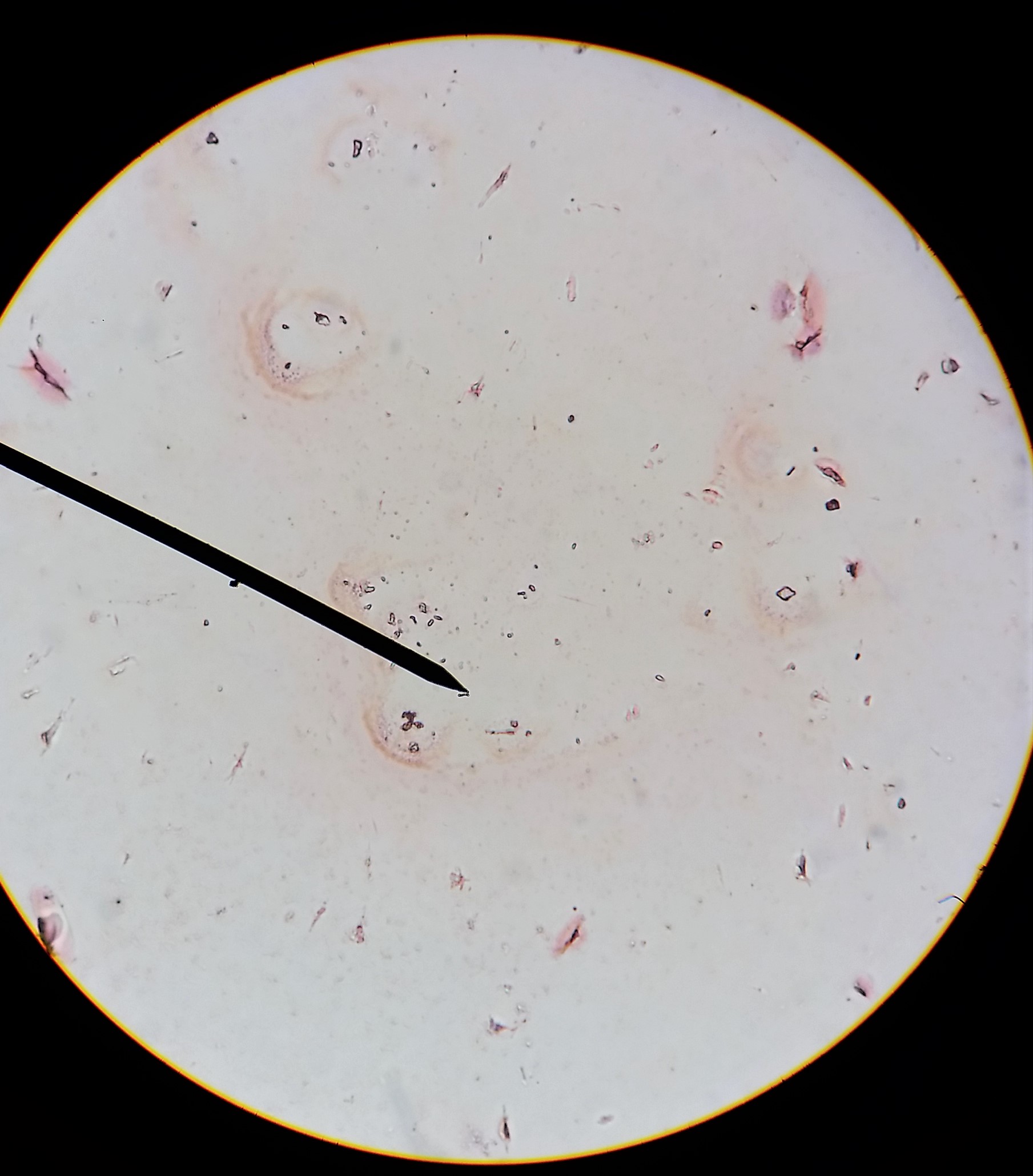

Supplement: TAP and LB liquid cultures of the bacterial strain LMJ (Bacterium strain clone LIB091_C05_1243 variant 16S ribosomal RNA; GenBank Accession # MN633292.1) grown for 4 days and Gram stains of LMJ from these liquid cultures. Growth of LMJ on TAP-agar from a 96 hours-grown TAP liquid culture is also sho — This file contains 11 images. Bacterial strain LMJ was grown in liquid TAP and liquid LB medium for 4 days (96 hours) in an incubator shaker, shaking at 150 rpm at 37C. Images were taken every 24 hours to monitor the turbidity/growth of these liquid cultures over 96 hours. Gram stains were performed on the 24 hours-grown liquid TAP and LB cultures. There were cell debris, membrane fragments, some small rods and round shaped cells were visible after gram staining. 96 hours liquid TAP culture was plated on a TAP-agar medium plate. LMJ grew back within 4 days at room temperature. This result shows that although gram stain could not detect many intact cells in the liquid culture, cells were not dead. LMJ could not form a biofilm in shaking cultures properly and this induced cell lysis in liquid cultures. It can form biofilms on TAP-agar when plated from the liquid TAP culture. [file f1000research-9-27224-s0005.tgz › LMJLBcelldebrisafterGramstaining.jpg]

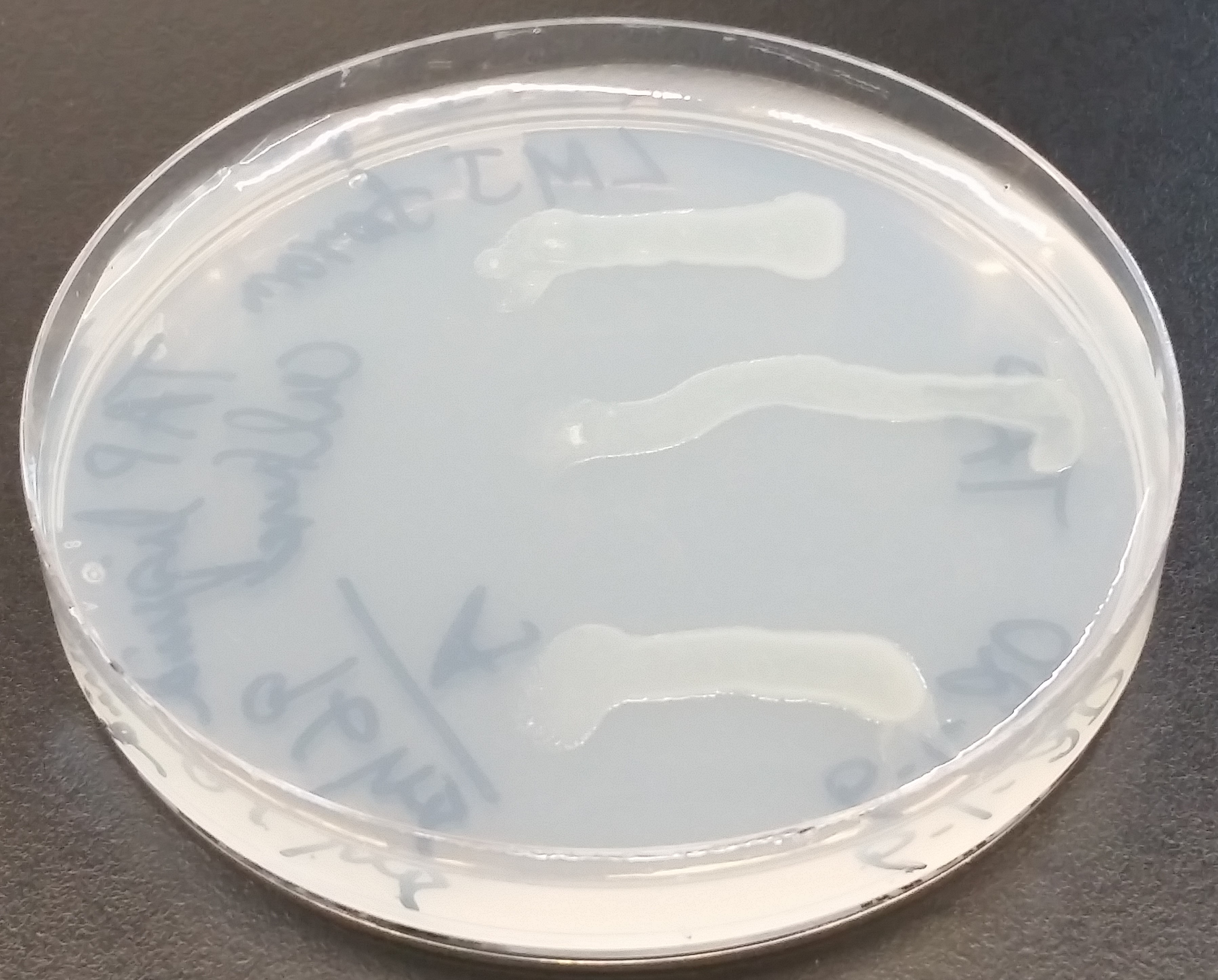

Supplement: TAP and LB liquid cultures of the bacterial strain LMJ (Bacterium strain clone LIB091_C05_1243 variant 16S ribosomal RNA; GenBank Accession # MN633292.1) grown for 4 days and Gram stains of LMJ from these liquid cultures. Growth of LMJ on TAP-agar from a 96 hours-grown TAP liquid culture is also sho — This file contains 11 images. Bacterial strain LMJ was grown in liquid TAP and liquid LB medium for 4 days (96 hours) in an incubator shaker, shaking at 150 rpm at 37C. Images were taken every 24 hours to monitor the turbidity/growth of these liquid cultures over 96 hours. Gram stains were performed on the 24 hours-grown liquid TAP and LB cultures. There were cell debris, membrane fragments, some small rods and round shaped cells were visible after gram staining. 96 hours liquid TAP culture was plated on a TAP-agar medium plate. LMJ grew back within 4 days at room temperature. This result shows that although gram stain could not detect many intact cells in the liquid culture, cells were not dead. LMJ could not form a biofilm in shaking cultures properly and this induced cell lysis in liquid cultures. It can form biofilms on TAP-agar when plated from the liquid TAP culture. [file f1000research-9-27224-s0005.tgz › 96hrsliquidTAPculturetoTAPagar.jpg]
