# Supplementary material for: Isolation and characterization of a novel bacterial strain from a Tris-Acetate-Phosphate agar medium plate of the green micro-alga Chlamydomonas reinhardtii that can utilize common environmental pollutants as a carbon source
Source: F1000Res. 2020 Jun 29;9:656. [Version 1] doi: 10.12688/f1000research.24680.1 (PMC7425125; doi:10.12688/f1000research.24680.1)
Supplement: Peer review history

# Peer review: Reviewer response for version 1

**DOI:** 10.5256/f1000research.27224.r69021

**Published:** 2020-08-19

**Authors:** Juan Campos Guillén

**Affiliations:** 1 Facultad de Química, Universidad Autónoma de Querétaro, Santiago de Querétaro, Mexico

The manuscript by Mitra *et al *lays out a very interesting case for a connection between a  novel bacterial strain ( *Acidovorax* sp.) that was isolated from a contaminated TAP medium plate of Chlamydomonas and that can utilize common environmental pollutants as a carbon source. I also agree that the identification and characterization of *Acidovorax *sp., is absolutely required to reduce prevalence and prevent the further spread of contamination on TAP medium.

 The work is well performed and easy to follow. I think that this adds to the body of knowledge and recommend for acceptance with minor comments addressed below:

 Please see below for individual points:

1. Figure 2 could be eliminated or improve the quality of the figure.
2. I suggest changing the figure 8 for a graphic or table.
3. In page. 17, first line change LMG for LMJ.

Is the work clearly and accurately presented and does it cite the current literature?

Yes

If applicable, is the statistical analysis and its interpretation appropriate?

Yes

Are all the source data underlying the results available to ensure full reproducibility?

Yes

Is the study design appropriate and is the work technically sound?

Yes

Are the conclusions drawn adequately supported by the results?

Yes

Are sufficient details of methods and analysis provided to allow replication by others?

Yes

Reviewer Expertise:

Molecular microbiology, RNA expression, tRNA methabolism.

I confirm that I have read this submission and believe that I have an appropriate level of expertise to confirm that it is of an acceptable scientific standard.

# Peer review: Reviewer response for version 1

**DOI:** 10.5256/f1000research.27224.r65744

**Published:** 2020-08-12

**Authors:** Ruby A Ynalvez

**Affiliations:** 1 Department of Biology and Chemistry, Texas A&M International University, Laredo, TX, USA

This paper gave a report on a novel bacterium that share morphological and many biochemical traits with *Acidovorax* sp. I find the paper interesting and worthy of publishing.

1. The isolation of this bacteria was highly interesting as the work in this article stemmed from a side research project of young investigators. There were four undergraduates and, one high school student.
2. The bacterium being reported is a promising candidate for further investigation for its use in bioremediation.
3. The bacterium produces pyomelanin. This finding opens another avenue for investigation - - to probe pyomelanin’s role in oxidative stress protection in LMJ. 

Sufficient and scientifically sound methods have been done in order to characterize this novel bacterial strain. Results presented were adequate to give detailed characterization of the novel isolate. In addition, I appreciate that the authors provided sufficient and clear details of methods and analysis to allow replication by others.

 This paper reported an interesting finding that has a potential for useful application in the future. I hope that the authors can continue to further characterize this novel strain, LMJ.

 I just have few and minor comments:

1. The optical density of 0.08–13 which matches the 0.5 McFarland standard seem to be a very wide range and 13 is too high. I think this is a typographical error.
2. Is there a need to present the data from each replication? Consider presenting just the means and SDs and mentioned in the methods and/or underneath the table the number of replications done. 

Is the work clearly and accurately presented and does it cite the current literature?

Yes

If applicable, is the statistical analysis and its interpretation appropriate?

Yes

Are all the source data underlying the results available to ensure full reproducibility?

Yes

Is the study design appropriate and is the work technically sound?

Yes

Are the conclusions drawn adequately supported by the results?

Yes

Are sufficient details of methods and analysis provided to allow replication by others?

Yes

Reviewer Expertise:

Molecular Biology and Biochemistry

I confirm that I have read this submission and believe that I have an appropriate level of expertise to confirm that it is of an acceptable scientific standard.

# Peer review: Reviewer response for version 1

**DOI:** 10.5256/f1000research.27224.r69019

**Published:** 2020-08-10

**Authors:** Dickson Aruhomukama

**Affiliations:** 1 Department of Medical Microbiology, College of Health Sciences, Makerere University, Kampala, Uganda

First, I would like to commend the authors for the good work. I find the work very interesting and comprehensive in several aspects.

 Second, I find every pursuit of identifying candidates that could be used in bioremediation of great importance.

 Third, I commend the authors for exploring to the dot a variety of methods in this work.

 I hope that the authors can sequence the bacterial strain and continue to pursue this work to further understand its role in bioremediation.

Is the work clearly and accurately presented and does it cite the current literature?

Partly

If applicable, is the statistical analysis and its interpretation appropriate?

Yes

Are all the source data underlying the results available to ensure full reproducibility?

Yes

Is the study design appropriate and is the work technically sound?

Yes

Are the conclusions drawn adequately supported by the results?

Yes

Are sufficient details of methods and analysis provided to allow replication by others?

Yes

Reviewer Expertise:

Microbiology, Microbial Genomics and Bioinformatics, Biotechnology

I confirm that I have read this submission and believe that I have an appropriate level of expertise to confirm that it is of an acceptable scientific standard.
